# DFT exchange: sharing perspectives on the workhorse of quantum chemistry and materials science

**DOI:** 10.1039/d2cp02827a

**Published:** 2022-08-10

**Authors:** Andrew M. Teale, Trygve Helgaker, Andreas Savin, Carlo Adamo, Bálint Aradi, Alexei V. Arbuznikov, Paul W. Ayers, Evert Jan Baerends, Vincenzo Barone, Patrizia Calaminici, Eric Cancès, Emily A. Carter, Pratim Kumar Chattaraj, Henry Chermette, Ilaria Ciofini, T. Daniel Crawford, Frank De Proft, John F. Dobson, Claudia Draxl, Thomas Frauenheim, Emmanuel Fromager, Patricio Fuentealba, Laura Gagliardi, Giulia Galli, Jiali Gao, Paul Geerlings, Nikitas Gidopoulos, Peter M. W. Gill, Paola Gori-Giorgi, Andreas Görling, Tim Gould, Stefan Grimme, Oleg Gritsenko, Hans Jørgen Aagaard Jensen, Erin R. Johnson, Robert O. Jones, Martin Kaupp, Andreas M. Köster, Leeor Kronik, Anna I. Krylov, Simen Kvaal, Andre Laestadius, Mel Levy, Mathieu Lewin, Shubin Liu, Pierre-François Loos, Neepa T. Maitra, Frank Neese, John P. Perdew, Katarzyna Pernal, Pascal Pernot, Piotr Piecuch, Elisa Rebolini, Lucia Reining, Pina Romaniello, Adrienn Ruzsinszky, Dennis R. Salahub, Matthias Scheffler, Peter Schwerdtfeger, Viktor N. Staroverov, Jianwei Sun, Erik Tellgren, David J. Tozer, Samuel B. Trickey, Carsten A. Ullrich, Alberto Vela, Giovanni Vignale, Tomasz A. Wesolowski, Xin Xu, Weitao Yang

**Affiliations:** School of Chemistry, University of Nottingham, University Park Nottingham NG7 2RD UK andrew.teale@nottingham.ac.uk; Hylleraas Centre for Quantum Molecular Sciences, Department of Chemistry, University of Oslo P.O. Box 1033 Blindern N-0315 Oslo Norway trygve.helgaker@kjemi.uio.no andre.laestadius@kjemi.uio.no e.i.tellgren@kjemi.uio.no simen.kvaal@kjemi.uio.no; Laboratoire de Chimie Théorique, CNRS and Sorbonne University 4 Place Jussieu CEDEX 05 75252 Paris France andreas.savin@lct.jussieu.fr; PSL University, CNRS, ChimieParisTech-PSL, Institute of Chemistry for Health and Life Sciences, i-CLeHS 11 rue P. et M. Curie 75005 Paris France carlo-adamo@chimie-paristech.fr ilaria.ciofini@chimie-paristech.fr; Bremen Center for Computational Materials Science, University of Bremen P.O. Box 330440 D-28334 Bremen Germany aradi@uni-bremen.de thomas.frauenheim@bccms.uni-bremen.de; Technische Universität Berlin, Institut für Chemie, Theoretische Chemie/Quantenchemie, Sekr. C7 Straße des 17. Juni 135 10623 Berlin alexey.arbuznikov@tu-berlin.de martin.kaupp@tu-berlin.de; McMaster University Hamilton Ontario Canada ayers@mcmaster.ca; Department of Chemistry and Pharmaceutical Sciences, Faculty of Science, Vrije Universiteit De Boelelaan 1083 1081HV Amsterdam The Netherlands e.j.baerends@vu.nl; Scuola Normale Superiore Piazza dei Cavalieri 7 56125 Pisa Italy vincenzo.barone@sns.it; Departamento de Química, Centro de Investigación y de Estudios Avanzados (Cinvestav) CDMX 07360 Mexico akoster@cinvestav.mx avela@cinvestav.mx pcalamin@cinvestav.mx; CERMICS, Ecole des Ponts and Inria Paris 6 Avenue Blaise Pascal 77455 Marne-la-Vallée France cances@cermics.enpc.fr; Department of Mechanical and Aerospace Engineering and the Andlinger Center for Energy and the Environment, Princeton University Princeton NJ 08544-5263 USA eac@princeton.edu; Department of Chemistry, Indian Institute of Technology Kharagpur 721302 India pkc@chem.iitkgp.ac.in; Institut Sciences Analytiques, Université Claude Bernard Lyon1 CNRS UMR 5280 69622 Villeurbanne France henry.chermette@univ-lyon1.fr; Department of Chemistry, Virginia Tech Blacksburg VA 24061 USA crawdad@vt.edu; Molecular Sciences Software Institute Blacksburg VA 24060 USA; Research Group of General Chemistry (ALGC), Vrije Universiteit Brussel (VUB) Pleinlaan 2 B-1050 Brussels Belgium fdeprof@vub.be pgeerlin@vub.be; Griffith University Nathan Queensland 4111 Australia j.dobson@griffith.edu.au; Institut für Physik and IRIS Adlershof, Humboldt-Universität zu Berlin 12489 Berlin Germany claudia.draxl@physik.hu-berlin.de; Fritz-Haber-Institut der Max-Planck-Gesellschaft 14195 Berlin Germany; Beijing Computational Science Research Center (CSRC) 100193 Beijing China; Shenzhen JL Computational Science and Applied Research Institute 518110 Shenzhen China; Laboratoire de Chimie Quantique, Institut de Chimie, CNRS/Université de Strasbourg 4 rue Blaise Pascal 67000 Strasbourg France fromagere@unistra.fr; Departamento de Física, Facultad de Ciencias, Universidad de Chile Casilla 653 Santiago Chile pfuentea@hotmail.es; Department of Chemistry, Pritzker School of Molecular Engineering, The James Franck Institute, and Chicago Center for Theoretical Chemistry, The University of Chicago Chicago Illinois 60637 USA lgagliardi@uchicago.edu; Pritzker School of Molecular Engineering and Department of Chemistry, The University of Chicago Chicago IL USA gagalli@uchicago.edu; Institute of Systems and Physical Biology, Shenzhen Bay Laboratory Shenzhen 518055 China jiali@jialigao.org; Department of Chemistry, University of Minnesota Minneapolis MN 55455 USA; Department of Physics, Durham University South Road Durham DH1 3LE UK nikitas.gidopoulos@durham.ac.uk; School of Chemistry, University of Sydney Camperdown NSW 2006 Australia p.gill@sydney.edu.au; Department of Chemistry and Pharmaceutical Sciences, Amsterdam Institute of Molecular and Life Sciences (AIMMS), Faculty of Science, Vrije Universiteit De Boelelaan 1083 1081HV Amsterdam The Netherlands p.gorigiorgi@vu.nl o.gritsenko@vu.nl; Chair of Theoretical Chemistry, University of Erlangen-Nuremberg Egerlandstrasse 3 91058 Erlangen Germany andreas.goerling@fau.de; Qld Micro- and Nanotechnology Centre, Griffith University Gold Coast Qld 4222 Australia t.gould@griffith.edu.au; Mulliken Center for Theoretical Chemistry, University of Bonn Beringstrasse 4 53115 Bonn Germany grimme@thch.uni-bonn.de; Department of Physics, Chemistry and Pharmacy, University of Southern Denmark DK-5230 Odense M Denmark hjj@sdu.dk; Department of Chemistry, Dalhousie University Halifax Nova Scotia B3H 4R2 Canada erin.johnson@dal.ca; Peter Grünberg Institut PGI-1, Forschungszentrum Jülich 52425 Jülich Germany r.jones@fz-juelich.de; Department of Molecular Chemistry and Materials Science, Weizmann Institute of Science Rehovoth 76100 Israel leeor.kronik@weizmann.ac.il; Department of Chemistry, University of Southern California Los Angeles California 90089 USA krylov@usc.edu; Department of Chemistry, Tulane University New Orleans Louisiana 70118 USA mlevy@tulane.edu; CNRS & CEREMADE, Université Paris-Dauphine, PSL Research University, Place de Lattre de Tassigny 75016 Paris France mathieu.lewin@math.cnrs.fr; Research Computing Center, University of North Carolina Chapel Hill NC 27599-3420 USA shubin@email.unc.edu; Department of Chemistry, University of North Carolina Chapel Hill NC 27599-3290 USA; Laboratoire de Chimie et Physique Quantiques (UMR 5626), Université de Toulouse, CNRS UPS France loos@irsamc.ups-tlse.fr; Department of Physics, Rutgers University at Newark 101 Warren Street Newark NJ 07102 USA neepa.maitra@rutgers.edu; Max Planck Institut für Kohlenforschung Kaiser Wilhelm Platz 1 D-45470 Mülheim an der Ruhr Germany neese@kofo.mpg.de; Departments of Physics and Chemistry, Temple University Philadelphia PA 19122 USA perdew@temple.edu; Institute of Physics, Lodz University of Technology ul. Wolczanska 219 90-924 Lodz Poland pernalk@gmail.com; Institut de Chimie Physique, UMR8000, CNRS and Université Paris-Saclay Bât. 349 Campus d’Orsay 91405 Orsay France pascal.pernot@universite-paris-saclay.fr; Department of Chemistry, Michigan State University East Lansing Michigan 48824 USA piecuch@chemistry.msu.edu; Department of Physics and Astronomy, Michigan State University East Lansing Michigan 48824 USA; Institut Laue Langevin 71 avenue des Martyrs 38000 Grenoble France rebolini@ill.fr; Laboratoire des Solides Irradiés, CNRS, CEA/DRF/IRAMIS, École Polytechnique, Institut Polytechnique de Paris F-91120 Palaiseau France Lucia.Reining@polytechnique.fr; European Theoretical Spectroscopy Facility https://www.etsf.eu/; Laboratoire de Physique Théorique (UMR 5152), Université de Toulouse, CNRS UPS France pina.romaniello@irsamc.ups-tlse.fr; Department of Physics, Temple University Philadelphia Pennsylvania 19122 USA aruzsinszky@temple.edu; Department of Chemistry, Department of Physics and Astronomy, CMS – Centre for Molecular Simulation, IQST – Institute for Quantum Science and Technology, Quantum Alberta, University of Calgary 2500 University Drive NW Calgary Alberta T2N 1N4 Canada dsalahub@ucalgary.ca; The NOMAD Laboratory at the FHI of the Max-Planck-Gesellschaft and IRIS-Adlershof of the Humboldt-Universität zu Berlin Faradayweg 4-6 D-14195 Germany scheffler@fhi-berlin.mpg.de; Centre for Theoretical Chemistry and Physics, The New Zealand Institute for Advanced Study, Massey University Auckland 0632 Auckland New Zealand peter.schwerdtfeger@gmail.com; Department of Chemistry, The University of Western Ontario London Ontario N6A 5B7 Canada vstarove@uwo.ca; Department of Physics and Engineering Physics, Tulane University New Orleans LA 70118 USA jsun@tulane.edu; Department of Chemistry, Durham University South Road Durham DH1 3LE UK d.j.tozer@durham.ac.uk; Quantum Theory Project, Deptartment of Physics, University of Florida Gainesville FL 32611 USA trickey@qtp.ufl.edu; Department of Physics and Astronomy, University of Missouri Columbia MO 65211 USA ullrichc@missouri.edu; Department of Physics, University of Missouri Columbia MO 65203 USA vignaleg@missouri.edu; Department of Physical Chemistry, Université de Genève 30 Quai Ernest-Ansermet 1211 Genève Switzerland tomasz.wesolowski@unige.ch; Shanghai Key Laboratory of Molecular Catalysis and Innovation Materials, Collaborative Innovation Centre of Chemistry for Energy Materials, MOE Laboratory for Computational Physical Science, Department of Chemistry, Fudan University Shanghai 200433 China xxchem@fudan.edu.cn; Department of Chemistry and Physics, Duke University Durham NC 27516 USA weitao.yang@duke.edu

## Abstract

In this paper, the history, present status, and future of density-functional theory (DFT) is informally reviewed and discussed by 70 workers in the field, including molecular scientists, materials scientists, method developers and practitioners. The format of the paper is that of a roundtable discussion, in which the participants express and exchange views on DFT in the form of 302 individual contributions, formulated as responses to a preset list of 26 questions. Supported by a bibliography of 777 entries, the paper represents a broad snapshot of DFT, anno 2022.

## Abstract 2

In this paper, the history, present status, and future of density-functional theory (DFT) is informally reviewed and discussed by 70 workers in the field, including molecular scientists, materials scientists, method developers and practitioners.

## Graphical abstract

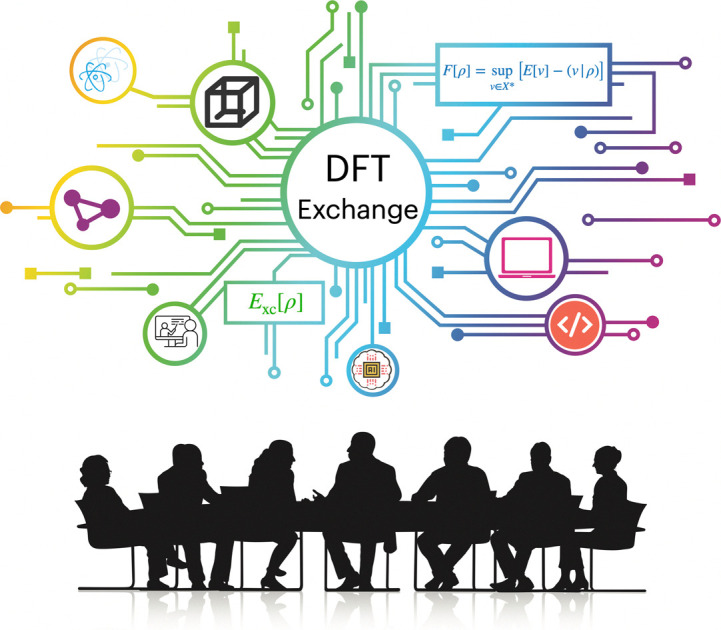

## 1 Introduction

What is the status of DFT? Where is DFT heading? What are the important new developments in DFT and what are the points of contention? *What is DFT?*

Such questions are discussed whenever developers and users of density-functional theory (DFT) meet – in conferences and workshops, during coffee breaks and over dinners. We do not expect short, clear answers to such questions but the discussions and conversations they give rise to are often informative and entertaining – and different from discussions in publications and presentations. We learn about new ideas and developments and about failed attempts – a casual remark may trigger new research or lead to new collaborations. These discussions are an important reason for travelling to conferences and something we have missed during the pandemic.

This article is an attempt to bring such discussions to the printed format – to let prominent workers in the field exchange views and thoughts about DFT in an open informal manner, mimicking the format of a roundtable discussion, but backing up their statements by arguments and references to the literature. The end result should be a lively guide to DFT and its development.

The format of the present article is an unusual one, resembling most closely the Faraday Discussions but not anchored to the talks presented at a conference. It is to our knowledge the first paper of its kind in PCCP and the first such paper on DFT. Given its unusual format, we here describe how it came about.

The initiative for the article was taken by three of the authors, Andy Teale, Trygve Helgaker, and Andreas Savin. Having received a go-ahead for the project from the publisher, the three initiators compiled an initial list of questions about DFT and some tentative answers. A letter of invitation was then sent out to about hundred workers in the field, inviting them “to participate in what will hopefully be an open, thought provoking and informal discussion about density-functional theory and its applications”. To clarify the format of the article, the invitation contained a link to the document with the preliminary questions and answers. A total of 67 accepted the invitation, bringing the number of authors to 70.

In a process involving all authors, the preliminary questions were revised and preliminary answers removed. A final set of 26 questions was agreed upon: five questions for DFT, nine for Density-Functional Approximations (DFAs), eight for The Future of DFT and DFAs, and four for Communicating and Sharing Our Results.

All authors were then invited by the initiators to contribute to the discussion by providing answers to the questions and also comments to answers over a six-week period, encouraging discussions among the authors. Guidelines were provided to ensure a smooth collaborative process. The end result was an extensive first draft of the manuscript, running over sixty pages and with several hundred references. After a two-week internal review involving all authors, an additional two weeks were allotted for responses to the internal review. The purpose of the internal review was solely to improve clarity of expression – not to restrict in any way the freedom of the authors to express their opinions.

The final draft was edited by the three initiators, with the aim of improving the organization of the manuscript by reordering contributions and comments, reducing, where possible, repetition and ensuring a certain level of uniformity in notation and clarity of presentation. However, to retain the spontaneity of the discussion and reflect the multitude of views presented, reorganization was kept to a minimum. As a consequence, some themes may be revisited in different contexts throughout the paper – much as would happen in a lively roundtable discussion.

Having received a final go-ahead from all co-authors, the final manuscript was submitted to the journal. All work on the paper was carried out with LaTeX, using the Overleaf platform^1^ for ease of collaboration.

The final manuscript provides an interesting snapshot of where DFT stands today and where it is moving. It covers much of DFT with an extensive bibliography, but coverage is nevertheless not exhaustive – classical DFT and multicomponent DFT are not discussed, for example. The topics covered in the paper reflect the interests of the authors. Also, the views stated are those of the individual authors – as such, the paper has no conclusion. In the spirit of the paper, you are instead encouraged to continue this exchange of views, by contacting the authors.

## 2 Density-functional theory

### 2.1 What is DFT?

#### 2.1.1 Savin

Density-functional theory (DFT) is more than existence theorems. I like to make the distinction between

(1) a density functional, a number obtained from the density;

(2) DFT, the collection of theorems useful for obtaining exact results with procedures using density functionals, without having to solve the exact many-body problem;

(3) the methods using them – for example, the Kohn–Sham method; and

(4) density-functional approximations (DFAs), the approximations (or models). The latter can originate from a choice of a “closed form”, as mentioned in contribution (2.1.4), or from controllable ones, as related to the numerical treatment and discussed in contribution (4.6.7).

#### 2.1.2 Levy

Federico Zahariev and I have recently shown in ref. 2 that it is useful and variationally valid to employ spin-free wave functions in the constrained-search formulation when deriving certain properties of a functional for the purpose of its approximation.

In the constrained-search formulation of pure-state (or ensemble) DFT, the kinetic plus electron–electron repulsion energy of a density is the expectation value of the wave function (or ensemble) that yields this density and minimizes the kinetic plus electron–electron repulsion expectation value. That is,1
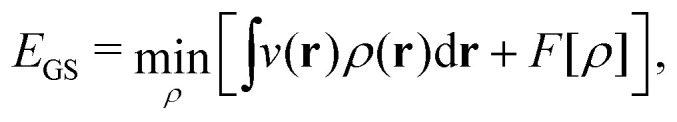
where, with the use of pure-state wave functions,2
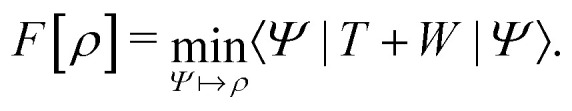
The wave functions are here spin-free, but antisymmetric in the first *M* spatial coordinates and separately antisymmetric in last (*N* − *M*) spatial coordinates. The generalization of *F*[*ρ*] to ensembles should be clear. This generalization ensures convexity.

#### 2.1.3 Reining

One may distinguish different possible aspects in this question: *What is the message of DFT? Why has it been successful? How is it used today? What distinguishes it from other theories that deal with the many-body problem?* Some are treated later, so I think we should focus on the first aspect here. I also think that, in answering this and many other questions, a glance at other possible theoretical approaches is healthy, because we always learn from comparison, so let us try to have such a point of view whenever possible.

The term DFT expresses the fact that observables in the ground state at zero temperature can be considered as functionals of the ground-state density. This can then be extended to thermal equilibrium, *etc.*, as others point out. So, it means that the density is a sufficient descriptor. It is important to say “*can be considered as* a functional of the density” and not “is a functional of the density”, because this is a choice: observables can also be considered as functionals of the many-body ground-state wave function, or the one-body Green's function, or many other possible choices. The functional of the many-body ground-state wave function is very simple (whereas the wave function is not, of course), and a density functional will in most cases be exceedingly complicated (whereas the density is simple). Actually, I chose to say “can be considered as”, because this does not imply that there must be an explicit expression.

A second important point: the density is not known *a priori* but is needed as input to evaluate our density functionals for a given system and observable. So, as a second aspect of DFT, we also have to invoke the variational character of the energy as functional of the density, because it allows us to find the density that is needed to evaluate the functionals for the various observables, without calculating the density from the many-body wave function. Otherwise, DFT could probably not compete with other approaches, not even as an idea – for example, also the external potential is a sufficient descriptor (for given particle number or chemical potential), it is simple, and it has the advantage that we (think we) know it. The variational character also has the benefit that a slightly wrong density may still lead to a reasonable energy (whereas this may not hold for other observables).

So, we may consider DFT as one possibility: one possible way to formulate the calculation of observables in a many-body system. There are many such ways, and we know that for most systems we will never be able to obtain the exact answer. Therefore, once we agree that those various ways are in principle exact, the true question is: how suitable are they as starting points for approximations? And so, for our purpose here: in which way is DFT a good starting point for approximations?

#### 2.1.4 Scheffler

Since the development of the quantum mechanics of atoms and polyatomic systems, it was clear that inspection of the ground-state electron density *ρ*(**r**) provides the information on the total number of electrons, *N*, the positions of the atoms, {**R**_*I*_}, and from *ρ*(**R**_*I*_) the nuclear charges.^3,4^ Thus, *ρ*(**r**) determines *N*, {**R**_*I*_}, {*Z*_*I*_} – that is, the many-electron Hamiltonian, and therefore, it determines everything. This is the algorithm that defines how to go from the ground-state density to the energy.

The theorem of Hohenberg and Kohn^5^ and the works by Levy^6,7^ and Lieb^8^ are beautiful mathematical treatments. Importantly, the basic concept that the ground-state electron density determines everything often enables decisive physical insight. The often misleading assumption is that the above laid out, exact algorithm “*ρ*(**r**) 
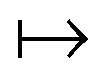
 ground-state energy (and even everything)” can be expressed in terms of a closed mathematical expression. Approximating the algorithm by a mathematical functional, *i.e.*, by a DFA, suffers from the severe problem that the range of validity of this functional is typically unclear: We can test its accuracy only by comparing results with experiments or high-level wave-function theories. We trust the reliability for systems that we believe (!) are “similar” to the tested ones, but we don’t know about the accuracy for untested systems. And the term “similar” is not even defined.

Let me add: I am not aware of a proof that the exact exchange–correlation-functional exists, beyond the noted algorithm which requires to solve the many-body Schrödinger equation. However, and most importantly, the works by Hohenberg and Kohn and Kohn and Sham have shown the way to develop density-functional approximations which revolutionized the description and understanding of polyatomic systems.

#### 2.1.5 Kvaal

I agree with Savin in contribution (2.1.1) – in particular with respect to the claim that a distinction between exact DFT and approximate DFT is useful. In my opinion, they are both conceptually and mathematically different. They share the use of the density and potential as dual basic variables, but otherwise the similarities disappear for me. For instance, a DFA will have much nicer mathematical properties than the exact universal functional, as they are built from simple, explicit ingredients, at least partially necessitated by the need for efficient numerical evaluation and optimization in order to be useful. On the other hand, the exact universal density functional has a complicated implicit definition, leading to a highly complicated functional. A concrete formulation of this is due to Schuch and Verstraete,^9^ who demonstrated that, if an efficient evaluation of the universal functional could be done, all NP hard problems would be solvable in polynomial time. This is highly unlikely. On the other hand, DFAs are necessarily computable! (It is of course one of the marvels of DFT, that it is even possible to obtain such good results with so little computational effort.)

Thus, approximate and exact density-functionals are mathematically quite different. The noncomputability of the exact functional indicates that systematically improvable DFAs are probably possible, in the sense of mathematical *a priori* error estimation – that is, mathematical statements towards an approximation's accuracy in terms of its adjustable parameters, such as basis size. Therefore, I would like to go out on a limb and say that approximate density functionals are not really approximations to exact density functionals. They are instead largely independent and, to a variable extent, semiempirical models that have the common use of the density as a basic variable as a characteristic. The latter aspect is for me an answer to the question “What is DFT?”

#### 2.1.6 Savin

Let me comment on the difficulty of obtaining exact functionals in a (semi)local form by choosing a simpler example. The Hartree density functional,3
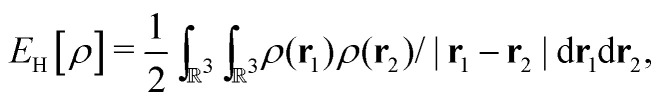
is universal, and not only known but also simple. However, I don’t see how to replace it by a (semi)local form.††Note that there is a (semi)local form for short-range interactions, *e.g.*, *δ*(**r**_1_ − **r**_2_), 
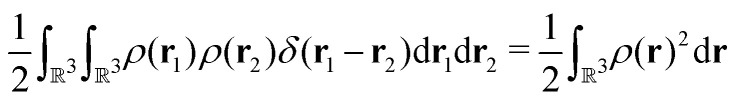
. One can argue that this does not lead to problems, as we compute *E*_H_ explicitly. However, this argument is not valid if we choose to express the exchange functional, *E*_x_, in a (semi)local form: for one-electron systems, *E*_x_ = −*E*_H_.

#### 2.1.7 Yang

I agree with Savin on the difficulty of semilocal functionals. The example of the interaction energy of a one-electron system is a clear case: the exact exchange–correlation energy has to cancel the classical Coulomb energy.^10^ Otherwise, the functional has a self-interaction error (SIE).

For many years, the SIE had been assumed to be the main systematic error in DFAs, related to the incorrect dissociation of molecular ions, the underestimation of chemical reaction barriers and band gaps of molecules and bulk materials, the overestimation of polymer polarizability, and many other failure of commonly used DFAs.^11,12^ However, the development of two SIE-free functionals, the Becke05^13^ and the MCY2^14^ functionals, changed the understanding.^15^ While these two exchange–correlation functionals, nonlocal and also nonsemilocal, are SIE-free by construction for any one-electron system and perform as well on thermodynamics benchmarks as hybrid functionals, they still retain significant errors in the dissociation of molecular ions, band gaps of molecules, and polymer polarizability problems, much like the hybrid functional B3LYP. The only significant improvement observed is in the prediction of reaction barriers. Thus the systematic error is clearly not the SIE.

To describe the systematic error of DFAs, the concept of the delocalization error has been developed, and it can be understood from the perspective of fractional charges.^16,17^ For systems of small or moderate physical sizes, conventional DFAs usually have good accuracy in total energies for an integer number of electrons. For a fractional number of electrons, conventional DFAs, however, violate the Perdew–Parr–Levy–Balduz (PPLB) linearity condition,^18–20^ which states that the exact ground-state energy *E*(*N*) is a linear function of the fractional electron numbers connecting adjacent integer points. Inconsistent with the requirement of the PPLB linearity condition, *E*(*N*) curves from conventional DFAs are usually convex, with drastic underestimation to the ground-state energies of fractional systems. The convex deviation of conventional DFAs decreases when the systems become larger and vanishes at the bulk limit. However, the delocalization error is exhibited in another way, in which the error manifests itself in too low relative ground-state energies of ionized systems and incorrect linear *E*(*N*) curves with wrong slopes at the bulk limit.^16,17,21^

To reduce or eliminate the delocalization error, enormous efforts have been devoted to the development of new exchange–correlation functionals. None of these developments are based on a semilocal form. All have nonlocal features in the functionals – see the development of the scaling approaches.^22–25^

In addition to the delocalization error characterized by fractional charges, commonly used DFAs also have a significant systematic static correlation error characterized by the violation of the constancy conditions on fractional spins.^17,20,26^ The combination of the exact fractional charge condition^18^ and the exact fractional spin condition^20,26^ leads to the general flat-plane condition,^27^ the satisfaction of which is a necessary condition for describing the band gap of strongly correlated Mott insulators. The flat-plane condition also leads to the conclusion that the exact exchange–correlation functional cannot be a continuous functional of the electron density or the density matrix of the noninteracting reference system everywhere.^27^ To reduce or eliminate the static correlation error, one has to use nonlocal functionals.^28^

#### 2.1.8 Savin

Warren Pickett said during a talk (Brisbane, 1996): “True, the density gives the potential, and this makes the Hohenberg–Kohn theorem sound so empty, because the potential, we know it anyhow”. We do not need to start with an unknown function, *ρ*(**r**), when it is equivalent to using a known function of the position **r** – namely, the external potential, *v*(**r**).

#### 2.1.9 Trickey

The Pickett remark quoted by Savin is a paraphrase of the analysis that Per-Olov Löwdin had attributed earlier to E. Bright Wilson.^29^ The density cusps tell you the nuclear charges, hence the external potential *v*, hence the Hamiltonian. Also see Krylov's contribution (2.1.22) below.

#### 2.1.10 Yang

The Hohenberg–Kohn work established the principles for describing a many-electron system from the reduced variable of its electron density and the Kohn–Sham work provided the formulation to use a noninteracting reference system to represent the electron density of a many-electron system. These works are the solid foundation of DFT. However, they do not lead to any systematic pathway to the approximation of the density functional; see contribution (2.1.8). The specific approximations for the density functionals are the key to all applications.

#### 2.1.11 Helgaker

I suppose the nontrivial result is that (for a given number of electrons) the potential and density are dual variables – what you can calculate from one, you can calculate from the other. In particular, we can calculate the energy directly from the density, bypassing the potential.

#### 2.1.12 Yang

Indeed, the dual formulation of DFT is the potential-functional theory (PFT).^30^ PFT establishes two results: the dual of the Hohenberg–Kohn theorem in terms of the external potential as the basic variable and the dual of the Kohn–Sham theorem in terms of the potential of the noninteracting reference system. The first result provides a solution to the *v*-representability problem in the original Hohenberg–Kohn work. The second result provides the theoretical foundation for the optimized-effective-potential approach for Kohn–Sham calculations with functionals of orbitals.

#### 2.1.13 Helgaker

I like to think of DFT in terms of Legendre–Fenchel transforms.^8,31^ In short, from the concavity and continuity of the ground-state energy 
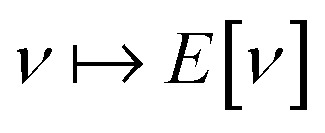
 as a function of the external potential *v* ∈ *L*^3/2^(ℝ^3^) + *L*^∞^(ℝ^3^) follows the existence of a universal density functional 
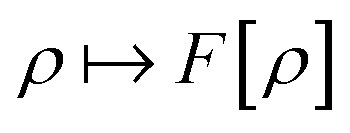
 as a function of the electron density *ρ* ∈ *L*^3^(ℝ^3^) ∩ *L*^1^(ℝ^3^) such that^5,8^4
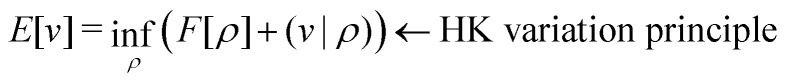
5
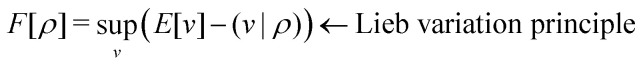
where 
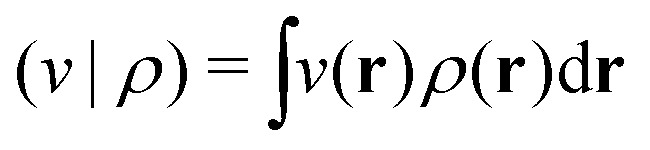
. Since *E* and *F* can be calculated from each other, they contain the same information, only expressed in different ways. However, although the Lieb variation principle is a powerful tool for analysis and method development, it is not a practical tool for computation. Instead, the power of DFT derives from Kohn–Sham theory, making it possible to approximate *F*[*ρ*] (sufficiently) accurately and inexpensively for densities *ρ* of interest to us by introducing orbitals.

#### 2.1.14 Levy

In contribution (2.1.13), Helgaker states that he prefers the Legendre-transform formulation. However, it has been shown that the Legendre-transform formulation is equivalent to the ensemble constrained search.^8^

#### 2.1.15 Helgaker

It is of course correct that the ensemble constrained-search functional is identical to Lieb's functional. With respect to the different formulations of DFT, my view is the following.

The Hohenberg–Kohn theorem,^5^ often thought of as the cornerstone of DFT, is easy to prove (apart from some subtleties) but perhaps not so easy to understand intuitively. Hohenberg and Kohn's original formulation of DFT is therefore not only restrictive in scope (in that it assumes *v*-representability) but may also appear a little mysterious.

Levy's constrained-search formulation^6^ took the mystery out of DFT and brought clarity and generality to the field – a major step forward, indeed. Lieb's convex formulation,^8^ on the other hand, gave DFT beauty and elegance by identifying the density functional with the Legendre transform (convex conjugate) of the ground-state energy, thereby placing DFT in a broader mathematical framework.^32^

It is an important and nontrivial result in DFT that the ensemble constrained-search functional and the Legendre-transform functional are the same – they are merely complementary formulations of the same thing.^8^ Together, they constitute the solid foundation of DFT.

#### 2.1.16 Scheffler

I somehow disagree with the last sentence of contribution (2.1.13). Clearly, Kohn–Sham theory has provided us with significant understanding, for polyatomic systems, mostly for cases where the physics is largely governed by the independent-particle kinetic-energy operator (or its orbitals). However, in general, I would hesitate to call Kohn–Sham theory together with the known DFAs “(sufficiently) accurate”. A key scientific problem is that the range of validity of the known DFAs is unknown, and a reliable estimate of the accuracy and a systematic convergence of the accuracy are not possible. Our own pragmatic approach is to perform calculations with different DFAs, and if the results are similar, we tend to accept them. Otherwise, we are worried. And, if possible, we check final results by a higher-level theory – by, for example, coupled-cluster theory.

#### 2.1.17 Kvaal

It is interesting to note that Lieb's convex formulation of exact DFT, the essence of which is succinctly described in contribution (2.1.13), does not rely in any way on the classical Hohenberg–Kohn theorems to establish duality of *ρ* and *v*. Neither are the theorems necessary for the derivation of exact Kohn–Sham theory. While the original Hohenberg–Kohn theorems are now established rigorously, albeit with mild assumptions on the potential,^33^ it is my opinion much easier to say that the Legendre transform of *E*[*v*] is the essence and foundation of DFT, from both a mathematical and a physical point of view. Lammert has pointed out that the Hohenberg–Kohn density-potential correspondence map is quite ill-behaved.^34^ Nearby *v*-representable densities may have wildly different potentials, and thus fundamental arguments that rely on, for example, some kind of differentiation of *v* as a function of *ρ* are not useful, at least for exact DFT.^34^

#### 2.1.18 Laestadius

With recent development of unique-continuation from sets of measure zero, in particular by Garrigue,^35^ I regard the Hohenberg–Kohn theorem as rigorous, albeit with some limitations. In particular, certain *L*^*p*^ spaces need to be considered for the potentials – for example, Theorem 30 in ref. 33 is a Hohenberg–Kohn result with all previous gaps filled, although it is not given for *L*^3/2^ + *L*^∞^.

Furthermore, comparing the situation with paramagnetic-current DFT, where the lack of a (corresponding) Hohenberg–Kohn theorem has been established by Capelle and Vignale,^36^ it is striking that although (*ρ*,**j**_p_) determines the nondegenerate ground state, if degeneracies are allowed, then the level of degeneracy is not determined.^37^ A given (*ρ*,**j**_p_) can therefore be associated with two different Hamiltonians (in fact, infinitely many) that may have different numbers of degenerate ground states. (Of course, this doesn’t stop the constrained search, which remains well defined.) In DFT, the extra layer of a Hohenberg–Kohn theorem (not just the first part of a constrained search) rules out such situations. I view the Hohenberg–Kohn theorem as a gold reserve – it is perhaps unexciting and just sits in the vault but is, on the other hand, good to have in certain extreme situations.

#### 2.1.19 Helgaker

Regarding the Hohenberg–Kohn theorem in DFT, it is interesting to see what role it plays within the Legendre–Fenchel formulation of DFT. The condition for a minimizing density in the Hohenberg–Kohn variation principle as given in contribution (2.1.13) is −*v* ∈ ∂*F*[*ρ*] where ∂*F*[*ρ*] is the subdifferential of *F* at *ρ* – that is, the collection of potentials with ground-state density *ρ*. Likewise, the condition for a maximizing potential in the Lieb variation principle is *ρ* ∈ ∂*E*[*v*], where the subdifferential of *E* at *v* is the collection of all ground-state densities of *v*. In fact, the two conditions are equivalent:

6 *E*[*v*] = *F*[*ρ*] + (*v*|*ρ*) ⇔ −*v* ∈ ∂*F*[*ρ*] ⇔ *ρ* ∈ ∂*E*[*v*].

By the Hohenberg–Kohn theorem, the optimality condition of the Hohenberg–Kohn variation principle takes the form7
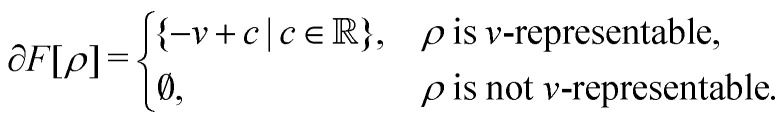
This uniqueness of the potential (up to an additive constant) is not mission critical for DFT but tells us that there is a unique maximizing potential in the Lieb variation principle (if any).

The optimality conditions in eqn (6) give some additional insight: the ground-state energy *E* and the universal density functional *F* are functions whose subdifferential mappings (“functional derivatives”) are each other's inverses. Loosely speaking, therefore, *E* and *F* may be obtained from each other by differentiation followed by inversion and integration.

#### 2.1.20 Salahub

Savins answer in contribution (2.1.1) to “what is DFT?” appeals to me because of its breadth. DFT appeals to different people for different reasons, from the joy of pure theory, to the satisfying hard work of DFAs, to the romp of applications across disciplines (when it works), to the agony when it doesn't (appealing to masochists, but also affording the possibility of looping back for improvements). So “DFT” is like an excellent marketing logo, as recognizable to scientists as the Nike logo is to the general public. Reasons for buying into DFT are numerous and varied, as reflected in the sections of this paper.

#### 2.1.21 Fuentealba

The first time I heard about DFT was in the eighties in Germany, and people called it “Density Functional Method”, because the theory is the quantum mechanics and one cannot have a theory into another one.

#### 2.1.22 Krylov

I first learned about the key ideas behind DFT before its modern incarnation was developed. Back in the eighties, chemists were using the X*α* method, which was regarded by *ab initio* theorists as semiempirical and, therefore, inferior to the then gold standard – the full Hartree–Fock method. We were struggling to understand why an inferior method would give more accurate results. I think the real insight was to understand that the Wilson conjecture – the observation that the one-electron density contains all the information needed to reconstruct the many-body Hamiltonian (and, therefore, to find the exact solution of the Schrödinger equation) – provides a physical justification for the existence of a mapping between the density and the exact energy of the system. The Hohenberg–Kohn theorems inform us that this mapping is unique.

With such justification, one can approach the problem of finding this mapping in a completely different way – not by building approximations to the known exact solution (as done in the wave-function theory), but by parameterizing an empirical representation of the mapping device, the functional. Most DFAs are built upon mathematical representations of the functional grounded in our physical understanding of what it should look like (based on exact results for model systems), but one can envision finding the mapping without any such help from physics – for example, by brute-force training of a neural network (machine learning).^38^ One can, therefore, think of DFT as an empirical method that can be made exact.

While the blind brute-force (*e.g.*, *via* ML) discovery of the density-energy mapping is, in principle, possible, it has important limitations compared to physically motivated DFAs. First, without any constraints due to physics, such brute-force search is going to be computationally wasteful. Second, having discovered the mapping between energy and density, one still has no recipe for computing energy derivatives with respect to various perturbations (*i.e.*, properties), unless properties (or various energy derivatives) were included in the training. In contrast, using a physically motivated form of the functional opens access to properties (although the quality is not guaranteed, as illustrated by the developments of magnetic DFAs^39^).

#### 2.1.23 Helgaker

I am not so fond of the Wilson conjecture – it works only if we already know that the potential is a Coulomb potential. It is a striking observation, but to some extent it trivializes DFT. The Hohenberg–Kohn theorem makes no such assumptions regarding the potential.

#### 2.1.24 Jones

A fixation on exact energies appears to be so strong among chemists that it justifies any amount of data fitting, so reducing DFT to a “semiempirical” or “empirical” method. With their focus on extended systems, materials scientists know that new knowledge can result from DFT calculations, even if all the calculated energies are wrong. See also contribution (2.2.23).

#### 2.1.25 Ayers

Arguably, any electronic structure theory method can be reformulated as a DFA by substituting its associated energy functional into the Legendre transform or its associated wave-function ansatz into the constrained search. So Hartree–Fock may be legitimately considered a DFT (a generalized Kohn–Sham DFT). Is Hartree–Fock theory and its analysis therefore DFT? Clearly, many coupled-cluster and propagator methods are also frequently analysed as DFT. I would not like to define DFT as “the sort of stuff that is done by density-functional theorists” but some work that is marketed as DFT (*cf.* contribution (2.1.20)) is not presented in the context of the mathematical framework of DFT (*cf.* contribution (2.1.1)).

To me, only orbital-free DFT is unequivocally DFT; everything else can also be fruitfully viewed from an alternative perspective. Indeed, some theoretical approaches and computational methods can legitimately be considered wave-function theories/methods, density-matrix theories/methods, propagator theories/methods, and density-functional theories/methods. I do not wish to take a hard line and proclaim that these types of theories/methods are not DFT because the philosophy (especially the emphasis on explicitly defining and characterizing the functional that is being approximated), traditions (especially the openness to pragmatic parameterization and approximation), and tools of DFT can be useful even for theories/methods that are “not just DFT”. But other, non-DFT, approaches could sometimes be even more useful.

#### 2.1.26 Görling

While the electron density certainly is a key quantity in DFT, I feel that there is a too strong focus on it – in particular, on the idea of getting the total energy or other information directly from the density. While this is the idea behind certain flavours of orbital-free DFT, it is not the idea behind the most commonly used DFT approaches, which are the Kohn–Sham or generalized Kohn–Sham methods. For these methods, a quite different view on DFT can be taken: to consider the electron density as the quantity that enables one to associate the real electronic system with a model system that has the same ground-state density, which makes it possible to describe the ground-state energy and other properties of the real system *via* the model system, *i.e.*, *via* its orbitals and eigenvalues. From the Kohn–Sham orbitals, traditionally, only the ‘noninteracting’ kinetic energy is calculated exactly, while the exchange–correlation energy is approximated by an explicit functional of the density.

But this is just one strategy. It is possible to determine additionally other contributions to the energy from the orbitals – for example, parts of the exchange energy in hybrid methods – or even to calculate all contributions to the energy exactly from the occupied orbitals, except the correlation energy. The latter can then be approximated by orbital-dependent functionals.^40^ In the latter case, the density is not needed at all in the calculation of the total DFT energy. If, furthermore, the orbitals are obtained *via* the optimized-effective-potential (OEP) method^40–46^ or within an appropriate generalized Kohn–Sham approach, then DFT methods results that do not require at any point the calculation of the density. The density is then only required in the underlying formalism.

I feel that the perception of DFT has been somewhat blurred by a questionable statement that, one way or another, is frequently found in textbooks and articles. This is the statement that DFT is distinguished from wave-function methods by using the electron density instead of a wave function to calculate the total energy of an electronic system. This statement is at least misleading if not wrong because most DFT methods used in practice are Kohn–Sham or generalized Kohn–Sham methods, which require orbitals and thus one-electron wave functions to calculate crucial parts of the total energy.

#### 2.1.27 Gidopoulos

I believe the distinction in the literature between wave-function methods and DFT is slightly different. In my understanding, the distinction is not that in DFT the energy is actually calculated from the density, once we know the density, because the question remains how to find the density. Rather, the distinction is that in DFT the solution to the electronic-structure problem is obtained by minimizing a total energy as a functional of the density, while in wave-function theory the solution is obtained by solving Schrödinger's equation. So, calculating the energy from the density does not mean literally plugging the density into some orbital-free expression, but the process of minimization of the total-energy density functional to obtain the minimum value, which is the total energy of the interacting system.

#### 2.1.28 Chattaraj

Any theory that applies density to understand a many-particle system, without using the exact wave function, can be termed as DFT.^47–49^ According to Hohenberg–Kohn theorems,^5^ DFT is a theory that legitimizes the use of the density to calculate all possible properties. The Hohenberg–Kohn theorems are just existence theorems and do not provide any know-how for an explicit form of the energy as a functional of the density as well as functional forms of other properties.

#### 2.1.29 Trickey

The foregoing discussion seems a bit parochial – for example, the identification in contribution (2.1.4) of DFT with “ground state”. That restriction seems to have been accepted by subsequent commentators in this section. But there are several instances of what generically is a DFT. There is, for example, a well-developed classical DFT. Closer to the focus of this discussion (many-fermion systems), there is free-energy DFT (also known as finite-temperature DFT).^50^ It inexorably involves excited states. There has been progress on free-energy DFAs.^51–56^ Another ensemble DFT is the Gross–Kohn–Oliveira (GOK) approach for excited states at *T* = 0 K (see other commentators below).

The common theme of these DFTs is the reduction of the inherent complexity of the direct description of a many-body system to the comparative simplicity of functionals of the density – either explicitly, or implicitly in terms of auxiliary functions such as orbitals. The strategy, in the time-independent case at least, is to obtain the relevant physics (hence also chemistry) by an appropriate minimization procedure on a functional of the density itself (whether it be pure-state or ensemble).

#### 2.1.30 Galli

In the Hohenberg–Kohn formulation, DFT is an exact theory of ground and excited states, entirely based on the electron density. That is, the density determines uniquely the potential, hence both ground and excited state properties of the system may in principle be derived. However there is no practical recipe on how to derive such potential and hence on how to derive neither ground or excited state properties. The Kohn–Sham formulation, in contrast, is applicable only to ground-state properties, although in practice it is applied also to excited states.

#### 2.1.31 Schwerdtfeger

We should be reminded that the charge density *ρ*(**r**, *t*) is not Lorentz invariant. As relativistic quantum (field) theory demands a fully covariant formalism, we have to use the four-current density *j*^*μ*^ as a function of the four-position *x*^*μ*^ instead of the charge density, the latter appearing only as the time-like (first) component of the four-vector (*ρ*, *j*_*x*_/*c*, *j*_*y*_/*c*, *j*_*z*_/*c*), where *c* is the speed of light in vacuum. The Hohenberg–Kohn theorem has been generalized to the relativistic domain by Rajagopal and Callaway^57^ and field-theoretical aspects have also been taken into account by Engel.^58^ Beside this enormous progress on the theoretical side, it is fair to say that applications in this most rigorous relativistic framework using the current-dependent exchange–correlation energy functional are more or less absent.^58^ The main reason lies, as one can guess, in the fact that relativistic DFT (RDFT) faces exactly the same fundamental problems as DFT in the nonrelativistic domain. As we know, relativistic effects can be very large for electronic properties of compounds containing heavy elements, often larger than the error introduced by many DFAs, thus justifying the introduction of the exchange–correlation functional into the (no-pair) Dirac–Coulomb (DC) equation (the Douglas–Kroll Kohn–Sham scheme) or into its corresponding two-component (such as exact two-component [X2C]) or scalar relativistic schemes, with or without the relativistic pseudopotential approximation. The latter together with DFT is clearly the main workhorse in solid-state physics. One may however, question the inclusion of smaller radiative QED corrections into RDFT as it cannot compete with more accurate wave-function based methods. On the other hand, we should mention that RDFT approximations based on the density *ρ* and the (noncollinear) magnetization density **m**^59,60^ have now become feasible and useful in many applications.

#### 2.1.32 Tellgren

In my view, a lot of work remains to be done on the theoretical side of RDFT too. Every rigorous formulation of nonrelativistic ground-state DFT depends on the ground state being identified as a global energy minimum. At the relativistic level, an energy minimization principle strong enough to construct a DFT is missing and the present attempts to establish a relativistic Hohenberg–Kohn theorem are not rigorous.

#### 2.1.33 Gritsenko

DFT can be formally considered as the result of the simplest exact functional closure of the conventional expression for the nonrelativistic ground-state energy *E*[*ρ*, *γ*, *P*], which includes the electron density *ρ*, the first-order reduced density matrix (1RDM) *γ*, and the diagonal part *P* of the 2RDM *Γ* corresponding to a ground-state wave function *Ψ*. This can be achieved in the spirit of the Bogoliubov–Born–Green–Kirkwood–Yvon (BBGKY) chain^61^ of the quantum dynamical reduced theories of many-electron systems. Truncation of the BBGKY chain with its exact or approximate closure at the *m*th level produces theories that operate with the *m*th (and lower) order RDMs.^62^ In this sense, DFT can be considered as the result of the exact closure at the “zero” (*i.e.*, only density functional) level of *E*[*ρ*, *γ*, *P*] with two maps, in complete analogy with those employed in the derivation of time-dependent density-matrix-functional theory (TDDMFT).^63^ The first map is the evident map *P* ← *Γ*
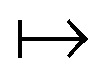
*γ*
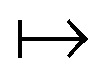
*ρ*, while the second map *ρ*
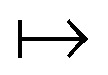
*Ψ*
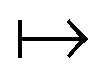
*Γ* employs the Hohenberg–Kohn theorem. It is its simplicity and compactness in the BBGKY sense and also its definite connection with a real world *via* its exactness that make DFT such a fertile ground for the present wealth of DFAs.

This great success of DFT can be favourably compared with a rather tumultuous development of “higher-order” full 1RDM or density-matrix-functional (DMFT) and 2RDM theories, which still do not enjoy a truly successful “take-off”. The ongoing development (see contribution (4.1.1)) explores a way^64^ in which DFT can help DMFT with such a “take-off”, while DMFT can help DFAs with the problematic inclusion in the latter of nondynamical or strong electron correlation.

### 2.2 What is Kohn–Sham DFT?

#### 2.2.1 Perdew

Often we need to predict the ground-state total energy and electron density of a system of real interacting electrons in a scalar external potential (created, for example, by their attraction to nuclei). Correlated wave-function theory provides “the right answer for the right reason”, but at a high computational price for systems of many electrons. Kohn–Sham DFT^65^ employs a simpler noninteracting or Coulomb-uncorrelated wave function, but includes a density functional for the exchange–correlation energy that is exact in principle but requires improvable approximations in practice. It often provides “almost the right answer for almost the right reason at almost the right price” for real atoms, molecules, and materials. The noninteracting kinetic energy and the electron density are found by the not-so-expensive self-consistent solution of effective one-electron Schrödinger equations. Indeed, the exchange–correlation energy and exchange–correlation potential “exactify” the Hartree approximation for the ground-state energy and density. The generalization from total density to spin density^66^ provides more information and enhances the accuracy of the approximations.

#### 2.2.2 Gould

Kohn–Sham DFT^65^ typically means any DFT approximation that employs a set of one-body orbitals, usually denoted {*ϕ*_*i*_}, to produce a kinetic energy functional, *T*_s_[*ρ*] := *T*_s_[{*ϕ*_*i*_}] that approximates the many-body kinetic energy, *T*[*Ψ*] = 〈*Ψ*|*T*|*Ψ*〉. Generalized Kohn–Sham DFT incorporates traditional approaches to DFT as well as “hybrid” functionals, which allow for a nonlocal operator treatment of the Hartree–Fock exchange terms.^67^

As a result, one can replace a many-body interacting Hamiltonian, *H*, by a simpler-to-evaluate one-body Kohn–Sham effective Hamiltonian:8
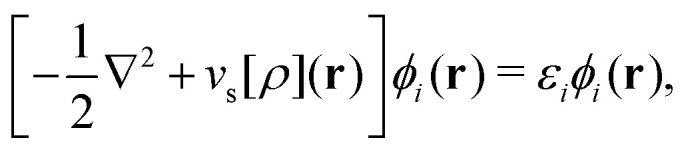
where *v*_s_ is an effective one-body potential (or operator potential). The density may then be calculated as 
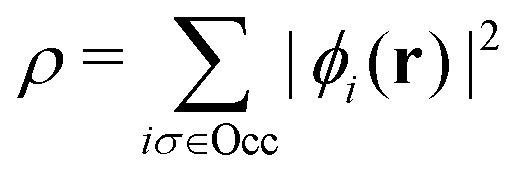
, while the energy is given by *E*_0_[*ρ*] = *T*_s_[*ρ*] + *E*_Hxc_[*ρ*] + (*v*|*ρ*). We will define *v*_s_ and *E*_Hxc_ below.

Formally, one may define 
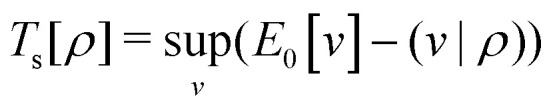
, where 
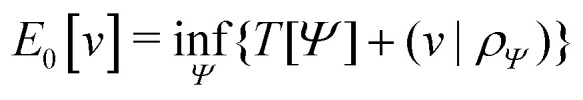
 in the notation defined in contribution (2.1.13).‡‡*T*_s_[*ρ*] has a slightly different meaning in hybrid DFT, where the Slater determinant *Φ* in *T*_s_ = 〈*Φ*|*T*|*Φ*〉 minimizes *T* + *αW* with 0 < *α* < 1 rather than *T*; see Garrick *et al.*^68^ Thus, *T*_s_[*ρ*] is the lowest kinetic energy of a noninteracting system with density *ρ*. Kohn–Sham DFT is useful because the Hartree–exchange–correlation (Hxc) energy,

9 *E*_Hxc_[*ρ*] := *F*[*ρ*] − *T*_s_[*ρ*],

is easier to approximate than *F*[*ρ*]. Here, *E*_Hxc_ incorporates the energetics of the interacting system, including some kinetic-energy terms. The one-body effective potential that minimizes *E*_0_[*ρ*] can be shown to be *v*_s_ = *v* + δ*E*_Hxc_/δ*ρ*.

#### 2.2.3 Gritsenko

A profound physical meaning of the exchange–correlation part of the Kohn–Sham potential *v*_xc_ is revealed with its partitioning

10 *v*_xc_ = *v̄*^hole^_xc_ + *v̄*_resp_

into the potential of the exchange–correlation hole *v̄*^hole^_xc_ and the response potential *v̄*_resp_. This partitioning emerges from differentiation with respect to *ρ* of the exchange–correlation energy *E*_xc_[*ρ*] represented *via* the exchange–correlation pair-correlation function *ḡ*_xc_,11
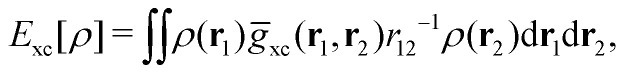
where the overbar indicates the coupling strength integrated pair-correlation function. The potential *v̄*^hole^_xc_, the derivative of the *ρ* functions under the integral, represents the universal interaction (for both occupied and virtual Kohn–Sham orbitals) with the exchange–correlation hole of the unit charge. In turn, the potential *v̄*_resp_, the derivative of the pair-correlation function *ḡ*_xc_, exhibits the spatial step-like structure, with the individual steps distinguishing various atomic and molecular electron shells.^69^

#### 2.2.4 Baerends

The Kohn–Sham method is often cited as the method that made DFT a feasible computational method by offering a decent approximation to (a large part of) the kinetic energy. The latter proved too hard to obtain as a density functional. But more importantly, the Kohn–Sham method has provided DFT with an orbital model. This has greatly facilitated its acceptance in the computational chemistry community. After initial reservations about the Kohn–Sham orbitals (“they are only there to build the density”), it has become evident that these orbitals are not inferior to or more approximate than the Hartree–Fock orbitals, but on the contrary are even more suitable for the qualitative and semiquantitative molecular-orbital (MO) theories of chemistry. If the exact Kohn–Sham orbitals and orbital energies could be obtained, this would be evident. The Kohn–Sham orbitals build the exact electron density, *i.e.*, the exact charge distribution in molecules, so they are perfect for the so-called charge control factor of chemical reactions. The energies of the exact upper valence Kohn–Sham orbitals approximate the first ionization energies exceedingly well: whereas the Hartree–Fock orbital energies, within the frozen orbital approximation for ionization energies (Koopmans’ theorem), deviate typically by more than 1 eV from ionization energies, the exact Kohn–Sham orbital energies have deviations that are typically an order of magnitude smaller.^70,71^ The virtual orbitals of the Kohn–Sham model are not Koopmans-type approximations to the electron affinities, but the virtual-occupied orbital energy gaps are excellent approximations to excitation energies.^72,73^ These are properties that have been the basis for the whole edifice of orbital-based explanations in chemistry.

Ultimately, virtually all explanations of chemical behaviour are cast in orbital language, even if the underlying computations are based on the most sophisticated techniques of theoretical chemistry. The ready acceptance of DFT in chemistry has been greatly aided by the availability of the familiar orbital model. As for the old adage that Kohn–Sham orbitals and orbital energies “have no meaning, there is no Koopmans’ theorem like in Hartree–Fock theory”: the opposite is true.^70,71^

The orbital energies of almost all DFAs do not have the nice properties of the exact Kohn–Sham model, being some 5 eV too high (not negative enough). This is unfortunate and has some adverse consequences, but fortunately the upshift is approximately the same for the upper valence and the lower virtual (valence) orbitals, so the correct relative order is preserved in most DFAs. Nevertheless, more efforts should be made to construct DFAs that obey these exact Kohn–Sham properties (much) more closely.

#### 2.2.5 Krylov

The orbital picture of Kohn–Sham DFT is indeed of great importance. With the exact functional, the energies of the highest occupied Kohn–Sham orbitals become exact ionization energies (IEs) (as per Janak's theorem). Numerical investigations show that the shapes of the Kohn–Sham orbitals in cases when their IEs are close to the exact IE (such as when tuning the range-separation parameter to make the Koopmans IE match Δ SCF IE) become similar to the shapes of the Dyson orbitals.^74,75^ Interestingly, the energies of lower-lying Kohn–Sham orbitals provide surprisingly accurate approximations to the exact many-body IEs (when used with appropriate DFAs),^76,77^ which can be understood by analysing the curvature of the total Kohn–Sham energy with respect to the occupation numbers.^76^

This endows the theory with the ability to provide physically relevant quantities – for example, Dyson orbitals enter the expressions for photoionization/photodetachment cross-sections and can even be reconstructed from experimental data.^74^ Moreover, the orbitals provide a link between many-body theories and DFT – for example, one can judge the quality of a particular DFA by how well the shapes and energies of the Kohn–Sham orbitals agree with those from high-level many-body calculations (*e.g.*, equation-of-motion coupled-cluster theory).^78^ These ideas are already exploited in optimally-tuned range-separated DFAs.^76,77^ But, perhaps more opportunities exist for using *ab initio* Dyson orbitals to build better DFAs?

#### 2.2.6 Calaminici and Köster

To further underline the importance of Kohn–Sham orbitals in chemistry and physics, we mention their interpretative use in cluster science for the definition of so-called superatoms – see, for example, ref. 79 and references therein.

Specifically, the electronic states of small metal clusters are bunched in shells. These shells are experimentally observed in the variations of polarizabilities, ionization energies, and electron affinities – to name a few characteristic observables. Kohn–Sham orbitals, as approximations to Dyson orbitals, reflect these shell structures in a large variety of free and ligand-stabilized clusters. Thus, the now common concept of superatoms in chemistry is based almost exclusively on Kohn–Sham calculations and the corresponding canonical Kohn–Sham orbitals.

#### 2.2.7 Gritsenko

True, the shape of the accurate Dyson orbital of a primary ionization is very close to that of the corresponding accurate occupied Kohn–Sham orbital *ϕ*_*i*_ obtained by “reverse engineering” from the correlated density. However, the same is true also for Dyson orbitals of the satellites of this ionization, reflecting the fact that Dyson orbitals are neither orthogonal to one another other nor normalized. This “unfortunate” feature of Dyson orbitals definitely hinders their comparison with other, “normally behaving” sets of orbitals.

Due to this, the Kohn–Sham orbital energies *ε*_*i*_ differ, in general, from the ionization energies *I*_*i*_ by the spectroscopic average of the satellite ionizations (see contribution (2.4.9)) as well as by the contributions from the response potential (see contribution (2.2.3)), with equality only for the highest occupied Kohn–Sham molecular orbital.^71^ The “well-behaved” (*i.e.*, orthonormal) Kohn–Sham orbitals are, in no way, the “poor cousins” of the Dyson orbitals, forming a distinctively different set of “optimal” orbitals. Indeed, unlike the Dyson orbitals, the occupied Kohn–Sham orbitals meaningfully accommodate the “electron pairs” of conceptual chemistry, while their energies provide a fair estimate of the potentials of primary ionizations. Furthermore, combined with the virtual Kohn–Sham orbitals and their energies, they form the basis for the successful treatment of electronic excitations in TDDFT (see contribution (2.4.9)).

#### 2.2.8 Staroverov

The classic Kohn–Sham scheme almost solves the problem of the kinetic-energy functional but its one-determinantal form creates formidable challenges for approximating the exchange–correlation part. These include the difficulty of devising exchange–correlation functionals for strongly correlated systems (see contribution (3.4.1)), limitations imposed by the assumption of noninteracting *v*-representability by a single Slater determinant, and the intricate behaviour of exact Kohn–Sham potentials (*e.g.*, shifts within nodal surfaces of the highest-occupied Kohn–Sham orbital^80^), which DFAs somehow have to get just right. Although the existing Kohn–Sham DFAs are amazingly more accurate than the Hartree–Fock method in general, it is sobering that they still inherit most qualitative failures (see Section 3.4) of the mean-field approximation. Ensemble methods (see Section 3.7) seem unavoidable from this perspective.

#### 2.2.9 Reining

Just to emphasize a few points, more from a solid-state physicist's point of view: first, Kohn–Sham theory seems to be a natural next step when choosing to work with DFT. Certainly, formulating things (or at least, energies) in terms of functionals of the density is very much helped by the fact that the huge Hartree electrostatic energy is known as an explicit functional of the density. It allows us to have a large part that we know exactly and only a small remainder that has to be approximated.

What is more logical than continuing along this line and taking out another part (the kinetic energy of some noninteracting system)? And what is more logical than taking this noninteracting system to be “similar” to the real system – with the same density, in the spirit of DFT? Generalized Kohn–Sham theory is then also very natural, both because we know more pieces and because (like the kinetic energy) we do not know them as explicit density functionals. Making these pieces and the resulting “potentials” more and more complex appears to build a continuous bridge between Kohn–Sham and Green's functions equations. Another generalization is to start with the consideration that the calculation of any observable will in general integrate out certain details of a system, so the same value for the observable might well be found in a simpler system. This holds for the density – with the Kohn–Sham system, for example – but one can also build auxiliary systems for other observables and profit from the Kohn–Sham experience.

Second, further to the discussion about the Kohn–Sham system, we should keep in mind that, for a single electron, the Kohn–Sham excitation energies equal the exact ones, while the Kohn–Sham electron addition energies are different from the exact ones. So we may expect that, for certain systems, there is a reasonable correspondence for the excitation energies. It is far from obvious that this would also hold true in extended systems with many electrons, and, of course, the Kohn–Sham gap does not equal the optical gap in general. The Kohn–Sham band structure is nevertheless a powerful starting point for calculations using, for example, one- and two-body Green's functions.

Third, the sometimes bad reputation of the Kohn–Sham noninteracting system stems from the fact that it is often used in place of the real system – not to yield simply its density, but also any other observable, in particular, spectral functions. Of course, this can lead to strong disagreement with the truth – and the band gap is just one example. Maybe we should just be more precise in saying what we are doing here – namely, that we use the Kohn–Sham expression (which is a functional of the density) for a given observable as an approximate functional because we do not know a better one? This doesn’t change the results, but it sounds a little more fair to the Kohn–Sham noninteracting system.

#### 2.2.10 Draxl

Indeed, the bad reputation of the Kohn–Sham system may often come from the fact that we either tend to overinterpret results or are not precise enough about what we are doing. Sloppy phrases like “DFT is well known for its notorious band-gap problem” might have been considered appropriate long time ago, but should not be said anymore in 2022. Pointing out the SIE of many functionals is certainly important, but we should always make clear at the very same time that Kohn–Sham eigenvalues are not supposed to provide band gaps.

#### 2.2.11 Baerends

I would like to endorse the statement in contribution (2.2.10) that Kohn–Sham eigenvalues are not (should not be) supposed to provide band gaps. The fact that in solids the Kohn–Sham band gap is not equal to (or close to) the fundamental gap *I*–*A* is extremely frequently cited as the (notorious, infamous,…) band-gap problem. But it is a problem of wrong expectations.

In molecules, it is well known that the Kohn–Sham HOMO–LUMO gap is much below the *I*–*A* difference. This is due to the fundamental difference that the Kohn–Sham system has an attractive potential due to the exchange–correlation hole of −1 electron also for the virtual levels, while the Hartree–Fock system lacks this attractive hole potential for the virtual levels. In the same way, the presence of this *v*^hole^_xc_ potential lowers the LUMO level (bottom of the conduction band, BCB) in solids strongly.^81^ The exchange–correlation hole in solids is pretty localized – at a given point **r**, its size is usually well within a unit-cell range around **r** and therefore its potential is strongly stabilizing. In a delocalized excitation, from an occupied Bloch state to an empty Bloch state, the excited electron does not benefit from this stabilization. Neither does an added electron – the excitation energy to this delocalized state is understandably close to the fundamental gap. So, physically we cannot expect the Kohn–Sham band gap to match approximately the fundamental gap or a delocalized excitation energy. Excitons in a solid (except for Frenkel excitons) typically have a large size, extending over many unit cells. They have excitation energies not much lower than the delocalized excitations, so also for them the attractive Kohn–Sham potential *v*^hole^_xc_ does not fit reality.^81^

The situation is different in molecules since there the physical hole that the excited electron leaves behind is roughly mimicked by the attractive exchange–correlation hole in the Kohn–Sham potential. Hence the Kohn–Sham virtual–occupied orbital energy differences have the nice property that they do approximate excitation energies in molecules;^72,73^ see contribution (2.4.9).

The difference between the Kohn–Sham band gap and the fundamental gap can be cast in the form of expectation values of the response potential part *v*^resp^ of the Kohn–Sham potential;^82^ see also contribution (3.8.6).

#### 2.2.12 Vignale

A question that keeps resurfacing is: Why are the Kohn–Sham orbitals better than the Hartree–Fock orbitals? From the point of view of the variational principle, the Hartree–Fock orbitals should be the best, since they build a Slater determinant which has the lowest energy (defined as expectation value of the Hamiltonian) among all Slater determinants. The Kohn–Sham wave function – also a single Slater determinant – cannot beat that. Nevertheless, we know that the DFT energy is better than the Hartree–Fock energy and also that the Kohn–Sham orbitals, as discussed in contribution (2.2.4), far from being meaningless, are in many ways “better” than the Hartree–Fock orbitals.

The resolution of the apparent paradox is that the Kohn–Sham energy is not calculated as the expectation value of the Hamiltonian in the Kohn–Sham wave function. The moment we adopt the Kohn–Sham approach, the original Hamiltonian of the system is no longer relevant. We are dealing with a reference system that is no longer interacting, but the rules for calculating the energy from the orbitals have also changed and are now expressed in terms of the exchange–correlation energy functional of the density. One could argue that the “particles” of this reference system are the “quasiparticles” of the original system, and this may help to rationalize the *a priori* surprising success of the Kohn–Sham orbitals in predicting single-particle excitation energies.

#### 2.2.13 Baerends

So in what sense are Kohn–Sham orbitals better than Hartree–Fock orbitals? When the energy of the determinant of Kohn–Sham orbitals is calculated with the full Hamiltonian, its energy is of course higher than the Hartree–Fock energy, but actually by only a tiny amount.^83^ On the other hand, the Kohn–Sham density, being equal to the exact one and not so diffuse as the Hartree–Fock one (in molecules), leads to much improved (more negative) electron–nuclear energy. Also the orbital shapes are “better” than the too diffuse Hartree–Fock orbitals (in molecules), so the kinetic energy is also considerably better (higher). The errors of the Hartree–Fock model for these two energy terms are large, in molecules often larger than the bond energy, and they rapidly increase upon bond lengthening.^83^ They cancel to some extent, which is why they are not so readily recognized. The Hartree term is of course also better (exact) with Kohn–Sham orbitals and density.

A tongue in cheek observation would be that the Hartree–Fock model manages to build a determinant that has a little bit lower expectation value of the Hamiltonian, but it has to distort the orbitals (make them more diffuse) because the lowering of the kinetic energy then just outweighs the energy penalty of the increase in the electron–nuclear energy. The Hartree–Fock model does not care – it just tries to get the lowest energy determinant. As noted in contribution (2.2.12), the true power of Kohn–Sham DFT has to come from accurate approximations of the exchange–correlation energy (defined in the Kohn–Sham context), but the good properties of the Kohn–Sham orbitals are an asset of this model.

#### 2.2.14 Gidopoulos

To address the recurring question by Vignale in contribution (2.2.12), I would like to point out that the Kohn–Sham orbitals are in fact as “energetically optimal” as the Hartree–Fock ones. Let me first quote Walter Kohn, who said in his Nobel Prize lecture that, while the Hartree–Fock orbitals are “total energy optimal”, the Kohn–Sham orbitals are “density optimal” because they yield the exact density.

Although, undeniably the Hartree–Fock Slater determinant has the lowest energy among all Slater determinants, we now know that the Kohn–Sham determinant can at least match, if not beat that (record), since it is “energy optimal” in a similar sense: in the Hartree–Fock optimization, we use the full interacting *N*-electron Hamiltonian, *H* and then seek the lowest energy Slater determinant as the best approximate ground state. For the Kohn–Sham orbitals, we may perform an equivalent, but reverse Rayleigh–Ritz optimization: let us assume that the ground state *Ψ* of the physical *N*-electron, interacting system is somehow known (and fixed). Then, we consider all *N*-electron effective, noninteracting Hamiltonians, *H*_*v*_, with a local potential *v*(**r**). The ground-state wave function and energy of each *H*_*v*_ are *Φ*_*v*_ and *E*_*v*_, respectively.

For *N* > 1, *Ψ* cannot be the exact ground state of any of these noninteracting Hamiltonians, *Ψ* ≠ *Φ*_*v*_ for each *v*, because *Ψ* is an interacting state while all *Φ*_*v*_ are noninteracting states (Slater determinants). Hence, the following Rayleigh–Ritz energy difference on the left-hand side is strictly positive:

12 〈*Ψ*|*H*_*v*_|*Ψ*〉 − *E*_*v*_ > 0.

This energy difference gives a measure of how well *Ψ* approximates the ground state *Φ*_*v*_ of the effective Hamiltonian *H*_*v*_. The smaller the energy difference, the better the approximation of *Ψ* to *Φ*_*v*_. It is elementary to confirm that the energy difference is minimized by the exact Kohn–Sham Hamiltonian *H*_*v*_s__.^84^ Interestingly, the exact density property of the Kohn–Sham state is the result of the Rayleigh–Ritz optimization and the density is not *a priori* fixed. Hence, the Kohn–Sham Slater determinant, on top of being “density optimal”, it is also “energetically optimal” in a Rayleigh–Ritz optimization, which physically is equivalent to the total energy minimization of Hartree–Fock theory.

I note that the variational principle in eqn (12) can be used to construct optimally converging power series expansions for the Kohn–Sham potential, without using the adiabatic connection (AC) path formalism.^85^

#### 2.2.15 Yang

I would like to address the physical meaning of Kohn–Sham orbitals in calculations with DFAs. Most DFAs to the exchange–correlation energy *E*_xc_ usually produce reasonable total energies for small and medium-size molecules; however, they have major deficiencies in the orbital energies. As has been known for a long time, for finite systems, the eigenvalue of the HOMO for the exact Kohn–Sham potential is equal to the negative of the first ionization potential (IP), as follows from the asymptotic decay behaviour of the exact electron density and the requirement that the Kohn–Sham effective potential be zero at infinity.^86^ However, in a Kohn–Sham calculation, the local Kohn–Sham potential can have any additive constant and give the same total energy and density but different orbital energies. Thus, the argument based on the long-range behaviour of density and potential hinges on a particular choice of the additive constant of the potential.

The orbital energies for the frontier HOMO and LUMO were rigorously shown to be the DFA prediction of the negative of the first IP and the first electron affinity (EA) in 2008.^21^ Three key results were used in the proof. (1) The Janak theorem shows that Kohn–Sham orbital energies are the derivatives of the total energy with respect to the orbital occupation numbers. Note that the Janak theorem does not relate orbital energies to any physical observables.^87^ (2) The left and right derivatives of the total energy with respect to the total electron number, or the left and right chemical potentials, are the negative of the first IP and the first EA, respectively, of the corresponding energy functional. This follows from the linear condition on the behaviour of the total energy for fractional number of electrons.^18^ The linearity condition is true for the exact functional, or for a functional without delocalization error for general systems. For infinite bulk systems, however, the linearity condition holds true for any functional approximation.^16^ (3) The chemical potentials were proved to be the derivatives of the energy with respect to the orbital occupation numbers of HOMO and LUMO in a Kohn–Sham calculation, when the exchange–correlation energy used is a functional of the density. With the use of the Janak theorem, this then establishes that the Kohn–Sham HOMO and LUMO energies are the chemical potentials of the system for the given DFA.^21^ Similarly, when the exchange–correlation energy is a functional of the noninteracting one-electron density matrix, the chemical potentials were proved to be the derivatives of the energy with respect to the orbital occupation numbers of HOMO and LUMO in a generalized Kohn–Sham calculation.^21^ Therefore, the HOMO and LUMO orbital energies are the DFA prediction of the negative of the first IP and the first EA. This interpretation of the HOMO and LUMO energies holds true for molecular and bulk systems, for any given DFA.

Indeed, DFAs with minimal delocalization error^23–25^ have excellent predictions of IPs and EAs from the HOMO and LUMO of generalized Kohn–Sham calculations, comparable to the accuracy of *GW* approaches.^88^ In addition, the orbital energies above the LUMO and below the HOMO approximate the corresponding quasi-particle energies, with similar accuracy as the HOMO/LUMO for the IP/EA. This has been explored to describe accurately the excitation energies and conical intersections of molecular systems in the quasi-energy DFT approach based on ground-state generalized Kohn–Sham calculations.^88,89^

#### 2.2.16 Baerends

In relation to contributions (2.2.15) and (2.2.11), it should be stressed that it is very important to distinguish between the properties of, on the one hand, the exact (original) Kohn–Sham model of noninteracting electrons in a local potential such that the exact density is reproduced and, on the other hand, the currently popular DFAs – in particular, those of the generalized Kohn–Sham family with nonlocal potentials. The local Kohn–Sham potential is unique by application of the Hohenberg–Kohn theorem to the noninteracting electron system, and so are the orbitals and orbital energies.

The attractive properties of the exact Kohn–Sham orbitals and orbital energies have been expounded in some contributions; see contributions (2.4.9), (2.2.4), (2.2.13), and (2.2.11). A salient feature of the exact Kohn–Sham model is that the LUMO is not at −*A* (given that the HOMO is at −*I*) but much lower: the HOMO–LUMO gap is approximately equal to the first excitation energy.^72,73,90^ It should be made clear that contribution (2.2.15) does not contradict these properties of the exact Kohn–Sham model. It refers to a different family of Kohn–Sham models, usually called the generalized Kohn–Sham models. These generalized models make it possible to include, for instance, part of the exchange operator (a nonlocal potential) of the Hartree–Fock model and adjust the local part of the potential so that the density remains exact and adjust the exchange–correlation functional so that the energy also remains exact.^67^ In such a scheme, the orbital energies are different from those generated by the exact local Kohn–Sham potential. In such a generalized Kohn–Sham model, one may strive to obtain that the HOMO is again at −*I* and that the LUMO is now at −*A*, as is also done in the Koopmans-compliant functionals.^91,92^ The LUMO then becomes more diffuse and one loses the simple representation of excitations in TDDFT with just one or a few orbital transitions.^73^

#### 2.2.17 Yang

In relation to the discussion in contributions (2.2.15) and (2.2.11) on the physical meaning of the HOMO and LUMO in DFT, it is important to separate the two types of one-electron Kohn–Sham Hamiltonians. The first one is from the ground-state calculation with a given DFA *E*^DFA^_xc_, which yields the density, orbitals and orbital energies of the noninteracting reference system, as developed in the original Kohn–Sham paper.^65^ This is called the direct approach.^93^ The second one is from an inverse calculation, generating the local potential *v*^*σ*^_s_(**r**) that reproduces a given ground-state electron density, which can be the exact density or an accurate density from high-level calculations. We called this the inverse Kohn–Sham or inverse OEP approach,^93^ the potential so obtained is also called the “exact Kohn–Sham” potential by Baerends in contribution (2.2.16).

In an inverse approach, the local potential is determined up to an arbitrary constant. Thus, in principle, the absolute values of the orbital energies are not defined. However, if the correct asymptotic condition on the potential is satisfied, which also sets the constant, then *ε*_H_ = −*I* is obtained, where *I* is the experimental ionization energy, if an exact density is given (row 1 Table 1). Similarly, a good approximation to the experimental −*I* is expected if a good approximation to the density is given from a DFA calculation (row 1 in Table 1). However, the corresponding LUMO energy has not been shown to relate to the ionization energy and is not a good approximation to the experimental −*I*, as discussed in contribution (2.2.16). In atomic calculations, the unoccupied-orbital energies, {*ε*_a_}, obtained from inverse Kohn–Sham calculations, have been shown to represent electronic excitations, with *ε*_a_ − *ε*_H_ describing excitation energies of the system with the same number of electrons. Using *ε*_a_ − *ε*_H_ to approximate excitation energies for molecules is less successful.

**Table 1:** Properties of the electron density *ρ*^*σ*^_s_(**r**) and HOMO and LUMO orbital energies, *ε*_H_ and *ε*_L_, of the noninteracting reference systems in exact DFT (*E*^DFT^) and various DFA models. The DFA models include all continuous functionals of the density *E*^DFA^_xc_[*ρ*^*σ*^_s_(**r**)], continuous functionals of the non-interacting density matrix *E*^DFA^_xc_[*ρ*^*σ*^_s_(**r**′,**r**)], and continuous functionals of the noninteracting orbitals and the external potential *E*^DFA^_xc_[{*ϕ*_*pσ*_},*v*_ext_(**r**)]. Computational approaches for *ρ*^*σ*^_s_(**r**), *ε*_H_ and *ε*_L_ include inverse calculations from a given (accurate) electron density and direct calculation methods based on the original Kohn–Sham approach (KS), the optimized effective potential (OEP), the generalized Kohn–Sham (GKS) and the generalized optimized effective potential (GOEP, which has been shown to be equivalent to orbital optimization (OO); see Jin *et al.*^94^). Three properties of each quantity are considered for each computational approach: (1) agreement of *ρ*^*σ*^_s_(**r**), the density of the noninteracting reference system, with *ρ*^*σ*^(**r**), the density of the physical system consistent with the exact DFT, or the density of the DFA as defined by the linear response 
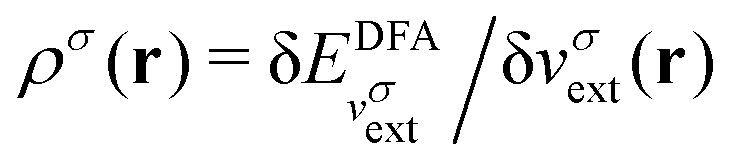
; see Chen *et al.*,^95^ Voora *et al.*;^96^ (2) agreement of the HOMO orbital energy *ε*_H_ with 
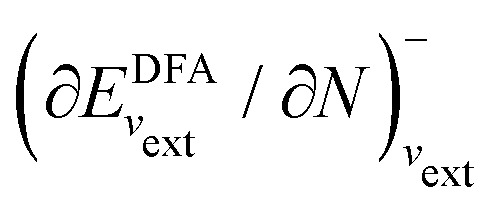
 the chemical potential of electron removal for the functional employed; (3) agreement of the LUMO orbital energy *ε*_L_ with 
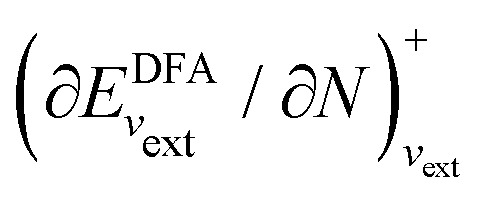
 the chemical potential of electron addition for the functional employed. No entry indicates that it is impossible or not yet known how to conduct the corresponding calculation. (Table provided by Yang, extended from ref. 93)

Noninteracting system	Type		*E* ^DFT^	*E* ^DFA^_xc_[*ρ*^*σ*^_s_(**r**)]	*E* ^DFA^_xc_[*ρ*^*σ*^_s_(**r**′,**r**)]	*E* ^DFA^_xc_[{*ϕ*_*pσ*_}, *v*_ext_(**r**)]
Inverse KS/inverse OEP *v*^*σ*^_s_(**r**)	Inverse	*ρ* ^*σ*^_s_(**r**)	Yes	Yes	Yes	Yes
		*ε*_H_	a	b	b	b
		*ε*_L_	No	No	No	No
KS *v*^*σ*^_s_(**r**)	Direct	*ρ* ^*σ*^_s_(**r**)		Yes		
		*ε*_H_		Yes		
		*ε*_L_		Yes		
OEP *v*^*σ*^_s_(**r**)	Direct	*ρ* ^*σ*^_s_(**r**)		Yes	Yes/no^c^	No
		*ε*_H_		Yes	d	
		*ε*_L_		Yes	No	
GKS *v*^*σ*^_s_(**r**,**r**′)	Direct	*ρ* ^*σ*^_s_(**r**)		Yes	Yes	
		*ε*_H_		Yes	Yes	
		*ε*_L_		Yes	Yes	
GOEP/OO *v*^*σ*^_s_(**r**,**r**′)	Direct	*ρ* ^*σ*^_s_(**r**)		Yes	Yes	No
		*ε*_H_		Yes^e^	Yes^e^	f
		*ε*_L_		Yes^e^	Yes^e^	f

a In an inverse calculation, the potential is determined up to an arbitrary constant and the absolute values of the orbital energies are therefore undefined. However, if the correct asymptotic condition on the potential is imposed, which also sets the constant, then *ε*_H_ = −*I*, is obtained, where *I* is the experimental ionization energy.^86^

b If the correct asymptotic condition on the potential is imposed, and if a good electron density is obtained from the DFA, then the inverse OEP calculation will leads to *ε*_H_ that is a good approximation to the experimental −*I*.

c The agreement between *ρ*^*σ*^_s_(**r**) with 
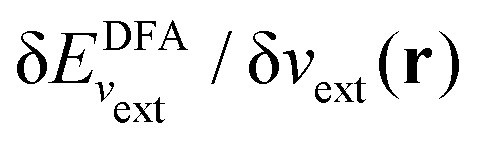
 is only true at the complete basis set limit for the basis set expansion of *v*^*σ*^_s_(**r**), and not so for any finite basis set.^93^

d Similar to (b), if the correct asymptotic condition on the potential is imposed, then the direct OEP calculation will lead to *ε*_H_ that is a good approximation to the experimental −*I*.

e For explicit functionals of the density, or the density matrix, GOEP/OO gives the same total energies and density matrix as in regular SCF. But the orbitals obtained in general are no longer the canonical orbitals and thus have no orbital energies directly. However, a unitary rotation can bring them to the canonical orbitals with proper orbital energies in agreement with the corresponding chemical potentials.

f In GOEP or OO calculations, the Hamiltonian for the noninteracting system is not available, so neither are the noninteracting orbital energies.

In a direct calculation with a DFA – that is, when the energy is minimized with respect to its variables, as discussed in contribution (2.2.15) – the HOMO energy of the noninteracting reference system has been shown to be equal to the chemical potential for electron removal13
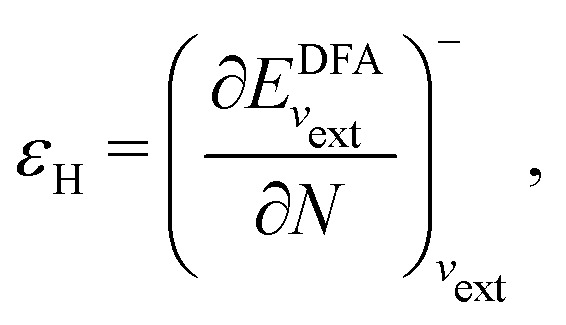
and LUMO energy of the noninteracting reference system has been established as being equal to the chemical potential for electron addition14
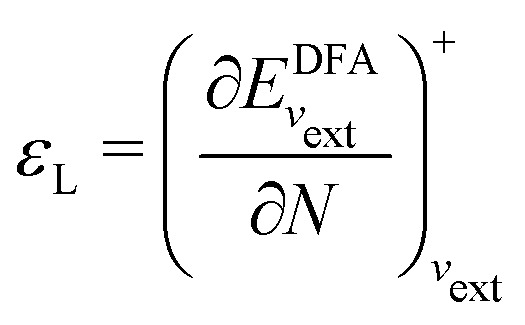
for a Kohn–Sham calculation with *E*^DFA^_xc_[*ρ*^*σ*^_s_(**r**)] and also for a generalized Kohn–Sham calculation with *E*^DFA^_xc_[*ρ*^*σ*^_s_(**r**′,**r**)] in the work of Cohen, Moris-Sanchez and Yang^21^ (rows 2 and 4 in Table 1). Note that these identifications are based on the assumption that *E*^DFA^_xc_[*ρ*^*σ*^_s_(**r**)] and *E*^DFA^_xc_[*ρ*^*σ*^_s_(**r**′,**r**)] have an explicit and continuous dependence on its variables *ρ*^*σ*^_s_(**r**) and *ρ*^*σ*^_s_(**r**′,**r**). But no locality is assumed. With these identifications, the use of HOMO/LUMO energy to approximate −*I*/−*A* was then established,^21^ building on the PPLB condition for fractional number of electrons and its results for chemical potentials.^18^ The quality of the approximation of *ε*_H_ to −*I* and/or *ε*_L_ to −*A* just reflects the quality of the DFAs used, where the delocalization error of the DFA plays a key role;^16^ see contribution (2.2.15).

There are other approaches to direct calculation, using as the basic computational variable either a local potential *v*^*σ*^_s_(**r**) in an OEP approach or a nonlocal potential *v*^*σ*^_s_(**r**,**r**′) in the direct generalized OEP (GOEP) approach.^94^ The meaning of HOMO and LUMO energies in direct OEP calculations was established in ref. 21; see also Row 3 in Table 1.

In Table 1, we also list the results on the agreement of the electron density of the noninteracting reference system with the density of the physical system as defined by the linear response.^93^

#### 2.2.18 Trickey

The pervasive emphasis on the Kohn–Sham orbitals to this point in the discussion is striking and, from the perspective of my interest in orbital-free DFT, a bit overbalanced. From that perspective, the Kohn–Sham orbitals and eigenvalues are not the crucial insight provided by the Kohn–Sham decomposition – that crucial insight is the existence (assuming *v*-representable densities) of a noninteracting system with the same density as the many-body system. With that assumption, existence is provable by application of Levy^6^–Lieb^8^ for the ground-state and Runge–Gross^97^ (as updated by Ruggenthaler *et al.*^98,99^) for the time-dependent case and Mermin^50^ for the temperature-dependent case. The orbitals (and eigenvalues) are a valuable, exploitable by-product.

Particularizing to the ground state, Kohn–Sham DFT is, at base, the decomposition of the Levy–Lieb functional (putting aside to a separate discussion the issues associated with the original Hohenberg–Kohn and later Levy–Lieb functionals) into physically recognizable, interpretable, and computable parts. Orbital-free DFT (better called one-orbital DFT) exploits only the decomposition, while conventional Kohn–Sham DFT also uses the Kohn–Sham orbitals explicitly. Both variants (to use a currently prominent word) are fundamentally Kohn–Sham theory. Both have the same definitions of kinetic energy, Hartree energy, exchange energy, and correlation energy. All those definitions depend upon the Kohn–Sham determinant.

The distinction between those two variants is operational – namely, what is done to exploit the Kohn–Sham decomposition computationally. This is crucial because of the many statements that one sees to the effect that orbital-free DFT is an “alternative formulation of DFT” that avoids the problems of Kohn–Sham theory, *etc.* That completely ignores the underlying Kohn–Sham logic. That logic is in fact crucial to constraints on approximate kinetic-energy density functionals (KEDFs).

#### 2.2.19 Gritsenko

The unique feature of the exchange–correlation part of the local Kohn–Sham potential is the richness of the physical information on the local effects of electron correlation, as reflected in the shape of the potential. This can be favourably compared with the nonlocal Hartree–Fock and self-energy potentials of the wave-function theory produced from the corresponding kernels. The shape of the latter potentials is “ruined” with singularities related to the orbital nodes. Contrary to this, the steps of the Kohn–Sham exchange–correlation potential meaningfully distinguish the local correlation effects in adjacent atomic and molecular shells with the corresponding “gauges” (see contribution (2.2.3)), while its integer discontinuity “jumps” signal occupation of (previously virtual) Kohn–Sham orbitals.

Then, instead of complaining about “the idiosyncratic behaviour” of the Kohn–Sham exchange–correlation potential, one should fruitfully explore and employ this meaningful information – see, for example, contributions (3.1.12) and (3.8.6). Moreover, one should not attempt to “wash away” this precious true information by constructing artificially too smooth Kohn–Sham exchange–correlation potentials by “reverse engineering” techniques.

As to the generalized Kohn–Sham scheme, the term ‘Kohn–Sham’ seems to be misused in this case. Indeed, out of desire to get electron affinities as the energies of virtual orbitals, the original Kohn–Sham theory is forcefully “crashed” in some (out of infinitely many) variants of the generalized Kohn–Sham “landscape” by mixing different theories both globally and with range-separation techniques.

#### 2.2.20 Görling and Kronik

With respect to the term ‘generalized Kohn–Sham’, we feel that it is appropriate. The generalized Kohn–Sham approach^67^ relies on the basic idea of the original Kohn–Sham formalism by exploiting the Hohenberg–Kohn theorem to introduce a model system with the same ground-state density, in order to have access to quantities that help in the description of the electronic system. Such quantities can be energies, typically the ‘noninteracting’ kinetic energy or the exchange energy, but can also be orbital eigenvalues. The generalized Kohn–Sham approach generalizes the Kohn–Sham one in the sense that it extends the range of possible model systems. Like all proper generalizations, it contains the original Kohn–Sham approach as a special case. As also discussed in contribution (2.4.8), the generalized Kohn–Sham approach provides more flexibility and establishes a firm formal foundation for frequently used methods that do not calculate the exchange–correlation potential as a functional derivative with respect to the electron density, notably hybrid functional methods. And, as also discussed in contribution (4.1.5), a specific generalized-Kohn–Sham map need not be “crashed”, but rather can be judiciously chosen, nonempirically, based on physical constraints.

#### 2.2.21 Görling

It is instructive to define which electronic-structure approaches are Kohn–Sham methods. Such a definition reveals the key characteristics of the Kohn–Sham formalism and shows the scope and perspective that the Kohn–Sham formalism provides. By a quite wide definition, those methods are Kohn–Sham methods that rely on a model system of noninteracting ‘electrons’ with the same ground-state electron density as the true physical electronic system and with a local multiplicative potential. The noninteracting ‘electrons’ are particles that are identical to electrons – in particular, they have the spin of electrons – but do not interact among themselves. Given that the particles of the Kohn–Sham system are noninteracting, the Kohn–Sham equation for the Kohn–Sham orbitals and their eigenvalues in eqn (8) emerges immediately.

Traditionally, the Kohn–Sham orbitals are used only to evaluate the kinetic energy of the Kohn–Sham model system, which represents the bulk of the full kinetic energy, taking into account the fermionic nature of electrons. The Kohn–Sham orbitals, however, contain much more information than their kinetic energy. The occupied Kohn–Sham orbitals, for example, enable an exact calculation of the exchange energy. This means that all parts of the total energy except the correlation energy can be easily calculated exactly, technically by evaluating the Hartree–Fock energy with Kohn–Sham orbitals. Indeed, approximating only the remaining small part of the energy, the correlation energy, is a natural and systematic approach. For individually approximating the correlation energy, orbital-dependent functionals^40^ can be constructed that use occupied as well as unoccupied Kohn–Sham orbitals and their orbital energies, in this way exploiting much more of the information contained in the Kohn–Sham model system.

Historically, this route was not pursued for three reasons:

(1) to avoid the high cost of evaluating the exact exchange energy, which nowadays is not really a problem for molecules up to a size of several hundred atoms. For larger systems or when very many electronic-structure calculations are required, in *ab initio* dynamics simulations, for example, the cost of exact exchange remains an issue.

(2) to benefit from error cancellation between exchange and correlation contributions. While this is a valid reason, the cancellation is not complete, limiting the accuracy that can be reached by traditional Kohn–Sham methods.

(3) to avoid the problem that the exchange potential is not directly accessible in terms of the Kohn–Sham orbitals. With the OEP method, functional derivatives of orbital-dependent energy expressions, including – for example, the Kohn–Sham exchange potential – are accessible.^40–46^

While basis-set OEP methods were numerically problematic in the past, robust, numerically stable basis-set OEP methods are now available.^46^ Moreover, orbital-dependent functionals can be evaluated in a post-self-consistent-field (post-SCF) manner, avoiding the need to take functional derivatives of orbital-dependent functionals with respect to the electron density. Alternatively, functional derivatives can be taken with respect to orbitals instead of the electron density, leading to generalized Kohn–Sham methods.

Meta-GGA and hybrid functionals are established functionals that depend on the occupied orbitals. Correlation functionals based on the adiabatic connection fluctuation-dissipation (ACFD) theorem^100,101^ depend on unoccupied as well as occupied orbitals and their eigenvalues. The simplest example of such a functional is the correlation energy within the random-phase approximation (RPA).^102–104^ All these methods are Kohn–Sham methods or, depending on the way the exchange–correlation potential is obtained, generalized Kohn–Sham methods.

#### 2.2.22 Trickey

The remark by Görling about the computational cost of exact exchange deserves emphasis. He observes that the cost “nowadays is not really a problem for molecules up to a size of several hundred atoms. For larger systems or when very many electronic-structure calculations are required, in *ab initio* dynamics simulations, for example, the cost of exact exchange remains an issue.”

This is a crucial distinction between gas-phase chemistry and materials physics and chemistry. For those with access to significant computing resources, exact exchange is not prohibitive for the comparatively small number of calculations needed to study an isolated molecule of up to a few hundred atoms. But that is manifestly not true for *ab initio* molecular dynamics (AIMD) of several thousand molecular-dynamics (MD) steps used to screen tens of different but kindred condensed-phase systems, for each of which the constituents are molecules with 300 or more non-hydrogen atoms. This distinction illustrates the compelling importance of continued effort to improve lower-rung DFAs. It also is but one example that there is more than gas-phase chemistry at stake in the development of DFT methodology and algorithms.

#### 2.2.23 Jones

I agree with Trickey in contribution (2.2.22) and Görling in contribution (2.2.21). The computational effort required in many “real-world” applications is often underestimated – see also Trickey in contribution (3.2.12), concerning other problems of extended systems. A single MD simulation of nanoseconds with a time step of femtoseconds can mean millions (!) of self-consistent DFT calculations of a system with hundreds of atoms.^105^ A factor of ten (or even of two) in computer time per time step can mean the difference between completing the calculation and abandoning it.

#### 2.2.24 Savin

The Hohenberg–Kohn theorem is valid for many Hamiltonians, including those with no interaction between particles. The latter case shows already the difficulty of constructing closed-form approximations to an energy density functional. Kohn and Sham decided to alleviate the treatment of electronic systems by treating accurately a (model) noninteracting system and by using density-functional corrections only for the difference between the energy of this system and the system of interest, with interacting electrons. Note that this idea is easily extended to other model Hamiltonians, making it possible to go beyond the use of a single Slater-determinant reference within DFT – see, for example, ref. 106.

#### 2.2.25 Tozer

A feature of regular Kohn–Sham calculations using common exchange–correlation functionals is that the electronic energy does not in general equal the sum of the occupied orbital energies. Recently, Levy and Zahariev^107^ proposed the direct energy Kohn–Sham (DEKS) scheme, whereby the Hartree-exchange–correlation potential is shifted by a constant, in order to make the electronic energy equal to the sum of the orbital energies. This shifted potential has attractive theoretical characteristics and so it is desirable to try to model it directly for use in DEKS calculations. The use of density-scaling homogeneity considerations is one possible way forward.^108^

#### 2.2.26 Arbuznikov

The remarks of Schwerdtfeger in contribution (2.1.31) have prompted me to add a few words on relativistic exchange–correlation functionals.

Despite the lack of a rigorous theory that would allow one to construct them in a systematic way, a potentially useful pragmatic solution within the Dirac–Coulomb–Breit framework has been known for a long time. Since relativistic effects become important at high densities – that is, in exchange-dominated core regions – one could, in a first approximation, restrict oneself to an appropriate treatment of the exchange energy. For the exchange energy of the relativistic homogeneous electron gas (RHEG),^57,59,109^ a multiplicative correction (a kind of “enhancement factor”) has been derived as a simple analytic function *Φ*(*β*), where *β* = (3π^2^*ρ*)^1/3^/*c* (in atomic units). This function satisfies *Φ*(*β*) > 1 and tends to one at the low-density limit; it is a sum of both Coulomb (longitudinal) and Breit (transverse) contributions. This scheme has been implemented and tested for atoms at the LDA level^110^ and subsequently extended to the GGA level^111^*via* data on the linear response of the RHEG to a weak perturbing potential.^57^ Data for several small diatomics are available as well.^112^

While valence-shell related properties turned out not to be sensitive with respect to these corrections,^112^ a high sensitivity of core one-electron energies of heavy atoms has been clearly demonstrated.^111^ For heavy atoms, these corrections seem to be of the same order of magnitude as atomic (nonrelativistic) correlation energies.^110^ So far, it appears that these corrections have not yet been implemented into a molecular or solid-state code. Obviously, studies of the impact on core-related properties will be of interest. Recently, short-range LDA and GGA exchange functionals have been developed and implemented in a similar way,^113,114^ but again only for atoms and ions so far.

A very recent development of a potentially useful relativistic local hybrid functional^115^ within an X2C code should be mentioned as well.

### 2.3 What can be described with DFT?

#### 2.3.1 Helgaker

Pure (non-Kohn–Sham) DFT provides the ground-state density and the ground-state energy. We can then (in principle) obtain rigorously all properties that can be expressed as functions of the density and the energy – for example, derivatives of the energy with respect to nuclear displacements or nuclear magnetic moments (provided DFT has been extended to deal with magnetism as discussed in contribution (4.3.1)). We can in principle also calculate excitation energies, from equiensembles.

In practice, we do Kohn–Sham DFT, which in addition to the density and the ground-state energy (in principle, both exact) also gives us the Kohn–Sham noninteracting wave function, from which many more properties of the system can be obtained, but only approximately, given that the Kohn–Sham wave function is a noninteracting approximation to the exact many-body wave function.

We are of course free to use the Kohn–Sham wave function as a zero-order starting point for a many-body treatment – but we are then leaving the domain of DFT.

#### 2.3.2 Görling

The ground-state electron density yields the electron number and the Hohenberg–Kohn theorem tells us that it determines furthermore the external potential and thus the Hamiltonian operator which determines all properties of an electronic system. Therefore the ground-state electron density determines the energy and the properties of the ground state and of all excited states. In practice, we typically use DFT to get information on ground-state properties and for excited states we switch to TDDFT in the linear response regime. However, it might be worthwhile to devote more effort to explore how excited-state energies and properties can be obtained in DFT without invoking TDDFT.

#### 2.3.3 Krylov

I would like to see some thoughts of how to approach the problem of extracting properties that cannot be formally expressed in terms of the electron density or one-particle density matrix. The value of *S*^2^ is such a property.

#### 2.3.4 Reining

I agree, in principle, that we should get from the density all properties that are determined by the external potential and the number of electrons. Why do we then feel that we have so little diversity in the observables that are traditionally dealt with in DFT? First, this statement is actually not true, if we consider the Kohn–Sham observables as approximations to the true density functional of, for example, spectra – there are many such calculations around. The Kohn–Sham expressions are of course not explicit functionals of the density, but implicit ones, *via* the orbitals. But why is it so difficult to go beyond the Kohn–Sham approximation and find better ones for these observables?

Again, this is actually not completely the case. Take the polarizability – we do go beyond the Kohn–Sham independent particle polarizability, by adding Hartree (*i.e.*, the bare Coulomb interaction in the integral kernel of the Dyson equation) effects in the RPA, and even exchange–correlation effects through the exchange–correlation kernel, which is also a density functional. Like Görling in contribution (2.3.2), you might object that this is TDDFT, but I would say it is linear response in the ground state, so we are talking about functionals of the ground-state density. Simply, we have derived this ground-state density functional using TDDFT, but who cares how we derived it once we have it? We could of course dream of finding simpler functionals for the polarizability, maybe even explicit functionals of the ground-state density, but since even the kinetic energy is so difficult, I wouldn’t bet on this in the near future.

#### 2.3.5 Yang

An exact DFT calculation for the ground state of an *N* electron system provides directly the ground state total energy *E*_v_(*N*) and electron density. It also provides the ground state energies for the corresponding (*N* − 1) and (*N* + 1) electron systems directly through the chemical potentials of the *N* electron system. The extension of a similar connection to the excited states of the corresponding (*N* − 1) and (*N* + 1) electron systems has recently been made.^88,89,116,117^ However, since the exact functional is not available in an explicit form, neither is the method for the associated chemical potential calculations. We now focus on the discussion on explicit functional forms that include most existing DFAs.

For an *N*-electron system, a Kohn–Sham calculation with an exchange–correlation functional that is an explicit and continuous functional of the electron density leads directly to *E*_v_(*N* − 1) and *E*_v_(*N* + 1), the ground-state energies of the corresponding (*N* − 1) and (*N* + 1) electron systems. Similar connections follow for a generalized Kohn–Sham calculation with an exchange–correlation functional that is an explicit and continuous functional of the noninteracting reference density matrix. This is true because of the following: (1) it has been proved that the HOMO/LUMO energy is the chemical potential for electron removal/addition,^21^ (see Table 1) (2) the PPLB condition shows that the chemical potential of the *N*-electron system is −*I* and −*A*.^18^ Thus the band gap can be predicted from the HOMO–LUMO gap, in either Kohn–Sham calculations with an explicit functional of the electron density or generalized Kohn–Sham calculations with an explicit functional of the noninteracting reference density matrix. This connection is independent of the functional approximation. However, the accuracy of the prediction depends on the quality of the functional used.^21^ For functionals with minimal delocalization error, the prediction is comparable to, or better than, that of *GW* approaches.^25,88,89^

Similarly to the access to the ground state information of the corresponding (*N* − 1) and (*N* + 1) electron systems, it has been argued recently that *ε*(*N*), the orbital energies of orbitals above LUMO and below HOMO also approximate the corresponding quasiparticle energies *ω*^+/−^(*N*) as follows: *ε*_*m*_(*N*) ≈ *ω*_*m*_^+^(*N*) = *E*_*m*_(*N* + 1) − *E*_0_(*N*), and *ε*_*n*_(*N*) ≈ *ω*_*n*_^−^(*N*) = *E*_0_(*N*) − *E*_*n*_(*N* − 1). This then links directly to the excited-state energies of the corresponding (*N* + 1) and (*N* − 1) systems.^88,89,116,117^ Extensive numerical evidence supports this claim.^88,89,116,117^ Thus, the excited-state energies of *N* electron systems can be obtained from ground-state calculations on the (*N* − 1) or (*N* + 1) electron systems.^88,89,116,117^

### 2.4 What concepts are useful for the development and understanding of DFT?

#### 2.4.1 Perdew

An open subsystem of fluctuating (and thus on average noninteger) electron number is a surprisingly useful concept. Real atoms have integer electron numbers, but local and semilocal approximations to the DFA for the exchange–correlation energy spuriously predict the transfer (delocalization) of a fraction of an electron between two different well-separated open-shell atoms (or between two open subsystems of a combined system). Nature's integer preference is explained by invoking an ensemble description of each separated open quantum subsystem that is equivalent to making a wave-function description of the combined system.^18^ When the electron number in the open quantum subsystem is varied between two adjacent integers, its exact total energy and electron density vary linearly with the electron number (piecewise linearity), so the exact energy minimizes at an integer electron number.

This has important practical consequences. In particular, local and semilocal approximations predict incorrect energies and densities for a diatomic molecule AB in the dissociation limit. In fact, these approximations are much more accurate for integer than for fractional electron numbers. This problem still plagues density functional approximations. A non-self-consistent cure is to evaluate the approximate functionals on Hartree–Fock densities, which localize an integer charge around each separated nucleus.^118^ Doing that also cures some related problems, such as spurious charge transfers at smaller internuclear separations.

#### 2.4.2 Perdew and Savin

In many cases, the energy and wave function of the interacting system can be connected smoothly to those of the Kohn–Sham noninteracting system of the same electron density. Then the exact exchange–correlation energy for that density becomes an integral over the strength of the electron–electron interaction, which subsumes both the potential energy of exchange and correlation and the kinetic energy of electron correlation. The AC and the idea of modelling the pair density associated with it^100,119–121^ served as the key inspiration not only for passing from LDA to GGAs,^122,123^ but also for making the step to hybrid functionals.^124^ Note that it is not necessary to use the pair density in the adiabatic coupling; one can use the first-order density matrix as well – see, for example, ref. 125.

#### 2.4.3 Sun

Related to the concepts mentioned above – that is, the AC and fractional charges – the concept of the exchange–correlation hole has been useful for the development and understanding of DFT. For example, the sum rules for the exchange and correlation holes have been used to explain the successes of LDA, while the successful PW91 GGA functional was constructed by enforcing the sum rules for the exchange and correlation holes on the gradient expansion approximation of slowly varying densities. The construction of the SCAN meta-GGA was guided by the understanding of the exchange and correlation holes. In particular, prototypical systems with very localized exchange correlation holes can be used as appropriate norms, whose exchange–correlation energies can be exactly or nearly exactly predicted by a semilocal density-functional approximation. Semilocal approximations, whose underlying exchange–correlation-hole models are necessarily semilocal, must fail to describe systems with delocalized exchange correlation holes – for example, systems characterized by fractional charges.

#### 2.4.4 Xu

The AC path mentioned in contribution (2.4.2), which bridges the fictitious noninteracting Kohn–Sham system to the fully interacting real system, is one of the most important concepts in the development and understanding of DFT.^100,101^ The coupling-constant integration along the AC path defines the Kohn–Sham exchange–correlation functional, which also accounts for the kinetic energy of correlation.^125^ The more we know about the AC path, the better DFAs we can construct.

The first widely recognized hybrid DFA is Becke's half-and-half functional.^124^ It was derived based on a linear model for the AC path, which was then empirically extended, leading eventually to the widely used B3LYP functional.^126–129^ More sophisticated AC models have been used to develop and rationalize the popular “nonempirical” PBE0 functional,^130^ as well as some other hybrid functionals.^131^

The AC formalism has provided an important playground for the development of the advanced DFAs that involve the unoccupied Kohn–Sham orbitals. The random-phase approximation (RPA) was introduced to the DFT community *via* the ACFD formalism.^100,132^ Görling–Levy (GL) perturbation theory^133^ shows that the initial slope of the AC path is twice the second-order GL perturbation energy (GL2). For systems with a linear AC path, the exact exchange–correlation functional is therefore nothing but the exact exchange plus GL2 correlation energy.^134^ The AC formalism has motivated the initial developments of several successful double-hybrid approximations by further mixing the second-order perturbation (PT2) energy with the already successful hybrid functionals.^134–137^

#### 2.4.5 Gori-Giorgi

The AC can be mathematically extended outside the usual range between the Kohn–Sham and the physical systems – for example, to negative coupling strengths (attractive electrons)^138^ or, more interestingly, to very large positive coupling strengths (electrons repelling each other infinitely strongly, or, equivalently, the Levy–Lieb functional in the *ℏ* → 0 limit^139,140^). This latter case defines the limit of strictly correlated electrons (SCE),^141–144^ which yields the functional complementary to the Kohn–Sham kinetic energy – that is, the minimum possible electron–electron interaction of a system with given one-electron density *ρ*(**r**); see eqn (76) in contribution (4.5.8). The SCE functional also yields the exact low-density limit of the exchange–correlation functional of Kohn–Sham DFT. Although chemical systems are usually very far from this limiting situation, the SCE functional sheds light on the nonlocal nature of the exact exchange–correlation functional and can inspire the construction of new approximations to handle strong correlation.^145–147^

Another way to use the SCE limit in chemistry is to build interpolation models of the AC between the Kohn–Sham limit (which may include exact exchange and second-order perturbation theory) and the expansion at strong coupling strength.^142,148–151^ The interpolation strategy based on global quantities (integrated over all space, a strategy that can be viewed as creating nonlinear hybrids and double hybrids) was abandoned for some time because of its lack of size consistency. However, more recently, it has been shown that size consistency can be easily restored for these functionals at no extra computational cost.^150^

#### 2.4.6 Teale and Helgaker

The AC is certainly a powerful tool for understanding the universal density functional. Using the Lieb variation principle (see contribution (2.1.13)), the AC can be calculated to high accuracy using many-body wave-function techniques.^152–155^ As well as the usual linear AC path, generalized AC paths, such as those based on the error function, can be calculated and are relevant for range-separated hybrid functionals.^11,156,157^

Such calculations can also be used to extract the coupling-constant-dependent one- and two-particle density matrices. The one-particle density matrices may be used to define an AC focusing on the kinetic component of the DFT correlation energy – see, for example, ref. 125 and 158, as alluded to in contribution (2.4.8) and calculated in ref. 159. The two-particle density matrices can be used to give direct access to the exchange–correlation hole and its coupling-constant average.^149,160^ All these quantities can be determined using high-level *ab initio* methods, giving valuable insight into the near exact behaviour of *F*[*ρ*]. The challenge is to parameterize simple models to construct useful DFAs – work that is still an active area of research.

All of the AC pathways mentioned above focus on the density-fixed case, relevant to Kohn–Sham DFT. However, if one notes the conjugate relationship between *F*[*ρ*] and *E*[*v*], a natural alternative is a potential-fixed AC, a possibility that has also been explored numerically.^154,161^ Since the density is no longer fixed, the calculations of the AC pathway are in the potential-fixed case much simpler to perform, but the noninteracting reference system (the bare nucleus system) is farther from a realistic electronic system than its Kohn–Sham counterpart. Recently, other ACs have been developed that do not insist on a fixed density along the AC pathway – see, for example, ref. 162 for an AC that recovers the Møller–Plesset series as its low coupling-strength expansion. The utility of the AC as a concept for understanding new theories based on these alternative pathways and their relative pros and cons compared with the Kohn–Sham approach underlines its importance as a concept in electronic-structure method development.

#### 2.4.7 Kaupp and Arbuznikov

The AC, which has already been invoked in contributions (2.4.2)–(2.4.6) as an important principle for the development of DFAs, is usually applied to the energy functional, where its existence is well established.^163,164^

Increasingly, however, interpolations along local ACs have been used, meaning that the coupling-strength (*λ*) integration is applied to the corresponding energy density or even to the exchange–correlation hole followed by integration over one and two spatial coordinates, respectively. While the existence of a “local AC” has never been proven rigorously, Becke argued that such an approach does not violate any basic principles and is just a matter of changing the order of integration that is valid for continuous functions^165^ – see also, for example, ref. 149.

One of the first applications of the local AC to the development of DFAs was to derive the B88 correlation functional^166^ Given that the global AC is the founding principle underpinning (global) hybrid functionals (see contributions (2.4.2), (2.4.4) and (2.4.6) above),^124,128,167^ a local AC should be relevant for local hybrid functionals (LHs) with position-dependent exact-exchange admixture. Let us mention in passing our early attempts to derive local mixing functions for LHs from local AC interpolation.^168^ Other important hyper-GGA functionals simulate strong correlation effects and also make use of local interpolations.^165,169^

Most notable in this context are recent efforts to include the SCE limit (*λ* → ∞) of the AC by local AC interpolation.^149,170–172^ Importantly, the local AC approach has advantages compared to the global AC in terms of achieving size-consistency for DFAs in the presence of degeneracies.^173^

#### 2.4.8 Kronik

An important concept that I have found to be very useful is that of generalized Kohn–Sham theory, introduced by Seidl *et al.*^67^ This involves mapping of the many-electron system onto a partially interacting model system, represented by a single Slater determinant, such that the ground-state electron density is conserved. The original Kohn–Sham theory then emerges as a special case of generalized Kohn–Sham theory, where the partial interaction is set to zero.

Generalized Kohn–Sham theory, recently extended to both TDDFT^174^ and ensemble DFT,^175^ provides a useful viewpoint that rigorously justifies the use of nonmultiplicative potentials. In particular, this means that the use of Fock-exchange potential operators (and variants thereof) in hybrid functionals (both global and range-separated), originally viewed as an *ad hoc* and theoretically unjustified merger of Kohn–Sham and Hartree–Fock theories, are rigorously derived and justified within generalized Kohn–Sham theory.^176^ While, for a given system, there is only one exact Kohn–Sham map, there are infinitely many partially-interacting systems to which an exact generalized Kohn–Sham map may be formed.^68^ This added flexibility has been found to be useful for spectroscopy – in particular, for choosing generalized-Kohn–Sham maps in which the derivative discontinuity is eliminated; see elaboration in contribution (4.1.5).

#### 2.4.9 Gritsenko

A useful concept of Kohn–Sham DFT is the meaning of the energies of the Kohn–Sham orbitals. According to the Kohn–Sham analogue of Koopmans’ theorem,^70^ the energy *ε*_*i*_ of the occupied Kohn–Sham orbital *ϕ*_*i*_ can be interpreted as approximate relaxed vertical potential *I*_*i*_ of the primary ionization, *ε*_*i*_ ≈ −*I*_*i*_. The quality of this approximation is better for the outer-valence Kohn–Sham orbitals, with equality for the HOMO. The deviation of *ε*_*i*_ of the lower-lying Kohn–Sham orbitals from −*I*_*i*_ is, primarily, due to the spectroscopically-averaged contributions from ionization of the corresponding “shake-up” satellites.^71^ The energies *ε*_*a*_ of virtual Kohn–Sham orbitals *ϕ*_*a*_ include the “excitonic” type particle–hole interaction; see contribution (2.2.3). The difference *ε*_*a*_ − *ε*_*i*_ therefore serves as a good-quality zero-order estimate of the corresponding excitation energy from TDDFT. With the electron affinity provided by the energy of the anionic Kohn–Sham HOMO, the Kohn–Sham orbitals deliver all the important one-electron quantities.

#### 2.4.10 Baerends

The formal theory – beginning with the Hohenberg–Kohn theorem – is clear enough. It offers the prospect of finding important properties, notably the energy, as functionals of the electron density. However, the functional relation between the density and the energy remains obscure. The theory tells us that the density uniquely determines the energy (or rather, that each ground-state density is associated with a specific energy), but it tells us nothing about the precise relation. When two densities are close to each other (given some topological definition of distance between densities), there is no guarantee that the corresponding energies are also close. In other words, we do not (yet) know how to derive from the one-electron density information on the pair density, which determines the (correlation) energy. It is a fundamental weakness of the theory that it provides no clue to the solution of this problem.

In *ab initio* quantum chemistry, the route that is followed, in many different ways, consists of finding computationally feasible and sufficiently accurate approximations to the full configuration-interaction (FCI) solution. One may call this a mathematically-oriented approach. One can view DFT as an attempt – maybe often unconsciously – to follow the route of finding physical models for the pair-correlation function. The largest part of the LDA functional is the exchange functional, which is practically the same as Slater's original *ρ*^4/3^ approximation. Slater derived his “exchange hole” from a simple model (local hole of constant depth integrating to −1) which leads to practically the same exchange energy expression (with *ρ*^4/3^ density dependence) as the homogeneous electron gas of LDA. But the Slater construction shows that the interpretation is not necessarily that of an electron-gas exchange approximation. Indeed, it has been realized that this simple local hole is much more like an exchange–correlation hole – for instance, accounting for considerable left–right correlation in the chemical bond (which is why it soon was called an exchange–correlation approximation).^69^ The Slater (or LDA) hole yields much better bond energies for prototypical diatomic molecules than the exchange hole of the Hartree–Fock model: bonding changes from severe underbinding in Hartree–Fock theory to some overbinding in LDA. Also, the systematic errors in frequencies and bond distances that characterize the Hartree–Fock model disappear. This tells us that modelling of the exchange–correlation energy may not be so difficult after all.

The major improvement in the step from LDA to GGA comes from improved modelling of the exchange approximation. Becke's parameterization was fitted to reproduce the exchange energies of the rare-gas atoms, and Perdew's nonempirical GGA approximation of the exchange hole likewise considerably improved atomic exchange energies. The main contribution to the success of GGA (improvement over LDA) for bonds in simple diatomic molecules originates from these exchange improvements. This is mystifying since one would expect the better exchange approximation of GGA to reproduce more closely the poor Hartree–Fock results. Apparently, the GGA exchange improvements have turned the exchange holes into better exchange–correlation holes. So, there is still a considerable lack of precise understanding why the most successful models work, which perhaps explains the lack of consistent improvement beyond the GGA level. On the other hand, the “physical route” to the correlation problem by modelling of the exchange–correlation hole is hopefully a fruitful way forward that can be pursued independently of the clarification of the mathematical intricacies of DFT.

#### 2.4.11 Gori-Giorgi

Besides the argument that the exchange–correlation hole is much more localized than the exchange hole,^69^ other possible ways to understand the interesting point made in contribution (2.4.10) about fitting exchange on atoms and getting correlation in molecules could be:

(i) the exchange energy functional (and the exchange hole) changes linearly under uniform coordinate scaling. The correlation energy (and hole), by contrast, does not exhibit any simple scaling. However, when the electron–electron interaction becomes dominant with respect to the kinetic energy (see contributions (2.4.5) and (4.5.8)), then the exchange–correlation energy scales again as the exchange energy.^170,177,178^ It might thus make sense to have an exchange-like functional to capture (at least part of the) static (left–right) correlation.

(ii) More recent work by Burke, Perdew and coworkers (see ref. 179 for a recent review) has clarified the sense in which LDA is a universal limit for coulombically bound systems, with exchange as the leading term.

#### 2.4.12 Krylov

Reduced quantities, such as state and transition density matrices, natural orbitals, natural transition orbitals (NTOs), and Dyson orbitals, are very useful for understanding what DFT can and cannot do.^74^ These objects are also useful for making rigorous connections between DFT and wave-function theories, as well as for interpretation.

For example, NTOs afford a unified and rigorous description of electronic transitions in terms of MO theory, which is also experimentally verifiable, noting that observables such as absorption cross-sections can be rigorously expressed in terms of matrix elements between hole and particle NTOs. By using natural orbitals and their occupations, one can compare such properties as diradical character and the number of effectively unpaired electrons;^180^ although not observable, these quantities are useful for understanding the underlying electronic structure and for judging whether DFT captures the physics of the problem.

#### 2.4.13 Krylov

The observation that the response of the density of one electronic state (*e.g.*, the ground state) contains the information about the entire spectrum of the system is both an opportunity for useful extensions and a liability in the context of the applicability of the theory. This observation has been used to extend Kohn–Sham DFT to describe excited states *via* TDDFT.

The same observation, coupled with the fact that the quality of the response of the density depends on the quality of the density, also enabled the extension of Kohn–Sham DFT to electronic structures that have multiconfigurational character and, therefore, are not well described by the single determinant. Although with exact Kohn–Sham DFT, we should be able to treat any type of electronic structure, current DFAs implicitly rely on the expectation that a single Slater determinant provides a good zero-order representation of the wave function; consequently, most Kohn–Sham DFAs fail when the electronic structure is multiconfigurational.

In the spin-flip DFT (SF-TDDFT) approach, the “difficult” multiconfigurational states (such as diradicals, molecules with broken bonds, transition metals) are described by means of spin-flipping excitations from a well-behaved high-spin reference state.^181–183^ In exact DFT, this approach should yield identical results to the traditional Kohn–Sham treatment – however, with the current incarnations of Kohn–Sham DFT, the SF-TDDFT method provides an effective solution to certain types of multiconfigurational states.

Thinking in terms of response properties also helps to understand when to anticipate potential problems. For example, while a TDDFT calculation may yield excellent excitation energies for a few valence states of interest, it may fail miserably in describing nonlinear properties, such as two-photon cross-sections, if the chosen functional does not treat (higher-lying) Rydberg states correctly.

### 2.5 What useful concepts of electronic structure theory have emerged from DFT?

#### 2.5.1 Gill

Inspection of the electronic Schrödinger equation for a large molecule does not lead one to anticipate that most of its interesting properties can be partitioned into almost-additive contributions from its various parts. As a result, there is an apparent inconsistency between the baroque complexity of quantum chemistry's many-body framework and the simplicity and predictive power of chemistry's “functional group” paradigm. Kohn–Sham DFT models, in which the exchange–correlation energy is approximated by an integral over all space of a function of the electron density, have partially bridged that conceptual gap.

#### 2.5.2 Kronik

Regarding contribution (2.5.1): interestingly, decades after his seminal DFT work, Walter Kohn formalized the idea of almost-additive local contributions by introducing the concept of “nearsightedness” of electrons in many-atom systems; see also contribution (5.4.7). Nearsightedness means that (with some caveats), for a fixed chemical potential, local electronic properties, such as the density, *ρ*(**r**), depend significantly on the effective external potential only at nearby points.^184,185^

#### 2.5.3 Ayers, Chattaraj, Chermette, De Proft, Fuentealba, Geerlings, Liu, Vela, and Yang

In the variational equation of DFT, the Lagrange multiplier *μ* was identified by Parr and coworkers in 1978^47,186^ as the partial derivative of the energy *E* with respect to the number of electrons *N*, at constant external potential *v*(**r**),15
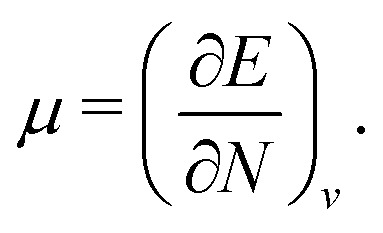
The chemical potential is the negative electronegativity *χ* = −*μ* by the Iczkowski–Margrave definition^187^ and reduces to the Mulliken electronegativity in a finite-difference approximation. As the electron density *ρ*(**r**) can be shown to be equal to the functional derivative of the energy with respect to the external potential,16
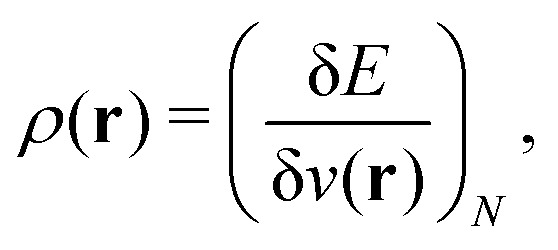
two basic quantities *μ* and *ρ*(**r**) can thereby be seen as responses of the energy to perturbations in *N* and *v*, respectively.

This observation forms the basis of conceptual DFT where, starting from the energy functional *E*[*N*,*v*] for atoms, molecules, and the solid state, derivatives of the type 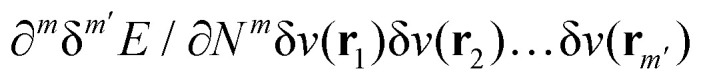 are identified as response functions of the system's energy to perturbations in *N* and *v*, important for chemical reactions, with *μ* and *ρ* being the first-order (*n* = *m* + *m*′ = 1) responses. Second-order properties (*n* = 2) like the chemical hardness,17
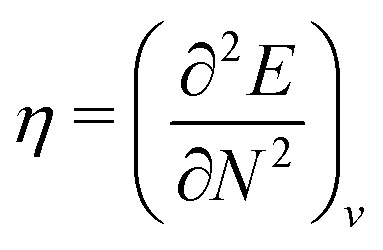
and its inverse, the chemical softness *S* = 1/*η*, the Fukui function18
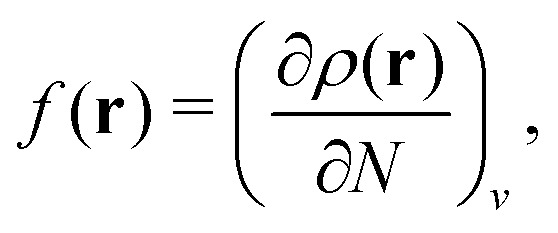
the linear response function19
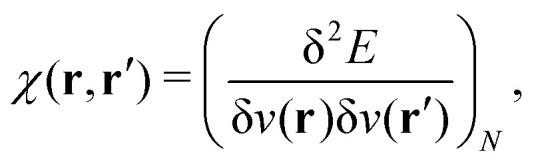
and even third order properties, with20
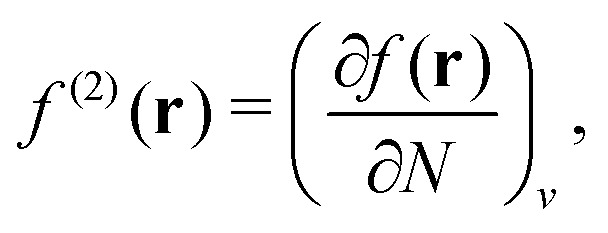
as the most representative member the dual descriptor, followed. All of them have proven their merits as concepts in the electronic-structure theory of atoms, molecules and the solid state,^188,189^ emerging in a natural way in conceptual DFT which, on the basis of the initial identifications, forms an integral part of DFT.

All these response functions and some others derived from the energy function *E*(*N*), of which the electrophilicity *ω* is the most eminent representative,^190,191^ were shown to bear chemical relevance. To give some examples: the chemical hardness *η* in eqn (17) was identified with Pearson's hardness, while the Fukui function *f* (**r**) was recognized as a generalization of Fukui's frontier MO concept, its product with the total softness, the local softness *s*(**r**) = *Sf*(**r**) being a local indicator for soft regions in a molecule.

The first conceptual development of the chemical potential *μ* was based on the assumption that the fundamental functional *E*[*N*,*v*(**r**)] is differentiable everywhere for both variables.^186^ Subsequently, the exact piecewise linear conditions at fractional particle numbers were established originally by Perdew, Parr, Levy, and Baldus (PPLB)^18^ based on grand canonical ensembles at zero temperature and later by Yang, Zhang, and Ayers based on pure states with degeneracy.^20^

The piecewise linearity of *E*[*N*,*v*(**r**)] with respect to *N* means that the derivatives at integer electron numbers are discontinuous. In particular, at a given integer *N*, the chemical potential *μ* = (∂*E*/∂*N*)*v*, the Fukui function *f* (**r**) = (∂*ρ*(**r**)/∂*N*))*v* and other related quantities are discontinuous, the corresponding left and right derivatives being different. In view of this discontinuity, use of the derivative notation is best understood with an underlying finite-difference mathematical definition,^47,192^ where *e.g.* the right derivative is obtained by evaluating *E*(*N* + 1) − *E*(*N*). This interpretation is particularly important for second derivatives such as the chemical hardness in eqn (17).^47^ Since the chemical hardness describes the change in the first derivative at an integer electron number *N*, it will be zero or infinite at *N* and have no physical meaning unless interpreted in the above finite-difference manner. The PPLB condition is thus the foundation for the discussion of derivatives. It leads to the identification of the left and right chemical potentials with the ionization energy *I* and the electron affinity *A*, respectively.^18^ This identification was used to establish the physical meaning of the HOMO and LUMO orbital energies as the density-functional prediction of *−I* and *−A*, respectively, associated with the functional approximation used.^21^

#### 2.5.4 Liu

The use of density functionals to quantify and rationalize traditional chemical concepts and physiochemical properties is an ongoing research topic in DFT.^193^ The first example was by Nalewajski and Parr,^194^ who proved that the Hirshfeld partitioning (the Hirshfeld charge) arises from the constrained minimization of information gain (the Kullback–Leibler divergence, an explicit density functional), subject to the normalization condition of the total electron density.

Steric effects have been quantified in DFT by the Weizsäcker kinetic energy functional.^195^ Its functional derivative has been employed to predict stereoselectivity.^196^ Pauli energy has been validated as a robust identifier for double, triple, quadruple, and even higher covalent bonds.^197^

#### 2.5.5 Staroverov

The electron localization function (ELF)^198,199^ and related tools for analysing the nature of chemical bonds^200^ come from DFT and are now ubiquitous in computational chemistry. Other examples include the average local ionization energy^201–203^ and classical turning surfaces of atoms and molecules.^204^

#### 2.5.6 Pernal

The concept of the AC, conceived within the DFT framework and successfully used to develop approximations to exchange–correlation functionals – see contributions (2.4.2)–(2.4.7) – has inspired the development of methods for calculating the dynamical correlation energy in wave-function theories.^205^ In the general AC theory developed in ref. 205 and elsewhere, one is not restricted to adopting a noninteracting Kohn–Sham system as a reference system, corresponding to a vanishing coupling constant. If, instead, the reference wave function consists of a combination of Slater determinants and orbitals are partitioned into noninteracting groups (most commonly into doubly occupied (inactive), fractionally occupied (active), and unoccupied (virtual) orbitals, as in multiconfigurational self-consistent-field (MCSCF) theory) then, by following the AC path, the limit of no correlation is smoothly connected with the full electron-correlation limit.

A difference between AC-DFT and multiconfigurational AC theory is that, in the former theory, the electron density is fixed to the exact density by a local one-body potential varying along the AC path, while in the latter, the condition of a constant density is imposed as an approximation. AC-based correlation energy approximations have been used with MCSCF, complete-active-space SCF (CASSCF), density-matrix-renormalization group (DMRG) and geminal theories.^205,206^ These multiconfigurational AC approximations rely on the ACFD formalism and the (extended) RPA. An appealing feature of the multiconfigurational AC methods is that only one- and two-electron reduced density matrices are needed, as opposed to perturbation approaches such as complete-active-space second-order perturbation theory (CASPT2) or *N*-electron valence-state second-order perturbation theory (NEVPT2), which require three- and four-body RDMs.

It has been recognized that, in the general AC theory, the reference state need not be an electronic ground state as long as it is not degenerate. This has motivated the development of AC methods for excited states, which recover the dynamical correlation energy for a specific state.^207^ It may be worth exploring if a similar approach could be developed for Kohn–Sham DFT, taking an excited Kohn–Sham determinant as the noninteracting system. The clear advantage over TDDFT would then be the description of states of double-excitation character.

#### 2.5.7 Pernal

There has always been an intuitive understanding that short-range correlation relates to the electron cusp in the wave-function description, while long-range correlation plays a role when electron pairs dissociate or when van der Waals bonds are formed. Range separation of electron correlation has gained mathematical rigour in the range-separated multiconfigurational formulation of DFT.^208,209^ In range-separate multiconfiguational DFT (RS MC-DFT), only the long-range part of the electron interaction operator, which is bounded at electron coalescence and is characterized by a proper Coulomb tail, is retained in the many-body Hamiltonian. Consequently, a wave function in RS MC-DFT has no electron cusp, which greatly simplifies the many-body problem – approximate wave functions call for shorter configuration-interaction (CI) expansions than when the full Coulomb interaction operator is used.

The long-range electron correlation energy naturally emerges as the difference between the energies of the FCI wave function and the chosen model (CI, CASSCF, *etc.*).^210,211^ The short-range correlation energy is rigorously defined as21
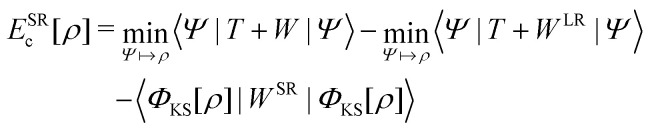
and depend on the underlying range-separation of the electron interaction operator, *W* = *W*^LR^ + *W*^SR^. Approximations for the short- and long-range correlation energies can be developed independently. One of the appealing features of RS MC-DFT is that wave-function models and existing approximate exchange–correlation functionals may be adapted to a range-separated electron interaction.

The rigorous range separation of electron correlation has led to a proliferation of wave-function methods using short-range exchange–correlation functionals as an inexpensive way of accounting for dynamical (short-range) correlation, thereby improving their accuracy and/or efficiency.^212^ A promising direction of development of DFT *via* its merger with wave-function theory is enabled by gaining access to a correlated two-particle local function – the on-top pair density – which can be used as a variable in correlation functionals in addition to the electron density; see, for example, ref. 213 and contribution (4.1.1).

#### 2.5.8 Grimme

In the early days of quantum-chemical method development for electronic structure, drastically simplified methods for large systems termed “semiempirical” like MNDO, AM1 or PM6^214^ were derived as approximations to Hartree–Fock theory.^214^ Usually, minimal atom-centred atomic-orbital (AO) basis sets and severe integral (multipole) approximations were applied, enabling a reasonably accurate, extremely fast treatment of mostly organic molecules. Because of the applied zero-differential-overlap (ZDO) approximation and their Hartree–Fock origin, these methods are not robust for more complicated electronic systems like, for example, the important class of organometallic catalysts.

This situation changed in the mid 1990s when the tight-binding (TB) semiempirical theory was proposed as an approximation to Kohn–Sham DFT,^215,216^ based on previous work of Foulkes and Haydock.^217^ The current theoretical view on TB methods, which in the meantime have been consistently parameterized for the whole periodic table,^218^ is based on a Taylor expansion of the total energy *E* around a reference density *ρ*_0_, constructed as a sum of atomic valence densities:

22 *E*[*ρ*] = *E*^(0)^[*ρ*_0_] + *E*^(1)^[*ρ*_0_,δ*ρ*] + *E*^(2)^[*ρ*_0_,(δ*ρ*)^2^] + *E*^(3)^[*ρ*_0_,(δ*ρ*)^3^] + …

where the fluctuations δ*ρ* are expressed in terms of multipoles and the series is usually truncated at third order. Short-range repulsive, exchange–correlation as well as dispersion effects are typically described using empirical pairwise potentials.

The speed-up of a TB calculation compared to, for example, a regular GGA(PBE) DFT calculation, is about three orders of magnitude, at little loss of accuracy for common properties like electronic and geometric structures. Thermochemical data and, in particular, conformational energies are generally not so well described, which is at least partially attributed to the small (mostly minimal) AO basis sets employed.^218^ The development of more accurate, but still sufficiently fast, TB methods is an important future field that should take advantage of more advanced DFAs.

#### 2.5.9 Aradi and Frauenheim

The efficient DFT-based TB methods are not restricted to “classical” DFT and to ground-state properties only. Several DFT extensions have been successfully ported into the density-functional tight-binding (DFTB) framework^215^ and implemented in various program packages. The DFTB version of those extensions (hybrid functionals^219^ TDDFT,^220^ Ehrenfest dynamics,^221^ Green's-function-based electron transport,^222^*etc.*) are typically several orders of magnitude faster than their DFT counterparts, allowing for a more efficient treatment of large systems and/or long time scales.

#### 2.5.10 Köster

For decades, X_*α*_ and Kohn–Sham DFT methods have served as a playground for the development of density-fitting methods.^223,224^ Commonly used approaches are the variational fitting of the Coulomb^225^ and Fock^226^ potentials. With these fitting approaches, the formal scaling of first-principles Hartree–Fock and Kohn–Sham calculations is reduced by one order of magnitude without lowering the accuracy of the underlying methodology. To avoid linear-algebra bottlenecks associated with variational density fitting, iterative Krylov subspace solvers are advocated.^227^

A further simplification of Kohn–Sham DFT implementations can be achieved by using the approximate density from the variational fitting of the Coulomb potential for the evaluation of the exchange–correlation energy and potential.^228,229^ The resulting energy expression remains variational and yields optimized structures and relative energies that are almost indistinguishable from standard Kohn–Sham approaches, but at a substantially reduced computational effort. The extension of this auxiliary DFT (ADFT) approach to perturbation theory permits first-principles molecular property calculations of systems with up to thousand atoms – for example, second-order analytic energy derivatives.^230^ Most recently, ADFT also serves as platform for the development of new DFAs.

#### 2.5.11 Galli

DFT in its approximate Kohn–Sham formulation has been key in the description of chemical bonding in condensed systems, including solids and liquids, in different phases and under different thermodynamic conditions. It has been especially critical for understanding trends in chemical bonding in solids as a function of temperature, pressure and, more recently, even external fields, although we are still far from having accurate descriptions in many cases. It is also important to note that the use of approximate DFT (beginning with LDA) is at the basis of the development of first-principles molecular dynamics and hence the ability to study finite-temperature properties of materials.

Orbitals obtained from the solution of the Kohn–Sham equations are also at the basis of most many-body perturbation theories solving, in approximate manners, the Dyson and Bethe–Salpeter equations (*GW* and BSE methods). These approaches have brought tremendous progress in understanding properties of solids, in spite of some lack of accuracy, and almost all of them (for solids) are based on DFT.

#### 2.5.12 Reining

Further to the usefulness of DFT as starting point for Green's functions methods, I would like to point out that combinations, for example, approximations for vertex corrections beyond the *GW* method are derived from DFT and TDDFT.

## 3 Density functional approximations

### 3.1 What strategies have been useful in constructing DFAs?

#### 3.1.1 Chermette

It is worth recalling that DFAs span a wide range, from quasi *ab initio* to fully semiempirical status. The first category, promoted by Perdew and collaborators, introduces parameters that are almost all fixed by theoretical constraints. This approach, which allows us to use the resulting exchange–correlation functionals in exotic systems with some confidence (assuming universality of the functional), may, however, involve constraints that can be questioned, as being not necessarily appropriate for molecular systems – for example, the uniform-gas limit. This constraint was removed by Handy *et al.*^231^ in the OPTX exchange functional and is a reminiscent of the X-alpha functional. This approach, coupled to a correlation functional (*e.g.*, in the OPBE functional), may lead to a good description of spin states.^232^

The second category, promoted by the Minnesota team, has led to functionals involving up to 64 parameters. These functionals may be very accurate for – but limited to – small classes of molecular systems and properties. In a paper involving 200 combinations of exchange and correlation functionals,^233^ Mardirossian and Head-Gordon compared the performance of these exchange–correlation functionals applied to 82 data sets, with and without dispersion corrections, and documented the scattering of the performance among the properties for given classes of molecular systems.

#### 3.1.2 Perdew

The original local density approximation (LDA)^65^ for the exchange–correlation energy was based upon fitting to an appropriate norm or system for which the approximation is exact: the electron gas of uniform density. Nonempirical generalizations of LDA have been constructed by satisfying additional exact constraints or mathematical properties derived from exact but impractical expressions (see, for example, ref. 6, 100 and 120) for the functional. For example, the PBE functional^234^ satisfied 11 exact constraints, and the SCAN functional^235^ was constructed to satisfy all 17 known exact constraints that a meta-GGA can satisfy. The SCAN functional also fits generalized appropriate norms, such as the hydrogen atom and neutral atoms of large atomic number. By contrast, empirical constructions are fitted to experimental or higher-level computational data (usually for molecules), which can make them more reliably interpolative and less widely predictive than the nonempirical functionals.

Of course, these two approaches are often combined. The most accurate functionals (including meta-GGAs, hybrids, and RPA-like functionals) generalize Kohn–Sham theory^67^ by employing as arguments of the energy density not only the electron density and its gradient, but also the occupied or even the unoccupied orbitals or one-electron wave functions, and by optimizing those arguments. A sometimes important but seldom discussed step in the development of a functional is “deconstruction”: removing what is wrong or unnecessary, as in the transition from gradient expansions to generalized gradient expansions.^123^

#### 3.1.3 Chermette

As suggested in contribution (3.1.2), in case of semiempirical functionals (see contribution (3.1.1)) which may involve dozens of parameters, it is especially important to remove all parameters with statistically insignificant weights in the fits. The reason is that these parameters introduce noise in the calculations and restrict severely the application domain to the classes of molecular systems that have been used in the training set. Approaches like variance analysis spring to mind, but more elaborate methods may also be used – for example, Mardirossian and Head-Gordon^236^ have detailed the strategy they used for a combinatorial approach to handle the problem, which is made more complicated by the fact that the objective function to be optimized (usually a least-squares sum) is a (linear) combination of inhomogeneous quantities (energies, structural data, other physical properties) that are combined with *ad hoc* weights in the objective function.

#### 3.1.4 Adamo and Ciofini

The terms “empirical” and “nonempirical” used above deserve some clarification. For us, the term “nonempirical” denotes those DFAs whose internal coefficients are not determined by an error minimization relative to external reference data sets (experimental or theoretical), but instead are fixed using only constraints derived by theory. The term “empirical” denotes, by contrast, those functionals whose coefficients are determined by a parameterization procedure. However, since these latter functionals may also respect some theoretical constraints, we prefer to use the term “semiempirical” to underline their theoretical foundation. In our opinion, these two terms, “nonempirical” and “semiempirical”, are not measures of quality, but rather indicate how the functional has been developed. Between these two classes, the term “minimally parameterized” is also used, to underline that an effort has been made to reduce the number of functional parameters, as mentioned above.

#### 3.1.5 Loos

The uniform electron gas, a hypothetical infinite substance where an infinite number of electrons “bathe” in a (uniform) positively charged jelly of infinite volume, is one of the success stories of DFT and, in particular, the parameterization of its correlation energy as a function of the density has been enormously useful for the construction of DFAs.^237^ From a more general point of view, model systems (especially the ones with uniform electron densities) provide new ways for improving and testing DFAs.^238^ In this regard, finite uniform electron gases (where electrons are confined to the surface of a sphere) can be seen as an extension of the conventional “infinite” version thanks to additional degrees of freedom coming from the tunable “finiteness” of the electron gas.^239,240^

#### 3.1.6 Reining

I would like to elaborate on contribution (3.1.5) of Loos: using results from the uniform electron gas has been invaluable for the success of DFT. Here, we should stress how much DFT has profited from other people's work and methods – in particular, from the quantum Monte Carlo calculations of Ceperley and Alder.^241^ This is important: trying to use the strong points of other methods – and trying to use the knowledge of model systems for the real materials we are interested in. This strategy could be extended much further.^242^

#### 3.1.7 Savin

An important decision in constructing DFAs is the choice of parts to be approximated by a closed form. Hohenberg and Kohn already considered it necessary to treat exactly the Hartree term, thus treating the electrostatic contribution to the energy correctly.^5^ Kohn and Sham decided to leave the DFA for exchange and correlation (Section II.A of their paper), or for correlation only (Section II.B).^65^ One can discuss having a part of exchange treated by orbitals and a part by DFAs, as done in hybrid DFT.^124^ One can also decide to treat only a part of correlation by a DFA – see, for example, ref. 243.

#### 3.1.8 Staroverov

The analytic derivations of density functionals for model systems that gave us the Dirac exchange and Thomas–Fermi theory, as well as derivations of DFAs from model exchange–correlation holes^166,244^ have been seminal. The success of this analytic approach seems difficult to sustain in DFT, but that is almost certainly because not everything has been tried. Attempts to connect DFT with wave-function methods explicitly can also result in effective practical methods, especially for calculations of accurate Kohn–Sham potentials.^245–247^

#### 3.1.9 Johnson

Explicit modelling of the separate exchange, dynamical, and nondynamical correlation holes has been a very successful strategy in functional development. The (exact) exchange–correlation energy can be written in terms of the exchange–correlation hole as:23
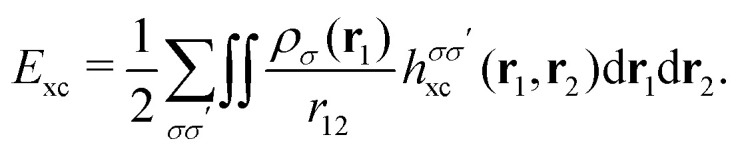
The total exchange–correlation hole can be decomposed into separate exchange, parallel-spin correlation, and opposite-spin correlation holes. Real space models can then be proposed that obey known constraints, such as normalization, as well as density and curvature constraints at a reference point.^244^

Another useful strategy in the development of GGAs is to enforce a large-gradient limit of the enhancement factor,^248,249^ which ensures an accurate treatment of nonbonded repulsion in van der Waals complexes.^250,251^ Such functionals are capable of high accuracy for modelling intermolecular interactions in both gas-phase and solid-state systems, when paired with a density-functional dispersion correction.^252^

#### 3.1.10 Adamo and Ciofini

It is worth underlining how the respect of known theoretical constraints can help in the development of DFA approximations. In this sense, we should first mention Becke's half-and-half model, which introduces the AC at the heart of functional construction.^124^ Another example is the PBE0 functional,^167,253^ defined based on the ansatz of Perdew and co-workers for the form of the AC path.^130^ The relationship between the AC ansatz and numerical performance has been explored by Yang and co-workers.^131^ Later, the introduction of the GL limit (see contribution (2.4.4))^133^ in functional development has led to the definition of double-hybrid functionals, including some nonempirical approaches.^254–256^

Interestingly, since the introduction of Becke's half-and-half model, constraints derived from properties of the AC have been used for functional development, thereby avoiding introducing variables to be fitted to external (not theoretical) data. In other words, increasing the number of theoretical constraints in going from local to hybrid functionals leads to improved numerical performance (at least within the same functional family) for a large number of chemical properties.^257^

#### 3.1.11 Sun

In the approach of using exact constraints to construct DFAs mentioned in contribution (3.1.2), two different levels of exact constraints have been successfully used. For example, the PW91 GGA functional was constructed to satisfy the exact constraints of the exchange–correlation hole, while the very similar PBE GGA functional was constructed to satisfy exact constraints of the exchange–correlation energy. The SCAN meta-GGA functional was constructed by satisfying the exact constraints of the exchange–correlation energy but guided also by properties of the exchange–correlation hole.

#### 3.1.12 Gritsenko

A useful strategy in constructing approximations to the Kohn–Sham exchange–correlation potential is the statistical averaging of (different) orbital potentials (SAOP). The SAOP exchange–correlation potential, which statistically averages the potential with the correct Coulombic asymptotics and the potential arising from the step structure of the atomic and molecular electron shells, produces a good-quality estimate of vertical ionization potentials and yields a high-quality zero-order estimate of excitation energies within TDDFT.^258^

#### 3.1.13 Romaniello

The link between DFT and many-body perturbation theory (MBPT) based on Green's functions has been particularly beneficial. The Sham–Schlüter equation (SSE),^259^ which relates the Kohn–Sham potential of DFT to the self-energy of MBPT, has given several insights into approximations to *v*_xc_. As an example, one can easily retrieve the OEP equations from the linearized version of the SSE.^260,261^

Also, the time-dependent version of the SSE^262^ has been very useful in the context of TDDFT. For example, one can show that the TDDFT exchange–correlation kernel *f*_xc_ can be written exactly as two contributions, one responsible for the shift of the Kohn–Sham band gap to the fundamental gap and the other accounting for excitonic effects.^263^ This splitting has been recently used to calculate accurate optical spectra of semiconductors and insulators within a pure Kohn–Sham TDDFT framework – that is, without invoking empirical information nor theory beyond Kohn–Sham DFT (*e.g.*, *GW* theory) to correct the Kohn–Sham gap.^264^

#### 3.1.14 Galli

One of the outstanding open problems in defining approximate density functionals pertains to the description of the electronic properties of solid–solid and solid–liquid interfaces. When systems with different dielectric properties are interfaced – for example, a metal with an insulator or a semiconductor such as silicon with an insulating liquid such as water – none of the existing functionals can accurately describe band offsets and other electronic properties. This issue can be mitigated by carrying out *GW* calculations starting from DFT orbitals (for nonmetallic systems) However, this *GW*@DFT approach does not work when the underlying wave function provided by DFT turns out to be too inaccurate as a starting point – for example, for some transition-metal oxides.

A useful strategy for deriving approximate functionals for interfaces may be based on an approximate treatment of the screened Coulomb interaction and of dielectric matrices; the latter may then be used to derive approximate hybrid functionals with parameters that capture how the dielectric screening varies in different parts of the system (see, *e.g.*, ref. 265 and references therein).

### 3.2 How accurate do we need DFAs to be?

#### 3.2.1 Jones

Table 2 shows that “accuracy” has different meanings in different contexts. If one is interested in properties such as cohesive energies and structures in different phases of extended systems, then it is impossible in practice to determine accurate total energies using DFT methods. If the goal, however, is to shed new light on a problem or to make unbiased predictions, then DFT calculations can be a reliable partner. They share with other approaches the benefits of error cancellation, and users of molecular dynamics welcome the fact that forces are straightforward to calculate and consistent with variations in the energy.

**Table 2:** Common accuracy objectives

Property	Accuracy required
Heats of formation	1^a^ kcal mol^−1^
	3^b^ kcal mol^−1^
Heats of formation (“intensive”)^c^	0.3^a^ kcal mol^−1^
	1.6^b^ kcal mol^−1^
Conformational energies	0.1^d^ kcal mol^−1^
Barrier heights	1^e^ kcal mol^−1^
Ionization potentials	1^e^ kcal mol^−1^
Band gaps	0.1^f^ eV
Excitation energies	0.1^f^ eV
Bond lengths	1^g^ pm
Vibrational frequencies	<3^h^ cm^−1^
Shielding constants	0.5–5%^i^
Dipole moments	0.1–0.2^j^ D
Dipole polarizabilities	0.5–1^j^ a.u.
Electric field gradients	0.1–0.2^j^ a.u.

a Savin: mean value of the experimental uncertainties compiled in ref. 266 for over 500 molecules containing elements with *Z* < 18. See also ref. 267.

b Savin: *Q*_95_, *cf.* contribution (3.3.12), obtained from the experimental uncertainties compiled in ref. 266 for over 500 molecules containing elements with *Z* < 18.

c Savin: heat of formation divided by (the number of atoms −1), justified by the mean of the values obtained by detaching successively one atom after the other.

d Grimme: molecular total energy difference for the same covalently bound structure but with different three-dimensional shape normally obtained by rotation around covalent bonds.

e Schwerdtfeger: based on ref. 267.

f Kronik: an experimental accuracy of 0.1 eV in band gap measurements is possible, as well as desirable, but not at all trivial and may require the combination of several measurement techniques. Many reported experimental results, especially for insulators, do not necessarily reach this level of accuracy. Also, some reported band gaps arise from correction terms to optical gap values. Furthermore, experimental band gaps are also influenced by electron-nucleus coupling, sometimes quite significantly. This should be taken into account when comparing to results of electronic structure theory that do not include such coupling.

g Helgaker: the uncertainties in experimental bond lengths depend strongly on the experimental technique used – an accuracy of 1 pm for covalent bonds of first-row atoms is a reasonable target for computation. For benchmark data of wave-function methods, see ref. 268.

h Draxl: for vibrational frequencies, even semilocal DFT does already very well, if computed consistently (*i.e.*, for the optimized geometry^269^). The situation is more tricky for intensities, as these are typically not measured for solids. The situation may be different for molecules; thus a distinction would be needed. Note that intensities can’t be obtained by DFT alone.

i Kaupp: the necessary and achievable accuracy for shieldings and relative shifts differs from nucleus to nucleus and for different applications. The best way to report the accuracy that allows a comparison between different nuclei, is to give relative deviations in %, normalized to the shielding or shift range of a given nucleus (either computed or experimental). For meaningful accuracy, this should not exceed a few percent, sub-percent accuracy is better, and is achievable at least for light main-group systems. This is not yet the case for transition-metal nuclei.

j Schwerdtfeger: these accuracies are expected from any decent *ab initio* calculation. For comparable accuracies for EFGs achieved by coupled-cluster methods see ref. 270, for DFT see ref. 271 and 272.

In extended systems, it is often impractical to repeat calculations with different functional approximations [see contribution (2.1.16)], and it is essential to develop a level of “trust” [see contribution (3.2.7)] in the approximations one uses and a feeling for their limitations. My own applications over several decades show a clear preference for main-group elements, which might imply less trust in the ability of particular DFAs to describe transition and rare-earth elements. This is perhaps not surprising, since some DFAs describe energy differences in the corresponding atoms very poorly.

#### 3.2.2 Schwerdtfeger

The accuracy really depends on the property in question, on whether the corresponding operators sample the density more in the outer region or in the region close to the nucleus. I find Table 2 quite useful, but we should be reminded of some more problematic cases for properties such as polarizabilities or hyper-polarizabilities.

Moreover, if we have an incorrect long-range behaviour of the one-particle density, then the region close to the nucleus will also suffer because of charge conservation. As a result, properties like electric-field gradients (EFGs) are not so well described by currently available DFAs – the worst results are perhaps obtained for the late transition metals.^271^ To illustrate, the Cu EFG in CuF at the experimental bond distance is measured to be (in atomic units) −0.31(2), while some representative DFAs give +0.495 (LDA), +0.444 (PW91), and +0.146 (B3LYP). At the coupled-cluster CCSD(T) level, we have −0.439 (−0.341 if relativistic effects are included).^270^ To address this problem, the parameters in the CAMB3LYP functional can be tailored such that accurate results for EFG (and other short-range properties) are produced,^272^ but this is not a nice solution and no unique functional exists that performs well for all properties concerned.

One should also mention that, as for molecules,^273^ the performance of various DFAs for the solid state has been extensively analysed in the past – see, for example, ref. 274–276. Here, a few percent error range is typical for solid-state properties such as lattice constants, cohesive energies and bulk moduli if (for the heavier elements) relativistic effects and (for the lighter elements) phonon contributions are included. For finite temperatures, thermal effects need to be included as well.

#### 3.2.3 Fuentealba

Let me illustrate Schwerdtfeger's point regarding polarizabilities with some numbers. The dipole polarizability of a Li_4_ cluster has been calculated using the B3LYP and PW91 functionals, the values being (in atomic units) 355 and 394, respectively.^277^ But the experimental value is 327 – no explanation. More dramatically, for the Cu_9_ cluster, the calculated value is 295, while the experimental value is 984.^278^ Pathetic.

#### 3.2.4 Calaminici

The accuracies of static Kohn–Sham DFT dipole polarizabilities are usually in the range of 1%. However, the errors in dynamic dipole polarizabilities can be catastrophic – in particular, for planar conjugated systems. Here, the long-range behaviour of the functional used is critical.^279^ The polarizabilities of small metal clusters can be significantly influenced by temperature effects.^280^ Furthermore, the experimental references for static polarizabilities of such clusters are not always reliable. In particular, this is the case for the available measured copper-cluster polarizabilities in the literature.

#### 3.2.5 Chermette

As far as bond lengths are concerned, the accuracy can be better than 1 pm (perhaps has small as 0.3 pm) if the aim of the calculation is to compare bond lengths of organic isomers and conformers. On the other hand, if heavy atoms are present, 3 pm or even 5 pm can be considered fine, even with relativistic corrections added. There is room for improvement.

#### 3.2.6 Grimme

Chemically relevant energies span a huge range – from one tenth to hundreds of kcal mol^−1^. The often cited “chemical accuracy” of 1 kcal mol^−1^ usually refers to bond or atomization energies, which (for small systems) are on the order of a few hundred kcal mol^−1^. While this definition is appropriate for some thermochemical problems, it is inappropriate for others. For example, the very relevant conformational energies of typical pharmaceutical drug molecules with about 50 atoms are on the order of the thermal energy at room temperature (0.6 kcal mol^−1^). Hence, in practical applications, only errors less than about 0.1–0.2 kcal mol^−1^ are acceptable.^281^

Another aspect to consider here is that the most important primary application of current Kohn–Sham DFT in chemistry is probably the determination of equilibrium structures (*R*_e_) including those of chemical transition states. Even with rather simple DFAs (*e.g.*, GGAs), basically no severe outliers are obtained, even in electronically difficult cases – a fact that, in retrospect, was extremely important for the development of computational chemistry. As such, newly proposed, empirical DFAs, should be carefully tested not only for energies but additionally for the computation of *R*_e_ structures. Similar considerations hold for the computation of vibrational frequencies, which are of utmost importance for thermostatistical properties – for example, Gibbs free energies.

#### 3.2.7 Savin

It seems to me that in most cases, calculations – like experiments – are not carried out to obtain specific numbers, but to answer some questions. Furthermore, the methods of quantum chemistry do not provide error bars for our calculations. The expected accuracy is therefore what we have from our experience with methods, which may not apply to a specific case. This experience may be tainted by trust acquired over years and not revised by an active following of progress in the field.

Another aspect is that we may overemphasize the accuracy of what we take as a reference. Herbstein discusses several factors that may affect the measurement of such basic data as single-crystal unit-cell dimensions.^282^ Cioslowski *et al.* show that experimental error bars are often missing or can be quite large.^266^ Sometimes advanced wave-function calculations are not pushed far enough to be used as a reference.^283^

#### 3.2.8 Adamo and Ciofini

In some cases, determining how accurate DFAs need to be is probably even more difficult than determining how accurate existing DFAs are in fact for a given property. Indeed, even for a single, well-defined property, the target accuracy will depend on which question we aim to answer (as already pointed out by Savin in contribution (3.2.7)). The necessary accuracy will depend strongly on the type of “interaction with the real world” is desired, following the excellent classification given by Kronik in contribution (3.8.5): confirmation, interpretation, or prediction. In our experience, this is particularly true for the interpretation and prediction of excited-state properties of molecular systems.

There have been a huge number of publications assessing the performance of different DFAs within TDDFT for the prediction of excitation energies both using theoretical and experimental reference data – see, for instance, ref. 284–288. Nonetheless, two difficulties are becoming nowadays evident: the reliability of affordable theoretical reference methods for large molecular systems may be difficult to assess, and the fact that excitation energies may not be the only property needed to provide a full answer to a given chemical question.

Concerning the first point, thanks to a number of detailed studies that compare DFAs results with those obtained using different reference methods, it has become evident that, especially for complex molecular systems, assessing the accuracy of DFAs is also dependent on the choice of reference. By targeting an accuracy below a certain threshold in the excitation energy, one is probably simply targeting the error bar of the methods used as reference.^289^ Furthermore, errors depend on the type of excitations considered – for instance, local or charge-transfer excitations. Due to the different impact of the approximation used in a given DFA on the different types of excitation, it is very difficult to assess a global accuracy for this DFA in predicting excitation energies. More severely, (vertical) excitation energies are often not what one aims for as a chemical answer since the quantitative description of the photophysical properties of a given molecular system are related to the prediction of its entire spectrum (absorption or emission), practically manifested in the observed colour.^290^ In this case, the accuracy we would like to reach – and which is asked for in industrial application, for instance – is the sensitivity of human colour perception.^291^

To achieve this objective, one needs to combine a very high (and energy-dependent) accuracy in the excitation energy with a very good description of the band shape. This latter is mostly obtained using approaches enabling the description of the vibrational broadening, that is the vibronic coupling between ground and excited states. Previous studies^291^ have nonetheless demonstrated that the same DFA can seldom reproduce with the same accuracy both electronic excitation energies and vibrational broadening. Finally, the comparison with experimental data can become even more complicated if environment effects, usually modelled with approximate methods, have to be considered.^292^ This latter point is of course of relevance, for any theoretical approach used and not exclusively limited to DFT.

#### 3.2.9 Barone

Sufficiently accurate molecular structures are a prerequisite for the computation of thermodynamic, kinetic, and spectroscopic properties. In this connection, the latest-generation DFAs (hybrid and, especially, double hybrids) with dispersion corrections added perform a remarkable job for main-group elements, in noncovalent complexes and for transition states.^293–295^ Furthermore, the remaining errors are rather systematic and can be corrected for by linear regression, depending only on the atomic numbers of the involved atoms.^296,297^ As a matter of fact, energies and properties can usually be calculated very accurately at DFT geometries with negligible errors provided that the functional and the basis set are properly selected. The situation is more involved for transition metals, where comprehensive benchmarks are still missing.

From another point of view, comparison with experiment requires vibrational corrections to geometric parameters and zero-point energies. The situation for main-group elements is again very satisfactory, with latest-generation DFAs in conjunction with second-order vibrational perturbation theory (or anharmonic treatments) providing remarkably accurate results without the need for any scaling factor.^294,296^ The above remarks concern isolated molecules (or low-pressure gas-phase). The situation is more involved in condensed phases, where much work to improve the accuracy of the results is ongoing.

#### 3.2.10 Piecuch

While it is important to set accuracy targets for any quantum-chemistry approach, not only for methods based on DFT, it may be useful to keep in mind that some quantities, such as binding energies in weakly bound clusters, activation energies, and vibrational frequencies, to name a few examples, vary so much among the various systems (in the case of vibrational frequencies, even within a given system) that setting up fixed error limits is not necessarily helpful. In all such cases, the relative (percent) errors may be more informative when setting up accuracy standards.

For example, it is commonly accepted that achieving a 1 kcal mol^−1^ (chemical) accuracy for binding energies involving covalently bound molecular species is often desirable, but setting a similar accuracy target for activation energies, which can be on the order of 1 kcal mol^−1^ in some processes and more than 10 kcal mol^−1^ in others, may be misleading. Furthermore, one can have reaction mechanisms that involve larger and smaller barriers along the same reaction pathway or along multiple competing pathways. A 1 kcal mol^−1^ accuracy level does not work well for noncovalent interactions either. In fact, even the frequently mentioned value of about 0.1 kcal mol^−1^ may not be adequate in this case.

If we replace the error criteria for the binding energies in weakly bound species and activation energies by relative errors of, say, 5%, then we may be in a better position to judge and make recommendations regarding what method to use. Indeed, if the activation energies along the reaction pathway of interest are on the order of 10 kcal mol^−1^ or more, as in ref. 298, then ∼5% relative errors translate into total errors on the order of 0.5–1 kcal mol^−1^, which is good enough to understand the reaction mechanism. However, if the activation barriers are on the order of 1–5 kcal mol^−1^, or if we must decide if a particular reaction has a barrier or is barrierless, as in ref. 299, then a fixed accuracy criterion may be insufficient to make a proper recommendation regarding what method to use. Similarly, a fixed 0.1 kcal mol^−1^ criterion might be of little use for some noncovalent interactions, where there are many cases in which the interaction energies are as small as 1 kcal mol^−1^ or less and equally many cases in which the interaction energies are on the order of a few kcal mol^−1^ or more. A good illustration of the former situation is the magnesium dimer, which is an important weakly bound system in studies of ultracold and collisional phenomena and which is characterized by a binding energy of about 430 cm^−1^; see ref. 300 and 301. To properly understand this system, one must be able to reach an accuracy on the order of a few cm^−1^, which is a major undertaking, even for the highest levels of *ab initio* wave-function theory.^301,302^

The magnesium-dimer example is also a reminder that in setting accuracy targets, we should be careful about treating wave-function methods, including those based on coupled-cluster theory, as providers of reliable reference information. As is very well known, wave-function methods exhibit a much slower convergence with the basis set than methods based on DFT. Also, the treatment of core electrons is usually different in DFT and wave-function calculations. Wave-function calculations are often carried out with frozen core electrons, whereas DFT (putting aside the issue of relativistic effective core potentials) is an all-electron theory. Thus, judging DFAs by comparison with wave-function approaches may sometimes be misleading or questionable.^303^ Finally, the CCSD(T) approach, often regarded as a standard for high-accuracy calculations, fails not only in multireference situations, such as covalent bond stretching and biradicals, but also in many cases of noncovalent interactions, including the aforementioned magnesium dimer, where the CCSD(T) binding energy extrapolated to the complete basis-set limit has a substantial error.^301,302^ While the development of *ab initio* wave function methods can be well served by comparisons with FCI, the development of DFAs may be better served by comparisons with reliable experimental data.

The idea of setting up accuracy targets using relative (percent) errors may easily be extended to other properties in Table 2. For example, the aforementioned 5% error limit would work well for vibrational frequencies, including low-frequency and high-frequency modes. Clearly, depending on the nature of the application, one may replace the 5% target by a different target.

#### 3.2.11 Kaupp and Arbuznikov

Several contributions in this section indicate that accuracy depends on the type of property one looks at. While highly empirical DFAs have concentrated on relative energies relevant for chemical processes, a wide-ranging recent discussion has put electron densities into focus.^304–311^ Here, we should clearly distinguish different spatial regions in an atom, molecule or solid, as different requirements hold for the core, valence, asymptotic, and intermediate regions. For example, many (albeit not all!) empirical Minnesota functionals, which give excellent valence energies and probably reasonable valence densities, produce highly erratic hyperfine couplings for transition-metal nuclei^312–314^ and also perform poorly for NMR shifts and spin–spin coupling constants.^315–317^ A position-dependent admixture of Hartree–Fock exchange in local hybrid functionals^318,319^ seems to be one way to improve specifically properties of operators that act near the nuclei or far away from them – see contribution (4.1.10).

#### 3.2.12 Trickey

One of the most striking features of Table 2 is what is not there. Except for band gaps, there is nothing about solids, no cohesive energies, no bulk moduli, no crystalline phase-transition pressures. (We here assume that “bond lengths” can be interpreted generously as including lattice constants.) With the disclaimer that what follow are simply values that seem to be fitting from experience but not from study, plausible useful accuracy values seem to be 0.015 Å for cell constants, about 0.1 eV per atom for cohesive energies, ±4% for bulk moduli, and ±2% for transition pressures (assuming the crystal structures are correct). The main point is that work is needed on such criteria.

Add to that something little discussed in this roundtable – namely, that predictive screening of materials requires even-handed accuracy across states of aggregation. One must have the same computationally affordable functional and protocols for both the isolated molecular constituents and the condensed phases, with correspondingly consistently appropriate accuracy for both constituents and aggregates. It is of little or no use in first-principles computational materials physics to prescribe a highly sophisticated DFA of great accuracy for the molecular constituents that cannot be afforded in condensed-phase studies or is deliberately tuned (*e.g.*, OPTX) to be accurate for molecules only.

#### 3.2.13 Baerends

A striking deficiency of almost all DFAs is the error of about 5 eV in the orbital energies. This is a much larger error (more than 100 kcal mol^−1^!) than in the total energy and unacceptable in view of the desired chemical accuracy. Its origin can be clarified using the partitioning of the exchange–correlation potential in the hole potential part and response part, *v*_xc_ = *v̄*^hole^_xc_ + *v̄*^resp^, where the overbar indicates that we are dealing with coupling constant integrated quantities; see Section 2.4. The exchange–correlation hole potential is directly related to the exchange–correlation energy density, *v̄*^hole^_xc_(**r**) = 2*ε*_xc_(**r**) with 
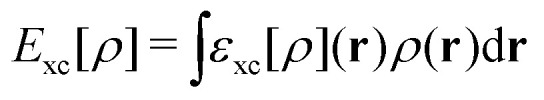
. The response part originates from the functional derivative of the *ε*_xc_ factor in the total energy.

Given DFAs with good total energies, the error in the orbital energies should not come from the hole part of the exchange–correlation potential – indeed, it has been argued that the error is in the response potential.^320^ It is quite common that an approximation to an integrand is decent in the sense that the integral (the energy) is well approximated, while the derivative of the integrand (the potential) is still very poor.

The response part of the current DFA potentials is too repulsive over the bulk molecular region, causing the 4–6 eV upshift of the orbital levels. A better approximation to the response potential is called for, rather than just the derivative that arises from existing LDA or (meta-)GGA energy density approximations. Indeed, replacing the LDA/GGA response part of the potential with the approximate response potential from ref. 321 (a local potential determined from nonlocal input) already improves the orbital energy spectrum a lot.^320^ Better approximations to the Kohn–Sham potential of course also improve response properties such as (hyper)polarizabilities and excitation energies.^258^ Note that this improvement is not primarily an effect of the correct asymptotic behaviour since the orbital energies are mostly determined by the potential in regions where the orbitals have a large amplitude (*i.e.*, the region where the bulk molecular density resides). Obviously, the accuracy of the DFA potential has been lagging far behind that of the energy. It needs to be improved, preferably in a more fundamental way than by pragmatically admixing some percentage of a nonlocal exchange potential.

#### 3.2.14 Görling

The origin of the errors that most DFAs exhibit for the Kohn–Sham orbital energies is the presence of unphysical self-interactions. A solution to this problem has been around for a long time – namely, an exact treatment of the Kohn–Sham exchange potential, which requires the OEP method.^40–46^ If the exact local Kohn–Sham exchange potential – that is, the OEP exchange potential – is used, then the HOMO eigenvalue immediately is close to the IP as it should, whereas, in conventional GGA calculations, it is typically several eV too high. Moreover, the Kohn–Sham eigenvalue spectrum changes qualitatively: an exact-exchange (EXX-OEP) calculation gives a Rydberg series as it should, while a GGA calculation does not. Thus, if the self-interaction contained in the Hartree potential is properly cancelled by the exact Kohn–Sham exchange potential, then a qualitatively correct and quantitatively much more accurate spectrum of Kohn–Sham orbital energies is obtained.^42–46^

The OEP method has a bad reputation because of numerical problems. However, these problems have been solved and computationally efficient, numerically stable OEP methods are now available.^46^ An exact treatment of exchange requires correlation functionals that go along with it. Such correlation functionals exist – for example, RPA-based functionals^102–104^ – but are so far not very popular. The poor orbital energies are thus the price to pay for approximating exchange and correlation together in most DFAs, in order to exploit error cancellations.

#### 3.2.15 Neese

I very much welcome this discussion. In practice, there is a large SIE. This error is large and profoundly influences the localization/delocalization of the Kohn–Sham orbitals and consequently, the properties derived from them and the associated electron density. We know that removing the SIE using established methods like the Perdew–Zunger scheme^10^ destroys much of the accuracy of Kohn–Sham DFT. The development of physically based correlation functionals becomes challenging with this error in the background.

I am aware of brilliant attempts to develop correlation functionals on top of self-interaction free references (discussed in the preceding contribution by Görling). I would be delighted to see this approach receive even more attention in functional development.

#### 3.2.16 Baerends

To further this discussion, let me note that it is indeed generally accepted that the origin of the poor orbital energies of presently available DFAs are the unphysical self-interactions. However, it is not completely clear what is meant by SIE. The one-electron SIE is felt to be evident: the exchange–correlation energy of a one-electron system like the H atom (in this case just the exchange energy) should cancel the Hartree energy. Actually, LDA is not so bad for the H atom: the Hartree energy of 8.01 eV is cancelled to a reasonable degree by the LDA exchange energy of −6.89 eV, yielding an error of only 1.12 eV. If we add the B88 GGA gradient correction for the exchange energy, then the SIE is reduced to 0.04 eV. The same very small SIE is observed for the H_2_ molecule at the equilibrium distance.^320^

This should give us pause for thought when we want to blame the SIE for failures of DFAs. How much SIE is there really in the current DFAs? The DFA error in the orbital energy is of a different order of magnitude: 6.88 eV above the exact H-atom value with LDA and 6.20 eV when the B88 gradient correction is added – clearly, not the same effect as the tiny SIE in the total energy. The effect on the orbital energies that we call SIE arises when we take the functional derivative of the energy (it is in *v*^resp^).

A general definition of SIE is not so easy to formulate. In the original Perdew–Zunger work, the total energy was taken as starting point for constructing the correction.^10^ The most important effect of the Perdew–Zunger correction was, however, to change the potential and most of their discussion was focused on orbital energies. A straightforward definition of the SIE (also in the many-electron case) would be the error incurred by the exchange–correlation hole not integrating to −1 electron. Now, the first exact property that is required of the model holes in DFT is that they do integrate to −1 electron, as is already true for Slater's *ρ*^1/3^ approximation. This does indeed lead to reasonable results for the total energy but does not guarantee a good potential because of deficiencies that appear in the step to the corresponding potential.^320^

Using exact exchange (100% or at least a very large percentage) in either a generalized Kohn–Sham or EXX-OEP manner also provides a large improvement in the orbital energies, as noted in contribution (3.2.14). The improvement is due to the way the step to the potential works out in that case, as it does for the Hartree–Fock-like correction in the Perdew–Zunger self-interaction correction.

#### 3.2.17 Gidopoulos

In our group, we share the view that self-interactions are behind the errors of Kohn–Sham orbital energies, an opinion advocated strongly also by Rod Bartlett.^322^ As Baerends explains, the effects of self-interaction are not evident in the DFA total energy and the error in the Kohn–Sham orbital energies is about an order of magnitude greater than the error in the total energy. So why blame self-interaction for the Kohn–Sham orbital error? Our reasoning for arguing that the errors in the Kohn–Sham orbital energies are due to self-interaction, when even the definition of self-interaction in the total energy is unclear, is simple.

Görling was the first to point out that we can use Poisson's equation to define an effective charge density from the Kohn–Sham potential.^43^ Then, the Laplacian of the Kohn–Sham Hartree-exchange–correlation (Hxc) potential *v*_Hxc_(**r**) defines unambiguously an effective charge density *ρ*_rep_(**r**) whose Coulomb potential is the Hxc potential:24
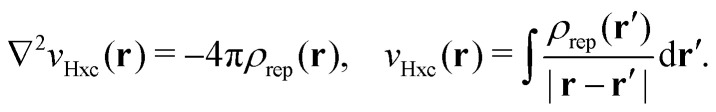
The “repulsion” or “screening” density *ρ*_rep_(**r**) effectively mimics the repulsion felt by each electron. For a system of *N* electrons in a self-interaction-free theory, the integrated charge *Q*_rep_ of *ρ*_rep_(**r**) should be *Q*_rep_ = *N* − 1, because each electron is repelled by the other *N* − 1 electrons but not by itself. However, in local and semilocal DFAs, *Q*_rep_ = *N*, which we interpret to imply that each Kohn–Sham electron is effectively repelled by all electrons of the system, including itself, and so self-interaction is present.

We agree with Baerends that the quality of the total energy in local and semilocal DFAs is (far) superior to the quality of the Kohn–Sham potential and hence we have decided not to interfere with the total energy of the DFA. Instead, we impose constraints on the effective local potential to reduce the self-interaction errors from it. These constraints, *Q*_rep_ = *N* − 1 and *ρ*_rep_(**r**) ≥ 0, are enforced with the OEP method, whose mathematical (rather than numerical) problems with finite basis sets are now well understood.^323,324^

The computational cost of these OEP calculations is determined by the matrix elements of the DFA functional derivative, a local potential, and is comparable to performing a small number (about ten) of DFA calculations. Imposing these constraints, the error of the HOMO Kohn–Sham orbital energies for local and semilocal DFAs reduces to about 1 eV. For one-electron systems, the two constraints give correctly a zero Hxc potential.^325,326^

#### 3.2.18 Kronik

Following the important points raised in contributions (2.1.7) and (3.2.17), I think it is worthwhile to emphasize that piecewise linearity, freedom from self-interaction, and an asymptotically correct Kohn–Sham potential – all three of which are important principles for DFA construction – are somewhat related yet inequivalent properties of the exact density functional.^327^

#### 3.2.19 Xu

The IP and EA are fundamental properties of atoms, molecules and solids, which are often associated with the orbital energies *via* Koopmans' theorem^328^ in Hartree–Fock theory or Janak's theorem^87^ in Kohn–Sham DFT; see contributions (2.2.4), (2.4.9), and (3.2.13). However, it is well-known that relaxation and correlation effects are often important in electron detachment and attachment processes, calling for extensions to the theory.^16,329–334^

From the perspective of fractional charges,^16^ an integration approach has been developed for the double-hybrid functionals,^331^ whose justification lies in the fact that they are found to fulfil better the piecewise linearity condition (see contribution (2.4.1)) and suffer less from delocalization error.^332^ Furthermore, the extended Koopmans' theorem^333^ (EKT) can be applied to the double-hybrid (DH) functionals, leading to the EKT-DH methods,^334^ which are shown to be capable of describing the breakdowns of the quasi-particle approximations for the inner-valence IPs, at a relatively low computational cost and a high accuracy.

### 3.3 How should we validate the quality of DFAs?

#### 3.3.1 Staroverov

In *ab initio* methods, quality is synonymous with overall accuracy. The current state of DFT suggests that one may need at least two interconnected criteria to characterize the quality of DFAs: accuracy and mathematical rigour (*i.e.*, the extent to which the DFA satisfies exact constraints), which is a proxy for universality. Of course, the choice and relative importance of various exact constraints, test sets, metrics of accuracy, *etc.* are subjective, but some consensus may not be impossible to reach.

#### 3.3.2 De Proft and Geerlings

A remark aside from the validation itself is the use of a certain DFA after its validation. It can be asked, once a given DFA has proven its merits in a certain domain, that this DFA should additionally be benchmarked for problems (compounds, properties, reactions) that do not differ markedly from those for which the DFA has been successfully validated in the literature and for which one can reasonably expect that it will perform well for the problem at hand (*cf.* the notion “level of trust” introduced in contribution (3.2.1)).

This procedure, sometimes requested by reviewers, is often time consuming. In addition, if one asks authors to benchmark each DFA for every type of compound in every type of reaction, it can be argued that one is perhaps approaching a new level of parameterization.

#### 3.3.3 Gill

The development of a small number of data sets of unimpeachable experimental data, against which the predictions of new DFAs can be compared objectively and reproducibly, is essential. Both the data sets and the software used to evaluate DFA performance should be freely accessible and entirely transparent. It was the publication of such comparisons in the early 1990s that led to the widespread adoption of DFT in the chemical community.^335^

#### 3.3.4 Gould

While not quite as robust as experimental data, we now have access to some impressively large quantum-chemical benchmark sets (about 5000 energy differences in MGCDB84^273^ and 1500 in GMTKN55^336^) against which to validate DFAs. However, key overall quality metrics can be reproduced almost perfectly by about 150 entries of the original benchmark sets (less than 10%).^337^ This means that a large part of these sets contain redundant information. It is therefore important that validation protocols test DFAs across diverse physics and chemistry – we should not just assume that testing against more systems will automatically do a better job. New applications of statistical techniques may be required to develop robust validation protocols.

#### 3.3.5 Grimme

Validation of DFAs on benchmark sets outside the common chemical compound space is essential if we are to find universal and practically robust methods. Automatically generated molecular structures – as employed, for example, in the “mindless benchmarking” scheme proposed in ref. 338 – may offer a solution to this problem.

#### 3.3.6 Krylov

Extending the validation studies to more properties is important – for example, what works well for dipole moments may not give you good polarizabilities, and so on.

#### 3.3.7 Neese

I completely agree with the comment of Krylov. It seems to me that enormous efforts are directed towards developing functionals that provide good total energies and significant progress in this direction is undeniable. Yet, there are many other properties of chemical interest. In addition, accuracy in total energies does not translate to accuracy in other properties – for example, we have frequently seen that some popular functionals that provide good total energies fail spectacularly in the computation of hyperfine couplings, excitation energies or other spectroscopic properties (as eluded to in contribution (3.2.11)). At this point, co-convergence of energy and properties appears to be exclusive to wave-function-based *ab initio* methods.

The development of standardized test sets has been very beneficial for the development of DFT. It would seem beneficial, yet challenging, to include a wide range of additional data in the development of new functionals to come closer to co-convergence.

#### 3.3.8 Trickey

Regarding validation construed broadly: what constitutes a meaningful, hence valid, improvement in a DFA? If a DFA improves over another by 0.2 kcal mol^−1^ mean absolute error (MAE) on atomization energies and 0.02 Å on bond lengths, *etc.*, is that really an improvement or is it in the noise of the data sets themselves? If my group produces a DFA that gives essentially the same errors on a large collection of canonical data sets as the best older DFA on the same Perdew–Schmidt rung,^339^ but the new DFA is much more stable numerically or 20% faster, wouldn’t such improvements be validation themselves?

#### 3.3.9 Savin

True, the superiority of one DFA over another can be significantly diminished after taking into account the uncertainty in the reference data.^340^

#### 3.3.10 Barone

Ideally, a DFA should provide accurate results for a broad set of molecular properties. However, from a pragmatic point of view, there is a difference between specialized and broadly applicable DFAs and the choice between the two classes depends on the problem at hand. My personal view is that the most suitable strategy is to enforce the largest possible number of formal constraints that a DFA should obey while leaving a few parameters free for improving accuracy. However, at present, DFA benchmark is severely biased towards energies and (perhaps) first-order properties of molecules containing atoms belonging to the first three rows of the periodic table.

#### 3.3.11 Pernot

To answer the question of Trickey about the intercomparison of DFAs, a small difference in MAEs between two DFAs certainly cannot be relied upon without additional information. This difference might be an artefact of the limited size of a nonexhaustive reference data set. As a result, there may be a high probability of rank inversion when the data set is perturbed by adding or suppressing a few points. A set of tools has recently been proposed to address this problem – for example, by estimating a rank inversion probability, *P*_inv_, or by using statistics based on system-wise comparisons, such as the systematic improvement probability.^341,342^

#### 3.3.12 Pernot

Validation requires the comparison of calculated values with a set of high-quality reference data. The resulting errors are used to estimate validation statistics. An important fact to have in mind is that the distribution of errors has no reason to be normal,^343^ essentially because the errors are dominated by systematic contributions from all the approximations involved in a calculation – level of theory, basis set quality, values of parameters, and so on.^344^

This is why statistics such as the MAE should not be used to estimate the confidence level of a DFA. There are more pertinent metrics such as *Q*_95_ (the 95th quantile of the absolute error distribution), on which a level of confidence can be based.^343,345^ We then know that there is a 5% risk to get an absolute error above *Q*_95_. By comparison, the probability for absolute errors to be above the MAE has been observed to vary between 0.2 and 0.45.^346^ From a MAE value of let us say 0.5 kcal mol^−1^, one cannot estimate the probability/risk for the errors to exceed 1 kcal mol^−1^.

The confidence we have in the prediction capabilities of a given method has something to do with probabilistic forecasting; this however, may depend on factors difficult to quantify, such as deciding about the probability we consider good enough to take a decision. Ideally, a DFA should be judged on its prediction uncertainty, like any measurement method, but this requires the correction of systematic errors, which goes beyond the realm of the DFA itself.^347–349^

#### 3.3.13 Savin

Could it be that the importance of accuracy is overemphasized? Let us take as an example the intensive atomization energies (*i.e.*, per atom) as obtained with the B3LYP functional on a widely used benchmark data set.^350^ Let us now consider what the measures mentioned in contribution (3.3.12) provide.^343^ The often quoted “chemical accuracy” of 1 kcal mol^−1^ corresponds to the mean absolute error of the B3LYP functional. However, this accuracy is reached by only about half of the systems in the data set.

Let us now ask ourselves what is smallest target accuracy that is reached by 95% of the systems in this data set. It turns out that, to satisfy this condition, the target accuracy must be as large as 3–5 kcal mol^−1^, the presence of the interval arising from the finite size of the data set. Nevertheless, the B3LYP functional is a very successful DFA.

#### 3.3.14 Neese

It appears to me that “sufficient accuracy” is something that depends largely on the context of the computational problem at hand. Take a very exothermic reaction, with a free-energy change of around −50 kcal mol^−1^. I don’t think I would understand anything about this reaction any better whether I compute the energy change to be −47 or −53 kcal mol^−1^, nor would it change any conclusion. On the other hand, if one wants to correctly predict the enantiomeric excess of a bifurcating reaction that may lead to different stereochemical outcomes, then even one half of kcal mol^−1^ matters.

Related to this problem of adjusting the “useful” accuracy to the problem at hand is the question of what else is needed for a successful chemical prediction or interpretation? For example, we have learned the hard way that getting accurate electronic energies from coupled-cluster theory will not necessarily lead to more accurate chemical predictions. In real-life chemical applications, there are other important error sources, for example coming from solvation or entropy effects. In addition one can not stress enough how important it is to carefully construct the computational model. In studying complex molecules (or enzyme active sites), one needs to pay a great deal of attention to possible alternative conformers, alternative protonation states, possible hydrogen bonds or potentially functionally important solvent molecules, to name only a few important aspects of model construction. In our experience, the errors stemming from failing to treat any of the mentioned effects correctly can easily overwhelm the error in the electronic energies. If this is the case, focusing on computing accurate electronic energies on irrelevant chemical models or with a large, possibly unrecognized solvation energy error in the background appears rather pointless – the conclusions drawn from the calculations will likely be wrong or will fail to properly explain the experimental findings. These aspects require a great deal of chemical common sense on the part of the computational chemist and are independent of the intrinsic accuracy of the chosen theoretical method.

#### 3.3.15 Galli

The validation of DFAs requires first a serious verification effort. This verification should include comparisons of properties obtained with different codes, taking care to ensure convergence of all numerical parameters involved in the calculations.

Sometimes statements on the validity of DFAs made in the literature are inaccurate or just not correct because a detailed analysis of numerical approximations has not been carried out before assessing the accuracy of the DFA. It is important to realize that the impact of poorly converged numerical calculations on the assessment of the validity of the theory is not the same for all properties and hence numerical verification should be done for each property of interest separately. It is also important to keep in mind that many comparisons of various DFAs in the solid state are made using pseudopotentials that are not consistent with the level of theory adopted for the valence electrons. For example, almost all hybrid DFT calculations are carried out with PBE pseudopotentials; and all comparisons are thus tainted by this inconsistency.

#### 3.3.16 Galli

We should push for a much more concerted effort of the different communities using DFT, aimed towards the verification and validation of properties calculated with DFAs. Different communities of DFT users, with different “cultures”, still exist: the quantum-chemistry community, the solid-state and materials science community, and the community carrying out first-principles MD and interested in finite-temperature properties. It would be very important for these communities to come together and establish a list of properties, comparing results for molecular and solid-state systems.

### 3.4 Where do existing DFAs work and where do they fail?

#### 3.4.1 Johnson

Existing Kohn–Sham DFAs fail for systems where the orbital occupations cannot be represented by a single Slater determinant. The classic example of a multireference system that cannot be represented by a single Slater determinant is the stretched H_2_ molecule. The ground-state wave function in the dissociation limit is an equal mixture of two determinants. A simple single-determinant wave function from MO theory incorrectly includes both covalent and ionic terms and, consequently, gives in the dissociation limit an energy of only −11/16*E*_h_, compared to the exact energy of −1*E*_h_. Despite not using an explicit wave function, Kohn–Sham DFAs are valid only for single-determinant states and suffer from similar errors as Hartree–Fock-based wave-function methods for multireference systems.^121^ This can be understood by examination of the pair density. For a Slater-determinant wave function, the pair density is25
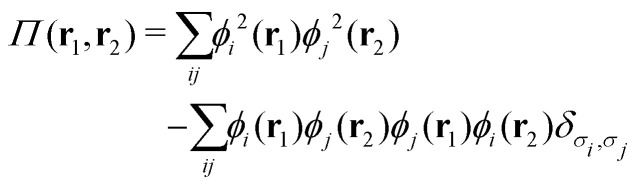
where the sums run over pairs of occupied orbitals *ϕ*_*i*_ of spin *σ*_*i*_. The exact pair density is the probability of finding a pair of electrons simultaneously at two points in space; it determines the proper form of the exchange–correlation energy *via* the AC.

Notably, the form of the pair density gives rise to the exchange hole in the case of parallel spins (*δ*_*σ*_*i*_,*σ*_*j*__ = 1) and leads to a depletion of probability of finding a second, same-spin electron near a reference electron. However, for multideterminental wave functions, the pair density behaves quite differently. Instead of leading to a Fermi (exchange) hole, with a depletion of parallel-spin electron density around a reference point, multireference systems can instead have a Fermi “heap” around the reference point,^351^ with an accumulation of parallel-spin electron density. This physics is not captured by Kohn–Sham DFAs, which model a localized exchange hole.

While there are other types of systems (such as those exhibiting significant delocalization error) where a particular DFA or class of DFAs may fail, all Kohn–Sham DFAs fail for multireference systems. Examples include stretched covalent bonds,^352^ organic biradicals,^353^ and solid Au_2_S.^354^

#### 3.4.2 Rebolini

Although Kohn–Sham theory is in principle valid for all systems, in practice existing DFAs mostly fail at describing multireference systems. However, one may want to distinguish between properties that depend on the total density of the system, which may still be properly described, and properties which depend specifically on the strongly-correlated electrons where DFAs are almost “expected” to fail – for instance, DFAs can be used to study the equilibrium structure, phonon spectrum, and polarization of strongly-correlated materials but fail to describe most magnetic properties.

#### 3.4.3 Neese

The first thing that comes to my mind in this context is the multiplet problem – that is, the simple fact that a single electron configuration (meaning a distribution of electrons among orbitals with occupation 0, 1 or 2) gives, in general, a number of different many-particle states with different spin couplings among the unpaired electrons.

This is not an esoteric formal remark. In open-shell transition-metal complexes, for example, the multiplet problem is extremely prevalent and affects all of their physical (spectroscopic) properties in a profound way. Take a simple L-edge (2p → 3d) excitation. In a d^5^ system, this excitation leads to as many as 1512 different final states that all contribute to the L-edge absorption spectrum. Yet, in a particle–hole theory such as TDDFT, one only has 15 particle–hole pairs to work with. How to describe 1512 states with only 15 particle–hole pairs is not clear. In practice, the failures are dramatic. Another example are the d–d spectra of these ions, which frequently show low-lying double excitations that are completely absent from the DFT-computed spectra.^355^

Similar remarks hold for many multideterminental (as opposed to multiconfigurational) problems, like spin-coupled open-shell ions. Surely, broken-symmetry DFT is a highly useful tool, yet it is a bit of a crutch and I am not aware of a satisfactory formal solution that would also be practical.

I find it important to distinguish between multideterminental and multiconfiguration problems. In the former case, there is a single electronic configuration but spin coupling of the unpaired electrons leads to a multideterminental wave function. In the latter case, there is an actual mixing of configurations with different orbital occupations. The former case is far easier and far more frequent. Recognizing this distinction may help (and, in fact, has already helped) to design more tailored approximations – for example, spin-flip methods.

#### 3.4.4 Ayers

Multireference effects are obviously problematic in single-determinant theories like Kohn–Sham DFT. But even if one changed to a different starting point (perhaps by moving to an extended DFT), there would still be (different) types of correlation that would be difficult to describe. Indeed, for every practical electronic-structure method I know, I can think of some type of electron correlation that it struggles to describe, so *ab initio* DFT^356^ is no solution. I would not say the situation is hopeless, but I accept that different flavours of DFAs will be needed for different types of properties and systems.

#### 3.4.5 Piecuch

This clearly is a rich topic, and all of us could find examples of situations (molecular systems, solids, selected properties other than energy, *etc.*) where the existing functionals used in conjunction with Kohn–Sham DFT and TD-DFT struggle. Bond breaking, doubly excited and charge-transfer states, strong correlations, and dispersion forces require additional – sometimes a lot of additional – effort beyond conventional Kohn–Sham DFT computations. However, from my point of view, which is the point of view of an ordinary user of DFT codes, a larger issue is the lack of transferability of DFT-based recommendations.

Focusing on my own experiences, the widely used B3LYP functional is among the best DFAs for studies of the activation barriers that determine aerobic oxidations of alcohols by gold nanoparticles,^298^ the BP86 and B97-D functionals performing considerably worse. At the same time, B3LYP and other hybrid functionals work poorly and the BP86 (especially when corrected for dispersion) and B97-D functionals are impressively accurate in applications of DFT to methyl–cobalt bond dissociation and low-lying excited states of methylcobalamin.^357,358^

The latter situation is similar to that created by the application of various DFAs to dicopper–dioxygen structural motifs. For example, when examining isomerization curves connecting the bis(μ-oxo) and μ-η^2^:η^2^-peroxo isomers of Cu_2_O_2_ cores supported by 0, 2, 4, and 6 ammonia ligands, hybrid functionals fail, the magnitude of the error being directly proportional to the percentage Hartree–Fock exchange in the functional.^359^ Pure GGAs work well in this case.

There is nothing new in the observation that pure GGAs may work better in situations involving static correlation. However, improving predictability of the outcome of DFT computations, so that one could, for example, avoid calibrating DFT functionals every time a new system is studied, while addressing fundamental issues such as the issue of SIE, would be useful. I realize that there has been great progress in addressing these and related issues in all sorts of interesting ways, but an additional effort toward improving the situation in this area would be helpful for the users of DFT methods.

#### 3.4.6 Gagliardi

Another application where Kohn–Sham DFT encounters some challenges is the determination of the spin ladder in multimetallic compounds. Some of these compounds are molecular magnets with potential applications in information storage, quantum computation, and molecular electronics.^360^ In compounds containing several magnetic centres, the spin carriers can magnetically interact in many ways. A prototypical system is a tris-hydroxo-bridged Cr(iii)–Cr(iii) system (Kremers dimer),^361^ which consists of two antiferromagnetically coupled Cr(iii) metal centres with a d^3^–d^3^ electron configuration.

Pantzasis^362^ pointed out that Kohn–Sham DFT generally fails to reproduce the experimental spin ladder for such systems (unless some *ad hoc* spin purification is performed), and thus the calculation of the magnetic coupling constant, and a more physical representation of the low-spin states requires a multireference treatment based on restricted-active-space SCF (RASSCF) or DMRG wave functions. However, also within the context of a large active space, a post wave-function treatment is needed. Multireference pair-density functional theory (MC-PDFT) starting from a large active space (DMRG or RASSCF active space with 30 electrons in 22 orbitals) gives encouraging results.^363^

#### 3.4.7 Kaupp and Arbuznikov

In the context of the failures for multireference cases, it seems important to mention attempts to account for strong-correlation effects and minimize fractional-spin errors.^26,27,364^ An important direction of development are Becke's real-space models of nondynamical correlation (initially the B05 functional^13^), which have been extended to account for strong-correlation terms by relying on the AC (Becke's B13 model^165^ and the related KP16/B13 model^169^ by Kong and Proynov). One interesting aspect of these functionals is that they are based on full exact exchange and model nondynamical correlation without using semilocal exchange.

Another important direction towards including strong-correlation effects also makes use of the AC but extends it to the strongly-correlated (*λ* → ∞) limit.^149,170,172^ While the question of how to best represent the noninteracting reference system of Kohn–Sham theory and the validity of the AC for multireference cases is still open, these approaches provide some hope of obtaining functionals that incorporate such effects.

#### 3.4.8 Romaniello

Kohn–Sham band structures are widely employed in solid-state physics. However, whereas this may be a reasonable approximation to the true charged excitation energies in the limit of weak correlation, it completely fails in the limit of strong correlation, as pointed out in contribution (3.4.2).

The paramagnetic phase of transition-metal oxides – which is systematically described as metallic, contrary to experiment – is a paradigmatic example. These systems are a challenge also for more advanced methods such as the *GW* method.^365^ However, this problem arises since we are modelling the paramagnetic phase as nonmagnetic. Trimarchi *et al.*^366^ have recently shown that band-structure theories can give a correct description of these systems provided that one models the spin-disordered paramagnetic phase using a larger supercell.

In fact, this is routinely done to model the spin-ordered antiferromagnetic phase: the nonmagnetic unit cell is doubled so that a different spin can be specified for the transition-metal atom. In this case, a band-gap opening is usually obtained in band-structure theories, reflecting the fact that, the more physical information is put into the problem, the less accurate a theory needs to be.

Maybe I can clarify this point with the simple example of the Hubbard dimer at one-half filling, which can give insight into a paramagnetic or an antiferromagnetic spin structure, depending on whether or not the spin symmetry is broken. In the atomic limit (where the electron–electron interaction dominates over the kinetic energy, hence we are in the regime of strong correlation) the two electrons, one with spin up and the other with spin down, are localized one on one site and the other on the other site with equal probability – that is, the ground state is the spin singlet 
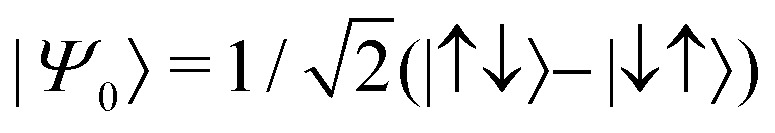
. The spectral function (which is related to photoemission spectra) thus shows, for each spin, two peaks with the same spectral weight 1/2 – one for the removal of an electron (peak at *ε*_0_, which is the orbital energy), and one for the addition of a second electron (peak at *ε*_0_ + *U*, with *U* the on-site electron–electron interaction), representing, respectively, the removal and addition energies, of an isolated atom with one electron.

Even the *GW* method cannot reproduce this spectrum. The *GW* method gives only one peak at *ε*_0_ + *U*/2, in line with the fact that this method describes many paramagnetic insulators as metallic. This happens because the *GW* method treats the charge/spin density as a classical charge distribution, with half an electron with spin up and half an electron with spin down on each atom that respond to the additional electron or hole added to the system in a photoemission experiment. If one considers instead the spin-symmetry broken state |*Ψ*_0_〉 = |↑↓〉 (or, equivalently, |↓↑〉), which is also an eigenstate of the system in this limit, then the *GW* method gives the correct spectral function. In this case, the electrons have fixed positions and one does not need to consider explicitly the correlation between two particles. One may therefore think that there is little correlation in this state. In reality, the system is correlated, but part of the correlation is included in the symmetry breaking.

#### 3.4.9 Galli

As indicated earlier, interfaces between metallic and insulating phases and interfaces between low-gap and wide-band gap semiconductors remain challenging to describe with existing DFAs – namely, band offsets of these interfaces are not accurate with most functionals and even structural properties in some cases^367^ turn out to be inaccurate. The electronic properties of several transition-metal oxides are equally challenging to describe with existing DFAs, especially those considered to be highly correlated materials. Empirical fixes have been proposed and used, as in the DFT+*U* method, but their predictive power is yet unclear, especially in cases where different values of *U* must be used for different oxidation states of the metal in the same oxide. An outstanding open problem is, for example, the metal-to-insulator transition in vanadium oxide (just to name one transition-metal oxide) as a function of oxygen composition or temperature.

### 3.5 What type or level of spatial nonlocality is required in explicit DFAs for the energy?

#### 3.5.1 Perdew

Given the exact electron density for a real system, and excluding exotic cases like the strongly stretched hydrogen molecule ion, the meta-GGA level of nonlocality can often give an accurate energy.^368^ But the level of nonlocality of the exchange–correlation potential (functional derivative of the exchange–correlation energy) can be much more critical for the electron density. The meta-GGA density is not sufficiently localized around the nuclear centres for some chemical problems, where the Hartree–Fock density, which comes from a more nonlocal exchange–correlation potential, is better.^118^ In a solid metal, however, the Hartree–Fock and even the meta-GGA density may be too localized in comparison with the exact density. Importantly, an approximate functional that is accurate for the energies on the exact electron densities of a wide class of real systems can still have inaccurate functional derivatives and thus inaccurate self-consistent densities in that class.

#### 3.5.2 Chermette

To generalize Perdew's statement in contribution (3.5.1), a given approximate functional can be very accurate for a given property, such as density, (everywhere or at nuclei), energy, or properties involving functional derivatives, but not for other ones. Indeed, this is just a consequence of the approximate nature of a DFA.

#### 3.5.3 Baerends

One consequence of the orbital levels for most DFAs being much too high (see contribution (3.2.13)) is a much too high-lying LUMO level. Adding an extra electron to the system then causes the LUMO level (containing the added electron) to be so close to zero that the LUMO becomes very diffuse, or even above zero, with an infinitely extended LUMO. This is a case where the Hartree–Fock method yields a much improved density.^369^ A similar effect can be achieved by using a more accurate model Kohn–Sham potential.^370,371^

On the other hand, while the Hartree–Fock density is typically very good for atoms, the Hartree–Fock model often yields poor bonding densities for molecules, being too diffuse around nuclear centres. For instance, for H_2_ it has been demonstrated that, due to this diffuse character, the errors in the one-electron energy terms (not sufficiently negative electron–nuclear energy and too low kinetic energy) are comparable to the error in the electron–electron energy.^69^ Upon stretching H_2_, the errors in the one-electron energy terms soon exceed the two-electron energy errors. For N_2_,^83^ the one-electron errors due to the too diffuse Hartree–Fock density are at the equilibrium distance already larger than the total bond energy of about 10 eV!

These errors can be understood from the limited flexibility of the Hartree–Fock wave function – that is, they arise from the lack of electron correlation. This gives a strong incentive to develop accurate model Kohn–Sham potentials that do better for the density and for the orbital energies and is also very important for the MO-theoretical explanations in chemistry.

#### 3.5.4 Xu

The electron density and the electronic energy are two quantities of fundamental importance. While an accurate description of the density allows for correct physical insight from the charge distribution, accurate determination of energies and their changes allows for precise quantification of the properties of a system of interest. The Hohenberg–Kohn theorems,^5,6^ which state that there exists a mapping from the ground-state electron density of a many-body system to its total energy, lay the foundation of modern DFT. To put DFT in practical use, the central questions are then (Q1) how to find the ground-state density of a physical system, and (Q2) how to set up a mapping from the density to the total energy. The Kohn–Sham scheme^65^ answers Q1 and Q2 simultaneously in a self-consistent way, using a local exchange–correlation potential, obtained by taking the derivative directly from a given DFA.

However, Q1 and Q2 can also be pursued separately.^372^ In cases where the Hartree–Fock method yields a much improved density (see contributions (3.5.1) and (3.5.3)), evaluation of the energy using a GGA functional on the Hartree–Fock density yields a much improved energy.^373–375^ It seems impractical, or even impossible, to demand that all properties be calculated accurately using a single, low-rung DFA; see contribution (3.5.2). It may eventually be possible for top-rung DFAs to give good densities, energies, and other properties, simultaneously.^332,376,377^ On the other hand, it is important to take both accuracy and efficiency into account. Hence, one can use a low-rung DFA to generate good orbitals and a good density efficiently, while using a top-rung functional to evaluate the energy accurately, as in the XYG3 double-hybrid functional.^134^

#### 3.5.5 Ayers

If one wishes to describe strong/static correlation using a Kohn–Sham DFA, then it is clear that enormous (even infinite) spatial nonlocality is required, because the (spherically-averaged) exchange–correlation hole can have a significant long-range structure. Moreover, when the multireference character is strong, that structure is exquisitely sensitive to small perturbations.

#### 3.5.6 Gill

Local DFAs are intrinsically incapable of capturing dispersion energy, which arises from long-range correlation effects between electrons.

#### 3.5.7 Grimme

The fact that semilocal DFAs yield an inconsistent or even unbound description of small van der Waals complexes was discovered in the mid-90s by Becke, Hobza, and Pulay.^378–380^ Noble gas dimers have been investigated several times as difficult cases for Kohn–Sham DFT, with large errors and sometimes qualitatively wrong behaviour being found. However, this “DFT failure” is actually a failure of the usual semi-local approximations and not of the theory itself. Around the same time, Meijer and Sprik presented an analysis of the problem for the typical case of the benzene dimer and noted related errors in the computed lattice energy or mass density of molecular crystals.^381^ General claims that semilocal DFAs cannot describe nonlocal long-range correlation (London forces) were occasionally made,^382^ but without further theoretical explanation – in particular, regarding the role of the correlation functional. Even as late as in 2002, the situation was not clear as indicated by a study of van Mourik and Gdanitz, which identified over-repulsive as well as over-binding functionals.^383^ For a more detailed historic account of the development of the dispersion problem in Kohn–Sham DFT, which cannot be solved simply by including nonlocal Fock exchange as is done in hybrid functionals, see ref. 384.

In those early days, the simple but incomplete picture prevailed that the dispersion energy is only relevant for the intermolecular situation – that is, for van der Waals complexes and condensed phases. The modern notion – namely, that intramolecular dispersion effects are especially important in large systems and in standard thermochemical applications – emerged only over the last ten years.^385^ Nowadays, newly proposed and accurate DFAs account for dispersion, which is mandatory for quantitative calculations and often even to obtain qualitatively correct results.

The most prominent dispersion correction schemes, which can be added to established DFAs, can be classified into the four groups:^384^ (i) nonlocal, density-based functionals (*e.g.*, vdW-DF or VV10), (ii) C_6_-based, atom-pairwise semiclassical models (*e.g.*, D3/D4, XDM, TS/MBD), (iii) one-electron effective potentials, and (iv) highly parameterized density functionals (*e.g.*, M06). Some of these methods, which mostly contain empirical components, yield very accurate long-range interactions, close to coupled-cluster accuracy (with a typical relative error of less than 5%) at low, often negligible computational cost.

Problems for particular systems or seemingly large differences between dispersion-corrected DFAs can often be attributed to an inaccurate description of short-range exchange–correlation effects, which are more difficult to describe than the long-range regime, dominated by 1/*R*^−6^ interactions. Dispersion effects can also be hidden by exaggerated charge-transfer interactions induced by the SIE in GGAs.^387^ Note further that, although London dispersion as a nonlocal correlation effect is omnipresent, it can be partly quenched in typical condensed-phase chemistry applications. In such systems, intramolecular noncovalent interactions compete with intermolecular solvent interactions and their subtle balance requires a sophisticated theoretical treatment of both dispersion and solvation.

#### 3.5.8 Tozer

The electrostatic theorem of Feynman (obtained by applying the differential Hellmann–Feynman theorem to a nuclear perturbation) states that the force on a nucleus equals the classical electrostatic force due to the electrons and nuclei in the system.^388^ This has great physical appeal since it relates the force on a nucleus directly to the electron density, in the true spirit of DFT.

The electrostatic theorem is formally exact, but breaks down for nonvariational methodologies and/or finite basis sets, meaning that it is of limited use in practice. However, for small systems where variational methodologies can be used with very large basis sets, the theorem is quantitatively applicable, meaning it provides an alternative perspective for viewing the “dispersion problem” of local functionals.^389^ Errors in dispersion forces can be understood in terms of errors in electron densities, which in turn can be understood in terms of errors in the exchange–correlation potential in the Kohn–Sham equations. Similar arguments can be applied to other problems, such as static correlation or delocalization errors.^390^

#### 3.5.9 Gori-Giorgi

The relevance of errors in electron densities for capturing dispersion interactions may need some reconsideration, or at least needs to be better understood, especially in the DFT setting. Pragmatically, poor densities can give very good dispersion energies – as an extreme example, it has been shown that it is possible to get exact dispersion energies between two one-electron systems up to and including orders *R*^−10^ without any deformation of the monomer densities.^391^

The subtle point with the electrostatic theorem of Feynman^388,392^ is that the result depends on whether one performs the derivative with respect to the nuclear position in the original coordinates or in the coordinates in which the electrons are centred on their respective nuclei.^393^ In the first case, the interaction energy depends only on the density distortion at order *R*^−7^ (for which the underlying wave function must be accurate to second order in the dipole–dipole and dipole–quadrupole interaction); in the second case, the interaction energy depends only on the distortion of the interfragment pair density at order *R*^−3^ (for which the underlying wave function must be accurate only to first order in the dipole–dipole interaction).^393,394^ These observations may suggest a route to build approximate exchange–correlation functionals by considering a simplified real-space mechanism, in which dispersion is reduced to the competition between kinetic energy and monomer–monomer interaction (thus keeping the density and pair density of the monomers unchanged, but producing an accurate interfragment pair density).^394^

#### 3.5.10 Dobson

Much relevant physics can be included in energy functionals *via* use of generalized “densities” assembled from Kohn–Sham orbitals, such as the positive local kinetic-energy density *τ*(**r**) used in meta-GGAs. Here, however, attention will be focused, as per the title question for this section, on strictly explicit functionals of the electron number density *ρ* and its space derivatives such as ∇*ρ* and ∇^2^*ρ*.

It may be useful to consider nonlocal functionals as a sum of “one-point”, “two-point”, “three-point”… contributions, where the *n*th term involves a 3*n*-dimensional space integral of a function *F*_*n*_ of the density and its derivatives, sampled at *n* different spatial points:26
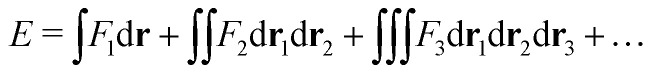
with

27 *F*_1_ ≡ *F*_1_(**r**, *ρ*(**r**), ∇*ρ*(**r**),…),

28 *F*_2_ ≡ *F*_2_(**r**_1_,*ρ*(**r**_1_),∇*ρ*(**r**_1_),…, *r*_2_,*ρ*(**r**_2_),∇*ρ*(**r**_2_),…),

and so on. Here, three dots … represent possibly a small finite number of additional space derivatives of *ρ*. Keeping an infinite number of derivatives would probably be equivalent to knowing the density everywhere *via* a three-dimensional Taylor series, at least for smooth densities. Then perhaps even the first term on the right-hand side of eqn (26) would represent the most general nonlocal density functional.

The LDA and GGA functionals correspond to the first term of the expansion in eqn (26). Examples of the second-order term (“two-point functionals”) are the naive Hartree energy and the vdW-DF energy functional of Langreth, Lundqvist, and co-workers.^395^

The expansion in eqn (26) may be relevant in the quest for explicit density functionals for the kinetic energy, a topic that has seen revived interest recently in the context of orbital-free DFT. Here, however, I will confine my remarks to the theory of van der Waals interactions (London dispersion),^396^ with which I am more familiar.

For dispersion interactions, the second-order term in eqn (26) has already had considerable success *via* the vdW-DF functional^395^ and its extensions.^397^ The third-order term would be needed, for example, for a strictly explicit density functional to capture the Axilrod–Teller–Muto interaction – that is, the van der Waals interaction between three atoms, taken beyond the summed interaction between pairs of atoms).

It has been known for some time that widely-spaced low-dimensional metals have van der Waals interactions that are qualitatively different from those between nonmetallic structures with a similar geometry;^398–400^ for some discussion, see Chapter 11 of ref. 396. Recently, it has been found that this specific metallic van der Waals physics is important beyond the asymptotic region, indeed right down to contact, for metallic nanotubes and doped graphene sheets. This behaviour was captured by calculations^401^ of the electron correlation energy in direct RPA (dRPA). Methods like dRPA start from an electronic band-structure calculation and are thereby sensitive to the presence or absence of a zero HOMO–LUMO gap (band gap). I fear that a very high order in the functional expansion in eqn (26) might be needed to capture such physics. I wonder, though, whether one could use a close examination of the ground-state electron density in the tunnelling region between atoms, in order to recognize the band gap. Certainly, this region determines the overlap (tunnelling) energy integral *t* in the tight-binding description of electronic band structure. In that case, perhaps the first few terms on the right-hand side of eqn (26) might be sufficient.

### 3.6 What is the role of symmetry breaking/restoration for DFAs?

#### 3.6.1 Perdew

Symmetry breaking reveals strong correlations that are present in a symmetry-preserving correlated wave function but “freeze out” in the total density or spin density of a Kohn–Sham DFT calculation.^402^ This often provides real information about the system being studied, and sometimes enhances the accuracy of the approximate functional. For example, when the bond length of the hydrogen molecule is strongly stretched, the symmetry-preserving ground-state wave function is a spin-unpolarized singlet state, whose energy most standard DFAs cannot get right, but the symmetry-broken solution reveals the correct dissociation to two separate hydrogen atoms, one spin up and the other spin down. In this way, symmetry breaking in approximate Kohn–Sham theory can capture what is a strong correlation in wave-function theory. What is strongly-correlated for one reference state can be weakly-correlated or even uncorrelated for another reference state. Kohn–Sham theory can also be re-interpreted as a theory not for the up- and down-spin densities but for the total density and on-top pair density.^403^

It is only *via* symmetry breaking that the interaction of the electrons with the nuclei can be regarded as a static external potential. In a symmetry-unbroken wave function for electrons and nuclei, all potentials are internal and all effects are correlations. The quantum theory of measurement requires a symmetry-broken or classical observer. The measured antiferromagnetism of solids is a physical symmetry breaking: a fluctuation or correlation that persists for a long time even on the human scale.^404^ Thus, condensed matter physicists tend to be more comfortable with symmetry breaking than many quantum chemists are. While the symmetry of the ground state of a finite system remains unbroken when averaged over an infinite time interval, the time interval over which the symmetry remains broken in a fluctuation can grow rapidly as the spatial extent of the system grows. The macroscopic world as we perceive it is symmetry-broken and classical.

#### 3.6.2 Gould

It is worth noting that, although symmetry breaking is extremely useful and often physically reasonable for the reasons mentioned in contribution (3.6.1), there are cases where preserving symmetries is important. A prime example is when we are interested in spectroscopic properties that are meant to be degenerate, but where the degeneracy is “spoiled” by symmetry breaking. Such cases can be dealt with by careful application of ensemble theories, as discussed in Section 3.7.

#### 3.6.3 Vignale

With reference to contribution (3.6.2), a good case in point is that of open-shell atoms, where rotational symmetry demands the existence of a degenerate multiplet of ground states when the magnitude of the orbital angular momentum *L* is nonzero. There is no functional that I know that can guarantee that this degeneracy is respected when the densities of the degenerate states are not trivially related to each other by a rotation. Years ago, Becke attempted to solve this problem by introducing a current-dependent functional, but could not achieve rigorous degeneracy.^405^

#### 3.6.4 Gould

It is worth noting that ensemble DFT, which invokes multiple Kohn–Sham states *via* ensemble density matrices, can restore all degeneracy. This is briefly discussed in Section 3.7.

#### 3.6.5 Savin

Spin-symmetry breaking is related to the general problem of degeneracy, as is the localization/delocalization problem.^173^ Note that the two-body density does not have the ensemble property used for the one-particle density. However, the real problem (not only for DFAs) is to deal with near degeneracy.

#### 3.6.6 Loos

In the DFT context, symmetry breaking might be seen as a signature of the approximate nature of a given exchange–correlation functional. Taking as an example the dissociation of the hydrogen molecule discussed in contribution (3.6.1), one might expect to never see any symmetry breaking if one employs the exact exchange–correlation functional within Kohn–Sham DFT, which is true for the Hubbard dimer.^406^ Thus, the ability of a given functional not to break the (spin and spatial) symmetries could be potentially seen as a diagnostic of its quality.

#### 3.6.7 Gould

While this is almost certainly true for exact spin-free DFT, there is an important consideration in Kohn–Sham DFT with spin densities. Even if we fix calculations to the exact total density, *ρ* = *ρ*_↑_ + *ρ*_↓_, the Kohn–Sham kinetic energy *T*_s_[*ρ*_↑_,*ρ*_↓_] can depend on *ζ* = |*ρ*_↑_ − *ρ*_↓_|/*ρ* (or, rather, on its Kohn–Sham equivalent, which may not be the same), and this dependence must be mirrored by *E*_xc_[*ρ*_↑_,*ρ*_↓_]. Since Kohn–Sham spin DFT seeks to minimize *T*_s_[*ρ*_↑_,*ρ*_↓_], it might be the case that *T*_s_[*ρ*_↑_,*ρ*_↓_] is minimized for a broken symmetry. It would be nice to determine if there is any exact symmetry breaking, or if spin DFT also preserves symmetries.

#### 3.6.8 Gori-Giorgi

Kohn–Sham DFT that uses the SCE limit (see contributions (2.4.5) and (4.5.8)) to approximate the exchange–correlation functional is able to stretch the H_2_ molecule without spin symmetry breaking.^172,407^ The SCE functional is also able to capture charge localization in very low-density systems without spatial symmetry breaking.^408^ However, the SCE functional is a very nonlocal approximation to the exchange–correlation functional (which is exact in a certain limit) and, at present, rather involved to evaluate. It also strongly overestimates correlation, so that a better strategy could be to design functionals that are inspired by the mathematical SCE structure but simplify and renormalize it.^145–147^

Apart from the reasons mentioned in contribution (3.6.2), further efforts to avoid symmetry breaking might be worthwhile in order to obtain potential-energy surfaces without kinks.

#### 3.6.9 Görling

To understand symmetry in Kohn–Sham DFT, it is necessary to look not only at the symmetry of the density or spin density but at the symmetry of the Hamiltonian operator of the true electronic system and of the Kohn–Sham Hamiltonian operator. The nonrelativistic Hamiltonian operator of the true electronic system is rotationally invariant in spin space even for a spin-polarized system with an odd number of electrons. Therefore spin is a good quantum number.

In the Kohn–Sham treatment, we then have a choice: (i) we can require the spin density to be identical in the Kohn–Sham and true electronic systems. This choice amounts to a spin-polarized Kohn–Sham calculation with a Kohn–Sham Hamiltonian operator that is no longer rotationally invariant in spin space. Spin is then no longer a good quantum number for the Kohn–Sham determinant – that is, spin poisoning occurs. (ii) Alternatively, we can require the total density but not the individual spin densities of the Kohn–Sham system to be identical to the true electronic system. The Kohn–Sham Hamiltonian operator then remains rotationally invariant in spin space and we get identical spin-up and -down orbitals. As a result, the orbitals and the Kohn–Sham wave function can be chosen to have well-defined spin but the individual Kohn–Sham spin-up and -down densities are no longer identical to those of the true electronic system. In practice, approach (i) is typically taken – however, approach (ii) is equally correct from a formal perspective, a point Walter Kohn made from time to time.

A similar choice can be made with respect to symmetries in real space. In open-shell atoms, for example, one can either require that the total density or the spin densities for the Kohn–Sham system and true electronic systems are identical or require that only their totally symmetric (*i.e.*, spherical) real-space components are identical.^409^ Depending on the choice made, the Kohn–Sham Hamiltonian either has a symmetry lower than the spherical symmetry of the true Hamiltonian in real space or is spherically symmetric in real space like the Hamiltonian of the true electronic system.

These choices, leading to different but formally correct Kohn–Sham approaches, must be distinguished from symmetry breaking of the type observed in a dissociating hydrogen molecule. The Kohn–Sham Hamiltonian has been shown to exhibit at least the symmetry of the total density or spin density of the true electronic system.^409^ In the dissociating hydrogen molecule, the true density is non-spin-polarized at all distances. Therefore, an exact spin-polarized Kohn–Sham calculation always reduces to the non-spin-polarized case. If this reduction does not occur, then it is an artefact of the employed approximate exchange–correlation functional, pointing to a shortcoming in the description of static correlation.

Finally, it should be pointed out that symmetry-breaking contributions in the Hamiltonian of the real system necessarily lead to corresponding terms in the Kohn–Sham Hamiltonian. Spin–orbit interactions, for example, require from a formal point of view the presence of terms in the Kohn–Sham potentials that couple to spin or magnetization currents.^410^ In practice, these terms are often neglected. It is interesting to note that, from a formal point of view, terms in the Kohn–Sham potential that couple to noncollinear spin are not required in the presence of spin–orbit interactions.^410^

#### 3.6.10 Chermette

Taking approach (ii) of contribution (3.6.9), a powerful, although limited, method of use for spectroscopy is ligand-field DFT (LFDFT) developed by Daul *et al.*^411,412^ This semiempirical method uses all the symmetry constraints included in the ligand-field formalism. Its parameters are extracted from a standard (usually restricted GGA) Kohn–Sham calculation. The spherical symmetry of the atomic densities is obtained by fractional occupation of the involved orbitals.

For instance, for a f^7^ → d^1^ transition in a lanthanide compound (here europium), the GGA Kohn–Sham MO occupations corresponding to the 4f^6^d^1^ configuration is 6/7e for the (7) MOs strongly localized on the Eu/4f orbital, and 1/5e for the (5) MOs mostly localized on the Eu/5d orbital. See, for example, the case of the Eu(η_9_-C_9_H_9_)_2_ complex, for which the 30 030 multiplet energy levels have been calculated.^413^

#### 3.6.11 Krylov

To properly deal with symmetry breaking, we need to look at properties that are rigorously defined – for example, it is difficult to discuss what spin symmetry means within DFT because *S*^2^ is a two-electron operator.^414^

So spin-contaminated (as traditionally computed) Kohn–Sham DFT or TDDFT solutions might, in fact represent the correct spin densities of the spin-pure correlated many body wave functions – for example, an open-shell doublet radical (such as CH_3_) in which the unpaired electron has alpha spin, is known to have areas with an excess beta-spin density.^414^ This cannot be reproduced by a spin-adapted (ROHF) Kohn–Sham determinant (which only allows for an excess alpha density), hence suggesting that a spin-polarized Kohn–Sham determinant provides a more appropriate description.

Because we do not know how to compute the *S*^2^ value in DFT, we should formulate this question – whether or not we have unphysical symmetry breaking – in terms of finding molecular properties that could report on it. The same concerns apply to spatial symmetry breaking. One example of how one may approach this problem is a charge-transfer system, such as (He)_3_^+^ or the charged ethylene dimer. Charge localization is very sensitive to Hartree–Fock-like symmetry breaking and also to the SIE. The comparison of charge localization patterns against high-level reference data can inform us whether symmetry breaking is real or artificial; some useful examples can be found in ref. 415.

#### 3.6.12 Baerends

In contribution (3.6.6), the “challenge” is put forward that, with the dissociating H_2_ molecule as an example, one would never see any symmetry breaking if one employs the exact exchange–correlation functional within Kohn–Sham DFT. Actually, the dissociating H_2_ molecule is a simple enough system that such a functional can be formulated.^416^ This functional becomes exact in the dissociation limit and does not lead to any symmetry breaking. Not unexpectedly, it is orbital dependent and employs in addition to the σ_g_ orbital the σ_u_ orbital. Since such involvement of “unoccupied” orbitals can be regulated *via* the natural orbital occupations, this ushers in reduced-density-matrix-functional theory. For heavier systems, such functionals become approximate, but can still provide good dissociation curves without symmetry breaking.^417^ In the Kohn–Sham context, the temperature-assisted occupation DFT (TAO-DFT) of Chai^418^ is an example of attempts to involve virtual Kohn–Sham orbitals *via* 1RDM-like occupation schemes.

### 3.7 What is the role of ensemble methods for DFAs?

#### 3.7.1 Savin

When discussing ensembles, we should be careful to distinguish between the different cases. Are we interested in describing ensembles associated with degenerate states? For example, do we want to construct universal density functionals that have the same value for all the densities of the ensemble? Do we mean errors that show up due to the locality in our approximations^419^ – for example, at dissociation? Do we mean ensembles that show up in (even accurate) Kohn–Sham calculations (*cf.* the pure-state *v*-representability discussion in ref. 7)? Do these ensembles survive at weak interactions? If a multireference treatment is needed in wave-function theory, can we use ensembles with (semi)local DFAs in DFT? We should keep in mind that the same classical ensemble may correspond to different wave functions, by ignoring the effect of the interference term produced by the sign (phase) of the coefficients. Ensembles are introduced for very different reasons for ground states, excited states and high temperature.

#### 3.7.2 Gould

Ensemble DFT^420^ extends the Kohn–Sham method and related theorems to a much wider range of problems – everything including thermal states,^50^ degenerate states,^6,8^ “partial” electrons,^18^ excited states,^421,422^ and states that give direct access to fundamental gaps.^423^ DFAs based on ensemble principles should be able to inherit this generality and thereby offer insights into systems that cannot be described by standard DFAs.

Thermal ensembles are rather different to the other types of ensembles. The following discussion thus focuses on other types of ensembles, which give insight into spectroscopic properties of electronic systems, like fundamental and optical gaps.

Despite representational issues in some systems, there are a wide variety of problems for which ensembles can be described cleanly, and for which mappings are one-to-one.^424^ In such cases, key ensemble functionals may be defined as,^6,8^29
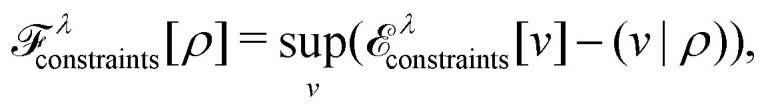
30
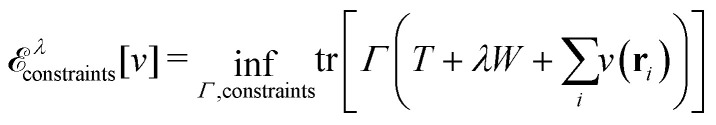
where *T* and *W* are the kinetic-energy and two-electron repulsion operators, respectively. Here, the energy is found by a constrained minimization over density matrices *Γ*, with different constraints leading to different types of ensemble theories. One may then define an ensemble Kohn–Sham theory, using,

31

<svg xmlns="http://www.w3.org/2000/svg" version="1.0" width="23.636364pt" height="16.000000pt" viewBox="0 0 23.636364 16.000000" preserveAspectRatio="xMidYMid meet"><metadata>
Created by potrace 1.16, written by Peter Selinger 2001-2019
</metadata><g transform="translate(1.000000,15.000000) scale(0.015909,-0.015909)" fill="currentColor" stroke="none"><path d="M560 840 l0 -40 -80 0 -80 0 0 -40 0 -40 -40 0 -40 0 0 -40 0 -40 -40 0 -40 0 0 -80 0 -80 40 0 40 0 0 -40 0 -40 80 0 80 0 0 40 0 40 40 0 40 0 0 40 0 40 40 0 40 0 0 40 0 40 40 0 40 0 0 80 0 80 80 0 80 0 0 -40 0 -40 -40 0 -40 0 0 -80 0 -80 -40 0 -40 0 0 -40 0 -40 -40 0 -40 0 0 -80 0 -80 -40 0 -40 0 0 -40 0 -40 -40 0 -40 0 0 -40 0 -40 -40 0 -40 0 0 -40 0 -40 -80 0 -80 0 0 80 0 80 -40 0 -40 0 0 -80 0 -80 40 0 40 0 0 -40 0 -40 120 0 120 0 0 40 0 40 40 0 40 0 0 40 0 40 40 0 40 0 0 40 0 40 40 0 40 0 0 80 0 80 40 0 40 0 0 40 0 40 40 0 40 0 0 80 0 80 40 0 40 0 0 80 0 80 120 0 120 0 0 40 0 40 -320 0 -320 0 0 -40z m80 -120 l0 -80 -40 0 -40 0 0 -40 0 -40 -40 0 -40 0 0 -40 0 -40 -80 0 -80 0 0 80 0 80 40 0 40 0 0 40 0 40 80 0 80 0 0 40 0 40 40 0 40 0 0 -80z"/></g></svg>

_s_[*ρ*] := 

<svg xmlns="http://www.w3.org/2000/svg" version="1.0" width="22.363636pt" height="16.000000pt" viewBox="0 0 22.363636 16.000000" preserveAspectRatio="xMidYMid meet"><metadata>
Created by potrace 1.16, written by Peter Selinger 2001-2019
</metadata><g transform="translate(1.000000,15.000000) scale(0.015909,-0.015909)" fill="currentColor" stroke="none"><path d="M480 840 l0 -40 -40 0 -40 0 0 -40 0 -40 -40 0 -40 0 0 -40 0 -40 -40 0 -40 0 0 -80 0 -80 40 0 40 0 0 -40 0 -40 40 0 40 0 0 40 0 40 40 0 40 0 0 40 0 40 40 0 40 0 0 40 0 40 40 0 40 0 0 40 0 40 -40 0 -40 0 0 -40 0 -40 -40 0 -40 0 0 -40 0 -40 -40 0 -40 0 0 -40 0 -40 -40 0 -40 0 0 80 0 80 40 0 40 0 0 40 0 40 40 0 40 0 0 40 0 40 160 0 160 0 0 -40 0 -40 -40 0 -40 0 0 -80 0 -80 -40 0 -40 0 0 -40 0 -40 -40 0 -40 0 0 -40 0 -40 -40 0 -40 0 0 -120 0 -120 -80 0 -80 0 0 -40 0 -40 -80 0 -80 0 0 40 0 40 40 0 40 0 0 40 0 40 -80 0 -80 0 0 -80 0 -80 40 0 40 0 0 -40 0 -40 120 0 120 0 0 40 0 40 80 0 80 0 0 80 0 80 40 0 40 0 0 40 0 40 80 0 80 0 0 40 0 40 80 0 80 0 0 40 0 40 40 0 40 0 0 40 0 40 -80 0 -80 0 0 -40 0 -40 -40 0 -40 0 0 120 0 120 40 0 40 0 0 40 0 40 160 0 160 0 0 40 0 40 -360 0 -360 0 0 -40z"/></g></svg>

^0^[*ρ*], 

<svg xmlns="http://www.w3.org/2000/svg" version="1.0" width="17.166667pt" height="16.000000pt" viewBox="0 0 17.166667 16.000000" preserveAspectRatio="xMidYMid meet"><metadata>
Created by potrace 1.16, written by Peter Selinger 2001-2019
</metadata><g transform="translate(1.000000,15.000000) scale(0.014583,-0.014583)" fill="currentColor" stroke="none"><path d="M560 920 l0 -40 -40 0 -40 0 0 -40 0 -40 -40 0 -40 0 0 -80 0 -80 40 0 40 0 0 -40 0 -40 -40 0 -40 0 0 -40 0 -40 -80 0 -80 0 0 -40 0 -40 -80 0 -80 0 0 -120 0 -120 40 0 40 0 0 -40 0 -40 40 0 40 0 0 -40 0 -40 200 0 200 0 0 80 0 80 40 0 40 0 0 40 0 40 40 0 40 0 0 80 0 80 -40 0 -40 0 0 40 0 40 -40 0 -40 0 0 -40 0 -40 -40 0 -40 0 0 -40 0 -40 -40 0 -40 0 0 -40 0 -40 40 0 40 0 0 40 0 40 40 0 40 0 0 40 0 40 40 0 40 0 0 -80 0 -80 -40 0 -40 0 0 -40 0 -40 -40 0 -40 0 0 -40 0 -40 -160 0 -160 0 0 120 0 120 40 0 40 0 0 40 0 40 40 0 40 0 0 40 0 40 80 0 80 0 0 160 0 160 40 0 40 0 0 40 0 40 120 0 120 0 0 -80 0 -80 -40 0 -40 0 0 40 0 40 -40 0 -40 0 0 -40 0 -40 40 0 40 0 0 -40 0 -40 40 0 40 0 0 40 0 40 40 0 40 0 0 80 0 80 -40 0 -40 0 0 40 0 40 -160 0 -160 0 0 -40z"/></g></svg>

_Hxc_[*ρ*] := ^1^[*ρ*] − ^0^[*ρ*],

where _s_ and _Hxc_ serve the same role as in conventional DFT. “Constraints” are henceforth implied by the use of calligraphic letters.

Eqn (31) defines an ensemble Kohn–Sham system with orbitals obeying,32
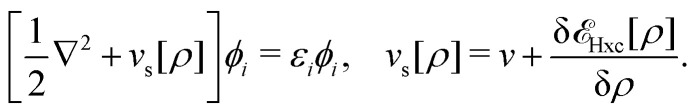
The density, 
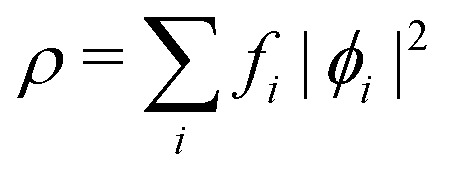
, is defined using orbitals with allowed fractional occupation factors, *f*_*i*_, that reflect the nature of the ensemble. Ensembles are thus amenable the same machinery as standard DFT – that is, by approximating ℰ_Hxc_[*ρ*] and then finding a set of self-consistent orbitals and density. Accommodating ensembles in Kohn–Sham DFT not only extends approximations to new problems (like excited states), but can also remedy deficiencies in standard approximations.^425,426^

A major difficulty in treating ensembles is that the minimizing wave functions and ensembles are not guaranteed to be unique and cannot be used explicitly to define functionals – the “nonuniqueness disaster”.^427^ This is related to issues raised in contribution (3.7.1). One must thus resort to more foundational relationships to further break ℰ_Hxc_ into useful pieces that may then be approximated. A rigorous separation into Hartree–exchange and correlation terms is achieved by using,33
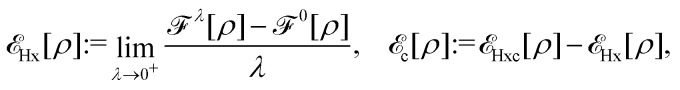
which gives the usual results for pure states. In the special case of ensembles that preserve fundamental symmetries by equally weighting states related by symmetry operations,^424^ one may also rigorously define the Hartree term ℰ^FDT^_H_[*ρ*] and exchange term ℰ^FDT^_x_[*ρ*] using the fluctuation-dissipation theorem.^428^ The resulting orbital functionals reduce to their usual definitions in pure states. Hybrid functionals formed on these orbital functionals (*e.g.*, by using 𝒯_s_ + ℰ^FDT^_H_ + *α*ℰ^FDT^_x_, where *α* is a HF mixing parameter) may be defined using ensemble generalized Kohn–Sham theory.^175^

An additional challenge in ensemble DFT is that the correlation energy is more complicated than its standard (pure-state) DFT counterpart. Firstly, because it must address multiple states at once. Secondly, because it contains density-driven (DD) correlations,^429^ which are a consequence of the fact that Kohn–Sham states reproduce the correct total density, but not the correct densities of the individual interacting states included in the ensemble. Gould and Pittalis defined DD correlations in special types of ensembles.^429^ Fromager then provided a rigorous general scheme for understanding DD correlations.^430^

#### 3.7.3 Fromager

Just for the sake of clarity, it is probably good to explain why a distinction has to be made between thermal ensembles and other types of ensembles like, for example, the Gross–Oliveira–Kohn (GOK) ensemble^422^ or the more recent *N*-centred ensembles,^423^ which are (somehow artificially) constructed in order to compute neutral or charged excitation energies in a completely time-independent formalism.

The discussion that follows focuses on the latter type of ensembles, which we could refer to as “pre-defined” ensembles. Indeed, unlike in thermal DFT,^431,432^ the ensemble weights **ξ** = (*ξ*_0_,*ξ*_1_,…, *ξ*_*I*_,…) that are assigned to each state within the ensemble will always be known before the ensemble DFT calculation is carried out. They are chosen (in principle, arbitrarily) and fixed. In other words, in the exact theory, the Hohenberg–Kohn theorem is established for a given ensemble or, equivalently, for a given set **ξ** of weight values. The one-to-one correspondence between local potentials and ensemble densities relies on the extension (from pure ground states to ensembles) of the Rayleigh–Ritz variational principle,^421,423^34
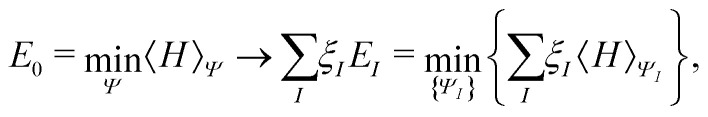
where {*E*_*I*_} are the targeted (ground- and excited-state) energies. The ensemble Hartree-exchange–correlation (Hxc) energy functional

35 ℰ_Hxc_[*ρ*] ≡ *E*^***ξ***^_Hxc_[*ρ*]

is said to be universal because it does not depend on the external (local) potential. However, it is expected to depend on the ensemble under study, through its weight dependence. The latter originates from the fact that a density *ρ* that integrates to a fixed integer number *N* of electrons can be both pure-state and ensemble *N*-representable at the same time:^423,433^36
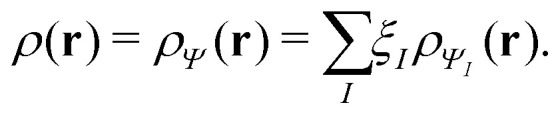
A simple example is provided by the hydrogen atom. The effective 1s orbital37
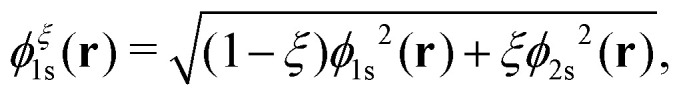
which represents the ground state of −∇_**r**_^2^/2 + *v*^*ξ*^(**r**) in the potential *v*^*ξ*^(**r**) = ∇_**r**_^2^[*ϕ*^*ξ*^_1s_(**r**)]/(2*ϕ*^*ξ*^_1s_(**r**)), has the same density as the GOK ensemble constructed from the regular 1s and 2s orbitals with weights (1 − *ξ*) and *ξ*, respectively.

In the general many-electron case, the ensemble Hxc functional needs to know if it has to compute the Hxc energy of a pure ground state or of an ensemble consisting of ground and excited states, hence the ***ξ*** dependence in *E*^***ξ***^_Hxc_ [*ρ*]. The extraction of excitation energies from an ensemble DFT calculation reveals the importance of this weight dependence.^422,423,434,435^ In particular, it has been shown that ensemble density-functional weight derivatives ∂*E*^***ξ***^_Hxc_[*ρ*]/∂*ξ*_*I*_ are directly connected to the derivative discontinuities that the Hxc potential exhibits when a given excited state is incorporated into the ensemble.^436–438^

In DFT for (canonical, for simplicity) thermal ensembles,^431,432,439^ the ensemble weights are controlled by a single parameter – the temperature or, equivalently, the inverse temperature *β*. In thermal DFT, the variational principle is extended to the total (Helmholtz) energy, which contains an entropic contribution:38
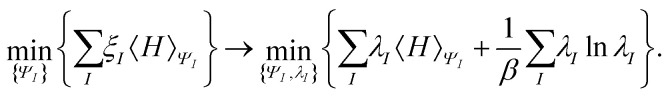
Note that, unlike in GOK or *N*-centred ensemble DFT, the minimizing ensemble weights
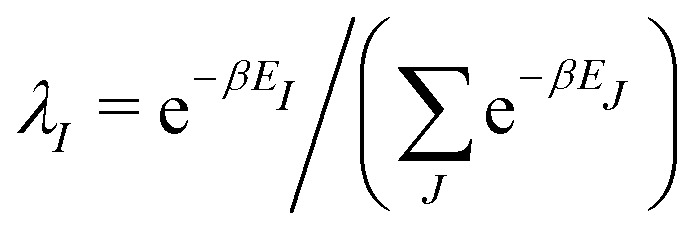
are energy dependent. Therefore, they are unknown when the calculation starts. Moreover, even though both interacting and noninteracting Kohn–Sham systems are described at the same temperature and share the same (ensemble) thermal density, their ensemble weights are different, simply because Kohn–Sham energies do not match the true interacting ones.

With these major differences in mind, the discussion on ensemble DFAs that follows essentially applies also to thermal DFT. The ensemble-weight dependence of the Hxc functional simply reduces to a temperature dependence.

#### 3.7.4 Gould

The last few years have seen significant development of new ensemble DFAs – especially for excited states. There are two main approaches: (i) explicit functionals of the density and ensemble weights (*i.e.*, constraints); (ii) ensemble-adaptation of existing functionals.

#### 3.7.5 Loos

Concerning point (i) of contribution (3.7.4), different strategies have been followed. In ref. 440, Loos and Fromager constructed a weight-dependent LDA (correlation) functional for GOK DFT^422^ using both finite and infinite uniform electron gases. This functional was employed to compute single and double excitations in one-dimensional systems. In ref. 441, Marut *et al.* designed, in the spirit of optimally-tuned range-separated hybrid functionals, a two-step system-dependent procedure (resulting in the construction of a weight-dependent exchange functional) to obtain accurate double excitations for two-electron atomic and molecular systems. The transferability of these weight-dependent functionals remains questionable.

#### 3.7.6 Gould

On point (ii) of contribution (3.7.4), the ability to rigorously define ℰ_Hx_^427^ and then break it down into ℰ^FDT^_H_ and ℰ^FDT^_x_ ^428^ has offered insights into adapting existing approximations to ensembles – because the exact-exchange functional of more complex excitations can obey combination rules that relate it to simpler pure-state systems for which approximations already exist. I showed that using exact-exchange relationships for ensembles allowed ensemble DFT to outperform the ΔSCF and TDDFT methods using the same DFAs.^442^ This success has been partially transferred to double excitations.^443^ Despite improvements from using the ensemble version of the on-top pair density *Π*(**r**,**r**) given for pure states in eqn (25),^443^ how to effectively reuse existing correlation DFAs remains an outstanding problem. It should be noted, however, that these DFAs require solution of orbital equations.^175^

Note that the approaches discussed above focus on modelling the Hx functional and state-driven correlations.^429,430^ Failure to include DD correlations in ensemble DFAs leads to “DD correlation errors” that are avoided in pure-state DFT. Gould introduced an extrapolation scheme to approximately avoid DD correlation errors.^424^

#### 3.7.7 Loos

Several current limitations of ensemble DFT are worth mentioning here:

(1) Self-consistent ensemble DFT calculations still lack a well-defined computational protocol (usual or generalized Kohn–Sham schemes, OEP-type algorithms, CASSCF-type orbital optimization techniques, *etc.*). How best to correct the ghost-interaction error at an affordable cost is also an open question.^444^

(2) In GOK DFT,^421^ one is supposed to know in advance the energy ordering of the excited states, which is far from being straightforward.

(3) Different flavours of ensemble DFT are used depending on the type of excitations targeted. In this context, a unified theory for charge and neutral excitations would be desirable in order to be able to compute both the fundamental and optical gaps from a single calculation.

#### 3.7.8 Fromager

I would like to complement the discussion in contributions (3.7.4), (3.7.6) and (3.7.7) from the perspective of state-averaged (usually multiconfigurational) wave-function-based methods.^445^ The incorporation of ensemble-weight dependencies into DFAs is probably the most challenging task in ensemble DFT. Defining Hartree, exchange, and correlation ensemble energies is not as straightforward as in regular ground-state DFT. Various decompositions have actually been proposed.^427–430,438^ They all have their advantages and drawbacks. A dilemma already appears at the Hartree-only level of approximation. In the original formulation of GOK^422^ and *N*-centred^423^ ensemble DFTs, the ensemble Hartree energy is evaluated from the regular ground-state Hartree density functional39
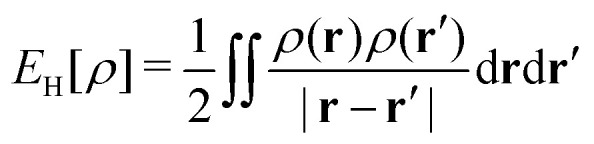
as follows:40
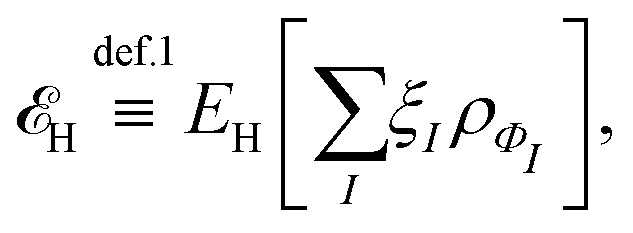
where {*Φ*_*I*_} are trial Kohn–Sham wave functions. While the above definition is formally convenient because it ensures that the Kohn–Sham orbitals are obtained from a single (local) ensemble-density-functional potential (Hartree-only in this case), it is, from a practical point of view, a very poor choice. The reason is that it contains unphysical “ghost” interaction terms between the states.^444^ The Hartree energy defined in this way also varies quadratically with the ensemble weights, by construction, while the exact ensemble energy varies linearly. At first sight, it seems better to opt for the following definition,41
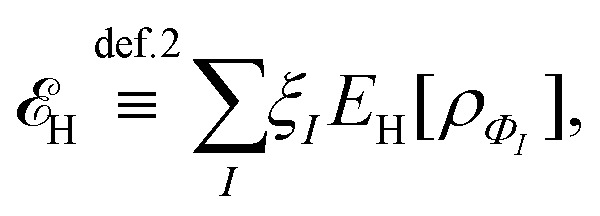
where individual Hartree energies are used instead. The above ensemble Hartree energy is an implicit functional of the ensemble density. If we want to preserve the original formulation of ensemble DFT, where a single local ensemble Kohn–Sham potential is employed, OEP techniques must be employed.^175,446^

Nevertheless, it is possible to tackle the problem differently. Indeed, an orbital-dependent Hartree-only density functional can be defined using Lieb's maximization (see eqn (29)) and the following approximate expression for the potential-functional ensemble energy:^438^42
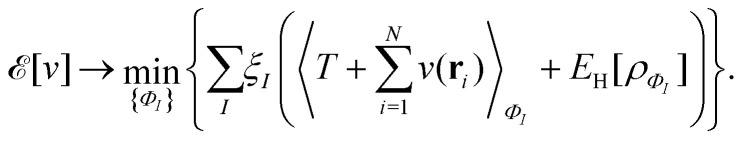
This procedure can be seen as the Hartree-only version for single-configuration (Kohn–Sham) wave functions of the state-averaged CASSCF (SA-CASSCF) method.^445^ Its practical disadvantage is that standard SCF routines cannot be used in this context. Indeed, as each Kohn–Sham state generates its own Hartree potential, there is no single ensemble potential from which the minimizing Kohn–Sham orbitals can be determined (by diagonalization).^175,438,447,448^ If we want to avoid the use of OEPs, this is essentially the price to pay for constructing ghost-interaction-free ensemble energies in a systematic and general way. Mapping the true interacting ensemble density onto such an approximate Hartree-only state-averaged ensemble leads to an alternative (in principle, exact) formulation of ensemble DFT.^438^ An exact ensemble exchange scheme is obtained along the same lines from the following approximate ensemble energy expression (note that, in practice, complementary fractions of exact and approximate density-functional expressions for the ensemble exchange energy are usually combined^438,448^):43
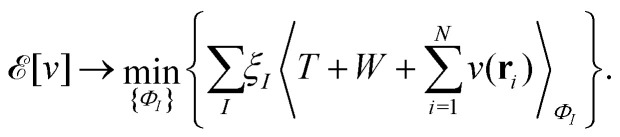
In this case, the individual (nonlocal) exchange potentials are functionals of the individual one-electron reduced density matrices.

Electron correlation can be introduced (approximately) into the theory by recycling the regular (weight-independent) ground-state correlation functional *E*_c_[*ρ*] as follows:44
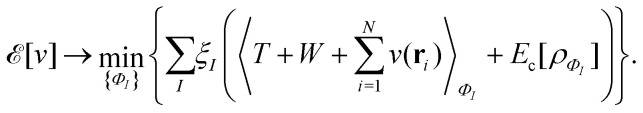
Mapping the true ensemble density onto such an (approximate) ensemble leads to another exact formulation of ensemble DFT. In order to recover the exact ensemble energy, a density-functional correction should then in principle be designed,45
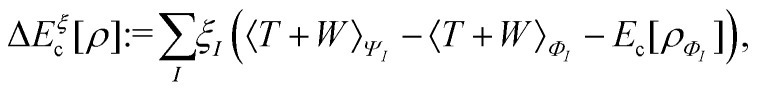
where {*Ψ*_*I*_} and {*Φ*_*I*_} are, respectively, the true interacting and auxiliary (generalized Kohn–Sham) density-functional ensembles with density46
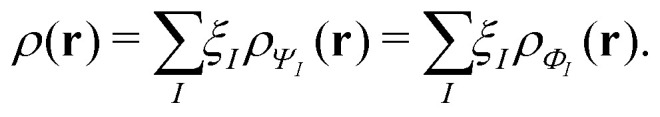
This is perhaps where the challenge in ensemble DFT lies – indeed, in computational studies, Δ*E*^***ξ***^_c_[*ρ*] is usually neglected.^448^ It is far from clear how accurate such an approximation is and if error cancellations systematically occur in this context; hence, the (urgent) need for a clearer hierarchy of approximations – that is, a Jacob's ladder for ensembles.

Let us give further insight into the approximation of eqn (44). From the more explicit expression47
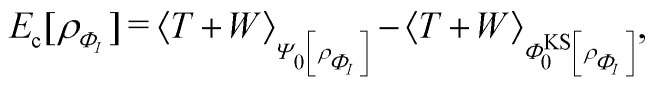
where *Ψ*_0_[*ρ*] and *Φ*^KS^_0_[*ρ*] are the standard interacting and Kohn–Sham noninteracting density-functional ground-state wave functions, respectively, we can rewrite the exact ensemble density-functional correlation correction to eqn (44) as follows:48
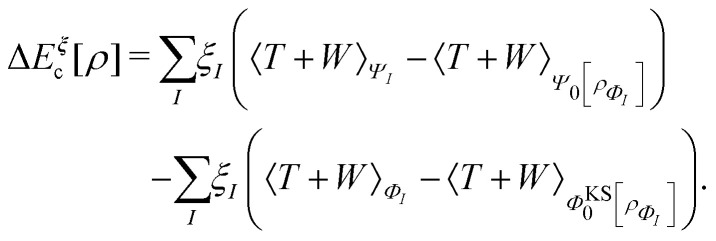
At this point, we stress that the density constraint of eqn (46) does not imply that, within the ensemble, interacting and Kohn–Sham densities match individually – in general, they do not.^429,430^ This can be seen in the regular ground-state limit of the theory, when the weights assigned to the excited states are equal to zero.^438^ This specific feature of ensemble DFT is reflected in the implicit weight-dependence of the Kohn–Sham wave functions {*Φ*_*I*_}.^430^ It is related to the concept of density-driven (DD) correlation recently introduced by Gould and Pittalis;^429^ see contribution (3.7.2). Moreover, even if the exact individual densities *ρ*_*Ψ*_*I*__ (which can be extracted, in principle exactly, from the Kohn–Sham ensemble^430^) were used instead of the bare Kohn–Sham densities *ρ*_*Φ*_*I*__, one would still not recover the exact ensemble correlation energy simply because, for a given excited-state density *ρ*_*Ψ*_*I*__, *Ψ*_0_[*ρ*_*Ψ*_*I*__] is always a ground-state wave function. The fact that the true excited-state wave function *Ψ*_*I*_ differs from *Ψ*_0_[*ρ*_*Ψ*_*I*__] can be related to the concept of state-driven (SD) correlation.^429^ In the light of this analysis, the following decomposition may be used as a guideline for the development of ensemble correlation DFAs:49
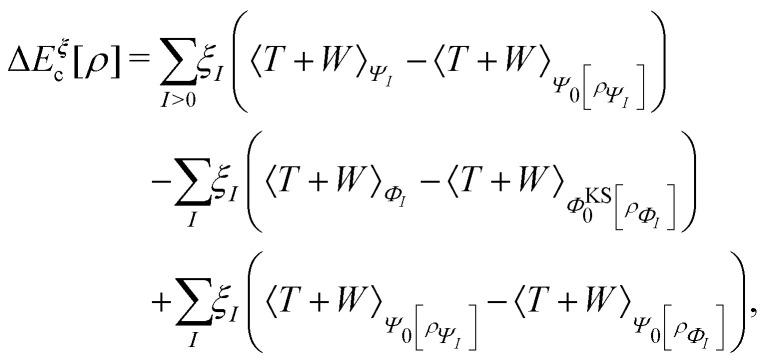
where the ground-state (*I* = 0) interacting contributions in the first summation rigorously cancel out.

Let us finally mention that ensemble DFT does not give a direct access to response properties such as oscillator strengths or to Dyson orbitals (in the case of *N*-centred ensemble DFT^423^). The extension of Görling–Levy perturbation theory^133,449,450^ to ensembles should probably be explored for that purpose.

#### 3.7.9 Grimme

Finite-(electronic)-temperature Kohn–Sham DFT, even with standard GGA or hybrid DFAs, can be used routinely to approximately describe difficult static-correlation problems in large systems even if the resulting energies need to be taken with caution. Such calculations often improve SCF convergence and can be employed, for example, in an MD treatment of high-energy chemistry (mass spectrometry^451^) or for the analysis of static-correlation effects.^452^

#### 3.7.10 Chermette

Most of the ensemble approaches discussed here are for “true” ground states or excited states, which naturally involve an integer (total) number of electrons. However, reactions or excitation processes may be described with a noninteger number of electrons, which mimics the approach of a charged or simply polarized species or particles. The relaxation of the MOs that occurs through the addition or depletion of a small amount of an electron is a tool not (yet) widely used. Organometallic complexes are sensitive to this because of their partially filled d orbitals. As a result, static (quasi-degeneracy) correlation may play a significant role – see, for instance, ref. 453.

#### 3.7.11 Gao

I would like to add an alternative formulation of the density functional of an ensemble of states in multistate DFT (MSDFT).^454,455^ We have been experimenting with this approach in the past few years, with excellent results in a variety of applications – including applications to singlet fission,^456^ proton-coupled electron transfer,^455^ conical intersections,^454^ local-valence and charge-transfer excited states,^457^ and core-level excitation energies.^458^

Recently, my coworker Dr Yangyi Lu and I proved that MSDFT is an exact DFT in the subspace 𝕍^*N*^ spanned by the lowest *N* eigenstates of the Hamiltonian.^459^ It is proved (1) that the Hamiltonian projected onto 𝕍^*N*^ is a matrix functional *H*[*D*(**r**)] of the multistate matrix density *D*(**r**) and (2) that variational minimization of the multistate energy, *E*_MS_[*D*] = tr_V_[*S*^−1^*H*(*D*)], gives the exact energies and densities of all *N* eigenstates. The second theorem corresponds to an extension of the Theophilou variational principle for the whole subspace^460^ in terms of *H*[*D*(**r**)], ensuring that the energies and vectors of individual states are obtained simultaneously. In these expressions, *D*(**r**) is a matrix of electron densities and transition densities of a set of basis states that represent the ensemble density *ρ*_V_(**r**) = tr_V_[*S*^−1^*D*(**r**)] of the subspace 𝕍^*N*^, where *S* is the overlap matrix of the basis states; *D*(**r**) is not to be confused with the one-electron density matrix.

The multistate matrix density *D*(**r**) can be sufficiently represented by *N*^2^ nonorthogonal (necessary) determinants.^459^ In a departure from Kohn–Sham DFT for the ground state, we introduce in MSDFT an “active space” of *N* interacting states {*Φ*_*A*_; *A* = 1,…, *N*}, each of which is written as a linear combination of *N*^2^ nonorthogonal determinants, 
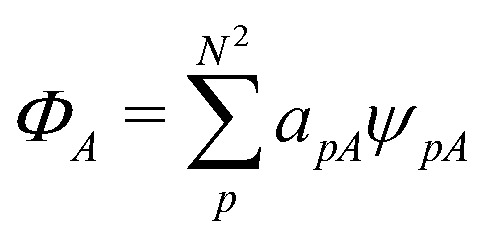
, to completely represent *D*(**r**) of the real (fully interacting) system. Its matrix elements are computed from one-electron orbitals {*χ*^*Ap*^_*j*_(**r**)} in the manner50
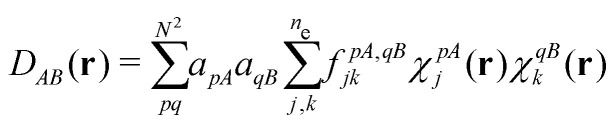
where *f*^*pA,qB*^_*jk*_ is the overlap between two Slater determinants of the corresponding (*n*_e_ − 1) orbitals. Unlike the diagonal state densities, the transition density *D*_*AB*_(**r**) with *A* ≠ *B* can be positive, negative or complex.

Analogous to the ground-state DFT, we can define a Lieb-like subspace energy functional51
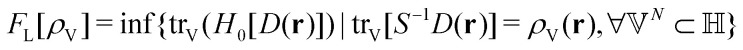
where *H*_0_ = *T* + *W* = *H* − *v*(**r**). The constrained minimization in eqn (51) imposes the condition that the total density of the *N* eigenstates of primary interest is identical to the subspace density. The optimal subspace density *ρ*_V_(**r**) is found by minimizing the multistate energy functional, an implicit functional of *ρ*_V_(**r**), with respect to *D*(**r**),52
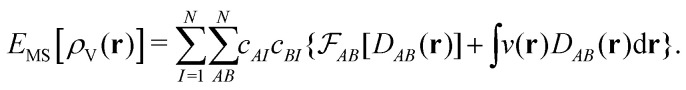
The energies of all *N* eigenstates of *H* within the subspace 𝕍^*N*^ are thus simultaneously determined.53
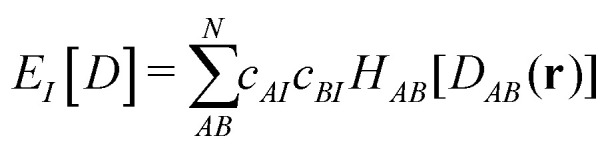
where *I* = 1,…, *N* and *E*_1_ is the ground state energy. The matrix functional of the full Hamiltonian *H* in the subspace 𝕍^*N*^ is given by54
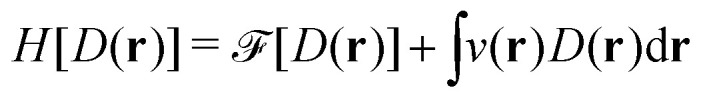
where ℱ[*D*(**r**)] is the universal matrix functional, whose elements in terms of one-body orbitals are

55 ℱ[*D*(**r**)] = *T*_MS_[*D*(**r**)] + *E*_Hx_[*D*(**r**)] + *E*_c_[*D*(**r**)]

The first and second terms in eqn (55) are, respectively, the multistate (active space) matrix functionals of the kinetic energy and of the Hartree–exchange energy:56
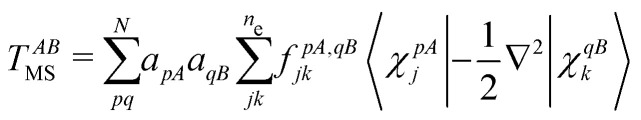
57
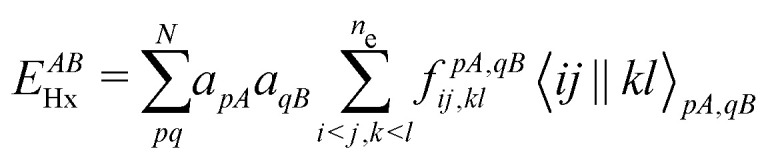
where 〈*ij*||*kl*〉_*Ap*,*Bq*_ is the two-electron Coulomb-exchange integral with *f*^*Ap,Bq*^_*ij,kl*_ being the coefficient. The multistate exchange–correlation matrix function *E*_xc_[*D*(**r**)] is defined by eqn (55), accounting for the remaining correlation energy not included in the multistate active space.

Notice that I have introduced a new class of density functional, the transition density functional (TDF) *E*^AB^_c_[*D*(**r**)] between states A and B.^458^ The physical interpretation of the TDF is the dynamic correlation contribution to the electronic coupling between two basis states.^457,458^ Although the functional form of the TDF is not known (similar to the exchange–correlation functional in Kohn–Sham DFT), in special cases such as spin-coupling interactions, it can be determined with the constraint of spin-multiplet degeneracy with the high-spin state determined separately using Kohn–Sham DFT.^457,458^ Obviously, as in Kohn–Sham DFT, *E*^AB^_c_[*D*(**r**)] also includes the corresponding residual kinetic energy not expressed in the orbital term.

One way to construct the multistate active space is to use constrained Kohn–Sham determinants for the states of interest. These non-Aufbau configurations can be optimized *via* a ΔSCF procedure,^461^ or by the targeted orbital optimization method.^462^ If we do not simultaneously optimize {*c*_*AI*_,*a*_*pA*_} in eqn (52), the orbitals in each determinant will be separately optimized, and they are generally nonorthogonal. Then, the procedure is equivalent to nonorthogonal state interaction (NOSI), a convenient approximation to the full MSSCF solution.^454,455^ In this case, one first carries out the optimization of each determinant configuration as a constrained Kohn–Sham DFT calculation.^455,463^ Then, this is followed by a single step of diagonalization of the Hamiltonian matrix functional (eqn (54)), with the approximations for the off-diagonal elements *H*_*AB*_ given in ref. 454, 455 and 457 to yield the energies of the adiabatic states. Consequently, all adiabatic states in the subspace, including the ground state, are treated on an equal footing in the course of minimizing the multistate energy functional.^458^

Since state interactions are explicitly included in the active space that defines the multistate energy, the effect of interference highlighted in contribution (3.7.1) is naturally included in MSDFT.

### 3.8 What has DFT told us about the real world?

#### 3.8.1 Jones

DFT would not be “the workhorse of quantum chemistry and materials science” if it had not provided much information about the real world. This is obvious in areas of particular interest to me, where its ability to make useful predictions of interesting physical properties broke new ground.

However, this is not the main lesson that DFT has taught *me* about the “real world”. I participated in (and survived) the struggle of a small number of scientists to convince the overwhelming majority of theoretical chemists that density-functional calculations could play an important role in chemistry. This struggle (from the mid-1970s to the breakthrough to general acceptance in the early 1990s) was against a conservative community that, with few exceptions, did not hesitate to “vilify” (Baerends, personal communication), ridicule, or ignore a development that everyone now knows was in its own interests. Such long-running rejection of unfamiliar ideas is certainly not unique in science, but I hope that it will not be repeated in the density-functional community.

#### 3.8.2 Salahub

Jones's answer about DFT being vilified in the early days reminds me of a tongue-in-cheek paper I wrote in 1999 as part of a *Theor. Chem. Acta* series reviewing contributions of DFT to end-of-the-century applications.^464^ Here is the beginning:

“1 am. A faint knock on the downstairs door. Or was it? Then the unmistakable thump of a heavy boot against the door and the crack of the door jamb as it shattered. Had his sordid past caught up with him? The interrogation would be swift and on the spot. Where did that wooden chair come from? And the bare light bulb slowly swaying above it? Whose face was that, almost invisible behind the glare? Inquisitor: Are you now or have you ever been a member of the Xalpha party? Mild-mannered respectable density-functional-theory practitioner (MMRDFTP): What? (Where had he heard that voice before?) Inquisitor: Are you now or have you ever been a member of the Xalpha party? MMRDFTP: I’m a Mild-mannered respectable density-functional-theory practitioner (MMRDFTP). What do you mean by breaking into my house in the middle of the night and hauling me out of bed like that? I was just in the middle of a great dream about an exchange–correlation functional that had the right asymptotic form and took care of dispersion seamlessly. Could have done excited states too, and eminently parallelizable. And now I’ve forgotten what it looked like”

Perhaps this has something to do with the “real world”.

#### 3.8.3 Schwerdtfeger

I remember the days when John Pople and Walter Kohn each argued their case of what the future will be, wave-function or density-functional based. It is clear that we can reach unprecedented accuracy in wave-function-based theory, testing even the standard model of physics. A nice example here is the accurate determination the fine structure constant from QED.^465^

And to make it clear: wave-function-based theory should be used wherever it can be used. But this is exactly the point. DFT is applied for large systems because of its low computational scaling law, *O*(*n*^3^), with the number of particles involved *n*, and where wave-function-based theory has (and in future will still have) real problems – for example, in describing electron correlation for strongly correlated and metallic systems. The electron-correlation problem for metallic systems in wave-function-based theory was already pointed out in early days by Fulde.^466^ Here, DFT gave us many useful results of the “real world” where *ab initio* theory is just not able (yet) to do the same job. It has become therefore an invaluable tool for materials science, solid-state physics, and the simulation of biomolecules.

What is perhaps a bit annoying (at least to me) are the “quick fixes” applied to DFT when one does not get reasonably accurate results – I just mention here the better description of electron pairing due to the on-site Coulomb repulsion by the use of the Hubbard term in DFT + *U*.^467,468^ On the other hand, many-body theory can be used successfully within a DFT formalism as the many applications in solid-state physics show – for example, by using *GW* and Bethe–Salpeter theory leading to quite accurate solid-state properties. So the two worlds come together somehow.

#### 3.8.4 Chermette

Young researchers may be reminded of the difficult youth of DFT in chemistry, even though interesting results were obtained^469,470^ and interpreted^471^ in the 1970s and 1980s. A similar situation occurred in other domains, such as the quasi-crystal discovery, not accepted by the crystallographers for a while.^472^

#### 3.8.5 Kronik

DFT interacts with the “real world” in three ways:

##### Confirmation

Sometimes experimental findings can be conflicting or controversial, owing to sample quality, complexity of measurement, difficulty of interpretation, or all of the above. “Reproducing” the experiment on the computer, using DFT, allows theorists to weigh in on such controversies.

##### Interpretation

Often the experimental result is beyond dispute, but it is poorly understood. With DFT, we can easily test for the effect of, for example, adding, moving, or removing an atom; we can examine the role of transition states and metastable states; we can assess what individual (Kohn–Sham) electron orbitals do and more. By doing so, we can explain experiment. The same tasks would range from the exceedingly difficult to the *a priori* impossible if attempted experimentally.

##### Prediction

Suggesting new mechanisms and properties before they have been examined experimentally, or indeed even suggesting new useful molecules or materials before they have been synthesized, once seemed like a distant “holy grail”. It is a testament to the quality of modern DFAs that such predictions are becoming increasingly successful.

#### 3.8.6 Gritsenko

DFT provides an astonishing example of how, arguably, the most exotic ultra-nonlocal feature of Kohn–Sham theory supplies the missing piece of information about one of the most important experimental characteristics of solid-state physics: the fundamental band gap *E*_g_. The feature in question is a finite upward jump *Δ*_xc_ of the Kohn–Sham exchange–correlation potential of a bulk crystal with a finite *E*_g_, when just a single electron is added to the conductance band. Addition to the too low Kohn–Sham band gap *E*^KS^_g_ of a simple estimate of *Δ*_xc_ extracted from the Gritsenko–van Leeuwen–van Lenthe–Baerends (GLLB)^321^ or Becke–Johnson (BJ)^473^ model exchange–correlation potentials produced surprisingly good-quality *E*_g_ for many extended systems.^474^

#### 3.8.7 Galli

DFT has told us about trends in properties and chemical bonding in numerous molecular and condensed phases, in spite of inherent inaccuracies of existing DFAs, and has shown predictive power and great usefulness in interpreting experiments. DFT has also been overused and those instances should of course be corrected. It should be emphasized that DFT is at the basis of all MBPT studies and even of quantum Monte Carlo calculations of solids, where the starting wave function is in most cases constructed from DFT orbitals.

#### 3.8.8 Neese

There is no doubt in my mind that computational chemistry would not be nearly as popular and important in chemistry as it is today without the huge success that DFT has enjoyed. While linear-scaling wave-function-based approaches have a come a very long way and can now be routinely used in most computational chemistry studies, I do not foresee that even the best linear-scaling approaches will make DFT obsolete in any shape or form. For example, it is difficult to see how correlated wave functions could compete with the speed and accuracy of DFT for geometries and harmonic frequencies. On the other hand, correlated wave functions are conceptually and numerically superior to DFT for a number of properties, for example magnetic properties. Personally, I hope for a fruitful interplay and co-existence, in which computational chemists make the best use of the available computational tools, no matter what theoretical framework they are based on.

#### 3.8.9 Barone

What is the meaning of the “real world”? All computations are performed on model systems, so that comparison with experiment requires the definition of both the mathematical (here the DFA) and the physical (the system investigated). Since DFT allows us to increase the dimensions of the physical model more than is possible with wave-function methods, we come closer to the “real system”. As a result, the discrepancies with experimental results are more probably related to deficiencies of the mathematical model. Of course, here multilayer (QM/QM′) models play a significant role, at least for nonperiodic systems.

#### 3.8.10 Helgaker

I wonder whether we as a community would have known less about the role of dispersion in chemistry and physics if DFT had not struggled to describe it. By being able to turn on and off dispersion as described for example by Grimme,^384^ we have observed the effects of dispersion in a very transparent manner – this was for me, at least, an eye-opener. In general, DFT forces us to discuss the real world in a different manner than wave-function theory.

### 3.9 What is the status of DFT-based tools for interpretation of chemical phenomena?

#### 3.9.1 Ayers, Chattaraj, Chermette, De Proft, Fuentealba, Geerlings, Liu, Vela, and Yang

The role of conceptual DFT in this endeavour has been very important: its past and recent accomplishments have been summarized in earlier reviews^188,189^ and a recent “status” paper^475^ where also its present status, prospects and issues are scrutinized. The present status can be best understood by considering the aim of conceptual DFT and the philosophy behind its realization.

The aim of conceptual DFT was clarified at a conference in Changsha City, China in 2018, attended by almost all of the most influential workers in the field, and formulated in the above mentioned status paper as “to develop a nonempirical, mathematically and physically sound, density-based, quantum-chemical theory for interpreting and predicting chemical phenomena, especially chemical reactions”.^475^ This aim should be realized with a philosophy based on three fundamental precepts: *observability* (our understanding of chemical observations should be based on quantum-mechanical observables – in particular, the energy, the density and their derivatives); *universality* (the results should not depend on the type of calculations) and *mathematical rigour* (aiming at a well-defined mathematical framework).

Based on these precepts, conceptual DFT has introduced a number of molecular reactivity descriptors, mostly response functions or descriptors derived from the *E*(*N*) curve, that – either alone or in combination with the electronegativity-equalization principle, the hard/soft-acid/base principle, the maximum-hardness principle, or the minimum-electrophilicity principle, for example – have served as valuable tools for the interpretation of experimental and theoretical (computational) data for a wide variety of reactions. Its scope comprises “generalized” acid–base, complexation and redox reactions and a multitude of “classical” organic reaction types including pericyclic reactions, with substrates varying from inorganic to organic and organometallic molecules, to polymers and the solid state. The success of conceptual DFT in pervading a broad range of chemical subdisciplines – from inorganic, organic, and organometallic chemistry to biochemistry and materials chemistry – can be described as getting “insight from numbers”, experimental or theoretical.

Pitfalls and shortcomings are still to be coped with, however, both on the more fundamental issues (*e.g.*, the nature of the *E*(*N*) function and the issue of differentiability, the convergence of the *E* = *E*[*N*, *v*] perturbation series in *N* and *v*) and on more applied aspects (*e.g.*, the delineation of the scope of the various principles) before the next step, from interpretation to prediction, can safely be taken; see Section 4.8.

#### 3.9.2 Baerends

On the topic of differentiability of *E*(*N*) hinted at in contribution (3.9.1), let us note that there truly is a fundamental problem.^476^ In the Euler–Lagrange equation for the optimization of the density,58
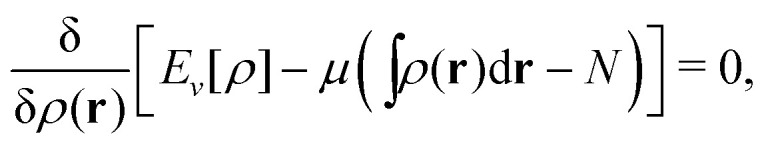
the total functional derivative of *E*_*v*_[*ρ*] has to be broken down into its partial derivatives59
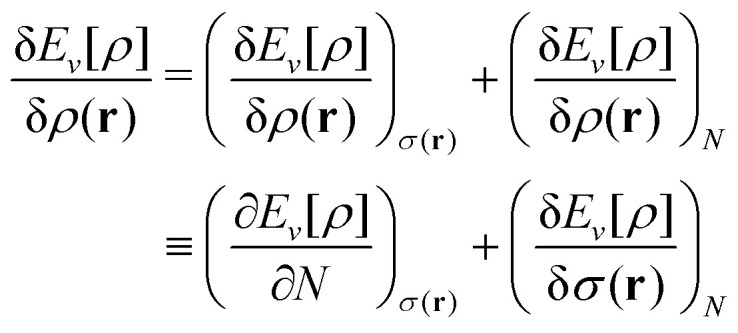
Here, the density is written as a product *ρ*(**r**) = *Nσ*(**r**) of the particle number *N* with a shape function *σ*(**r**) that integrates to 1.^477^ According to the theory of optimization under constraints, the Lagrange multiplier *μ* at the optimum density *ρ*^*N*^ is equal to the partial derivative with the shape function held constant, ∂*E*/∂*N* for short. But the Hohenberg–Kohn theorem does not define the energy for densities with a noninteger number of electrons. So the energy for a density with noninteger *N* in a neighbourhood of *ρ*^*N*^ is not defined and the derivative does not exist.

The typical solution in the theory of variations under constraints is to choose some extension of the functional into the domain where it is not defined. This extension is essentially arbitrary, the only requirement is that the extension obeys continuity properties so that the derivative exists. At this point, the Lagrange multiplier, which is the force of constraint keeping the density at integer *N*, is undetermined. It is determined by the chosen extension of *E*[*ρ*] into the noninteger domain. This is not a problem, it is directly related to the well-known gauge freedom of the Kohn–Sham potential, to which an arbitrary constant may be added.^476^ In a widely cited paper by Parr *et al.*^186^ (see also contribution (2.5.3)), the Lagrange multiplier *μ* has been described as “a characteristic constant for a system”, but without any proof or arguments. This contradicts the essential arbitrariness of the constant *μ*, and therefore of ∂*E*/∂*N*. Atoms and molecules have an ionization energy and an electron affinity – there is no additional physical quantity *μ* = ∂*E*/∂*N*.

The best known choice for extension of *E*_*v*_[*ρ*] into the noninteger domain is the one of ref. 18 – namely, forming an ensemble of the ground-state density matrix of the *N*-electron system with either the density matrix of the ground state of the (*N* + 1) system or the ground state of the (*N* − 1) system. This procedure leads to piecewise linear energy behaviour. This choice precludes application of the Euler–Lagrange variation method because the derivative ∂*E*/∂*N* does not exist (is discontinuous at the integer point). More extensive discussion of these matters is given in ref. 478.

#### 3.9.3 Liu

Two schemes for partitioning the total energy in DFT have been applied to understand different chemical processes and transformations.^193^ From these schemes, a unified view of molecular conformational stability has emerged, in which the electrostatic interaction plays the dominant role, while the contributions of steric repulsion and quantum effects are minor yet indispensable.^193^ This was also recently utilized to analyse the effects of cooperativity,^479^ frustration,^480^ and homochirality.^481^ Regioselectivity, nucleophilicity, and electrophilicity have also been quantified by information gain and Hirshfeld charge.^193^ Recent studies of density-based quantities for aromaticity and antiaromaticity yielded two opposite propensities, one for aromaticity and the other for antiaromaticity, depending on the number of π-electrons.^193^

#### 3.9.4 Chermette

As pointed out in contribution (3.9.3), for most molecular systems, the electrostatic interactions dominate over steric repulsion and other quantum effects. Accordingly, an analysis of the molecular perturbation introduced by a small charge (typically ±0.1*e*) leads to interesting insights in the understanding of reactivity.

The perturbed energy can be analysed as a contribution from excited configurations, whose importance may be estimated by their oscillator strengths. In most cases, it appears that only a limited number of excitations contribute significantly to the overall response to the perturbation, suggesting that chemical reactivity can be predicted by analysing the reshuffling of electron density upon excitation.^482^ The stabilization energy due to interaction between the polarization density and the electrostatic potential δ*v*(**r**) is given by60
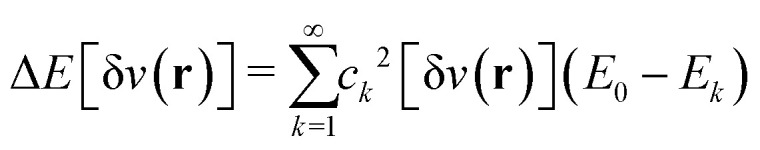
where *c*_*k*_^2^[δ*v*(**r**)] is the oscillator strength of the *k*th excited state and *E*_0_ − *E*_*k*_ is minus the *k*th excitation energy.^483^ Therefore, eqn (60) can be viewed as a minus the energy required to rearrange the electron configuration so that *c*_*k*_^2^ electrons are promoted from the ground state to the *k*th state. Following the same line of thought, the plot of *c*_*k*_^2^*versus* (*E*_0_ − *E*_*k*_) can be considered as a polarization spectrum. The polarization density can be computed as61
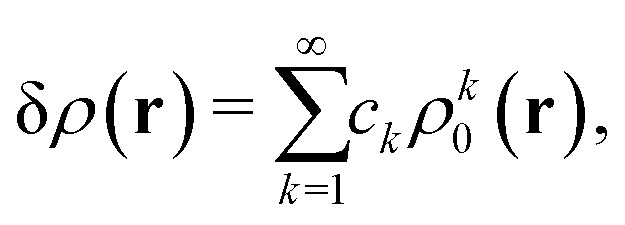
where *ρ*^*k*^_0_(**r**) is the transition density coupling the *k*th state to the ground state.^483^

From a link between conceptual DFT and statistical thermodynamics, it has been shown that the perturbation energy due to intermolecular electrostatic interactions can be understood in terms of effective work and heat exchange,^484^ the first-order correction 
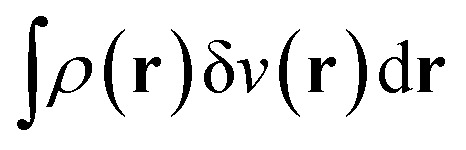
 being the effective work and the second-order correction in eqn (60) being the heat exchange. A polarization entropy and a polarization temperature can also been defined by this analogy. Therefore, using the external electrostatic potential as a probe and the polarization energy, entropy, and density as electronic responses, one can get qualitative and quantitative insight into the reactivity and the selectivity of molecular fragments.^483^

#### 3.9.5 Fuentealba

One should not forget that, along with density functionals, one has density functions as a special case. Ramon Carbó-Dorca Carre has studied the mathematical structure of such functions.^485^

#### 3.9.6 Ayers

One advantage of the popularity of DFT is that it gave publicity to methods based on the direct analysis of the electron density (and higher-order electron distribution functions), some of which developed concurrently with, or even predated, the emergence of modern DFT. Simply stated, DFT is a useful method not only for predicting reactivity as discussed in contribution (3.9.1) but also for describing and characterizing molecular electronic structure. Indeed, the framework of the quantum theory of atoms in molecules (QTAIM)^486^ and more generally quantum chemical topology (QCT)^487^ were largely developed alongside DFT, and use the same quantities (notably the density and its derivatives, various energy densities, and various strategies for characterizing, representing, and approximating the exchange–correlation hole) to obtain insight into molecular structure and chemical bonding.

## 4 The Future of DFT and DFAs

### 4.1 What are the important lines of development in DFT and for DFAs?

#### 4.1.1 Gritsenko and Pernal

Importantly, DFT can resolve a bottleneck problem of wave-function theory and DMFT regarding the reliable description of dynamical electron correlation. Indeed, nondynamical correlation can be efficiently accounted for with the small CAS CI and DMRG *ab initio* approaches or with DMFT functionals of the extended Löwdin–Shull (ELS) type,^64^ all in relatively small basis sets. It is the description of the residual dynamical correlation, which requires the inclusion of prohibitively many CI excitations in a sufficiently large basis, or many very weakly occupied natural orbitals, which is difficult to achieve with approximate DMFT functionals.

This bottleneck problem has been efficiently resolved in the CASΠDFT^488^ and ELS+ (the extension of the above mentioned ELS method)^64^ methods, which share the following master formula for the electronic energy:62
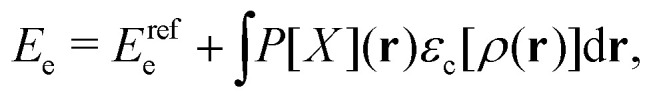
Here *E*^ref^_e_ is the CAS or ELS electronic energy, *ε*_c_ is a standard DFT correlation energy density functional, while *P*[*X*] is a scaling factor depending on the CAS or ELS on-top pair density *Π*(**r**, **r**) and the density *ρ*(**r**):63
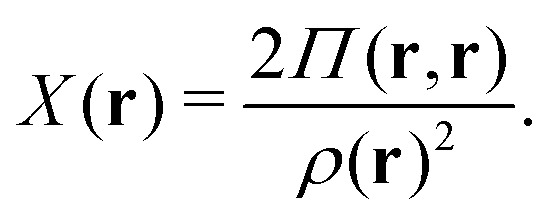
Effectively, the dynamical correlation energy is a functional of *Π*(**r**, **r**) and *ρ*(**r**) and the method works because *X*(**r**) locally probes the effect of nondynamical correlation exerted on the dynamical correlation. More precisely, the region where *X*(**r**)≤1 represents suppression of dynamical correlation by nondynamical correlation, while the region where *X*(**r**) > 1 indicates enhancement of dynamical correlation in excited states of ionic type.^488^ The CASΠDFT and ELS+ methods reproduce well, in a relatively small orbital basis, the accurate potential-energy curves in the complete basis-set limit; also, the CASΠDFT method yields good-quality vertical excitation energies for prototypical molecules.^489^ This development shows a promising new direction of combining *ab initio* methods with DFAs *via* the on-top pair density correlation functional.

#### 4.1.2 Gagliardi

The on-top pair density is the diagonal part of the two-body density matrix in the coordinate representation. It plays a very general role in wave-function theory and has also been used in many contexts in DFT and DMFT with both single- and multiconfigurational reference states. Many examples of multiconfigurational DFT have shown that use of the pair density gives superior results. One recent utilization of the pair density has occurred in multiconfigurational pair-density functional theory (MC-PDFT).^490,491^ In MC-PDFT, the energy is computed by combining wave-function theory for the classical components of the electronic energy (kinetic energy, electron–nuclear attraction, and classical electron–electron repulsion) with a functional for the nonclassical components of the energy (exchange and correlation). MC-PDFT is a special case of multiconfigurational nonclassical functional theory (MC-NCFT).^492^ The expression for the MC-NCFT energy is

64 *E*_MC-NCFT_[*ψ*^MC^] = *E*^MC^_class_ + *E*_nc_[*f*[*ψ*^MC^]]

where the classical energy *E*^MC^_class_ contains the nucleus–nucleus repulsion, nucleus–electron attraction, classical electron–electron repulsion, and electronic kinetic energy. The nonclassical functional *E*_nc_ depends on a featurization *f* of the reference wave function *ψ*^MC^, which may be the density, on-top density, and their gradients, or other attributes of the wave function – it can, for example, be the on-top functional *E*_ot_. MC-PDFT does not use wave-function theory for the internal correlation energy. The method has shown promising performances in several applications involving ground and excited states of multireference systems.^493,494^ Analytic gradients for the state-specific and state-average formulations are available.^495,496^

There are two main issues that should be addressed in the future, if MC-PDFT is to become a routine method for multireference systems:

1. How should one choose reference wave functions for these calculations and make them affordable for extended systems? Instead of using the CASSCF wave function, one can use RASSCF or generalized-active-space SCF (GASSCF) wave functions or DMRG wave functions.^497^ Related to this challenge is the task of automating active-space selections to make these methods more user friendly.

2. The second direction of development is towards the functional form. Currently, functionals are borrowed from the Kohn–Sham world, but specific functionals for this theory should eventually be developed. One possibility is to develop multiconfigurational density-driven functional methods that correct the classical or total energy of a multiconfigurational wave-function method through the use of a machine-learned functional.^492^

#### 4.1.3 Jensen

A promising alternative to variants of single-determinantal Kohn–Sham DFT, including hybrid and long-range corrected DFT, is to use range separation not only for the exchange energy but also for the correlation energy, as originally suggested by Savin^158^ and mentioned by him in contribution (2.2.24); see also contributions (2.5.6) and (2.5.7) from Pernal.

The separation of the electron–electron repulsion into a long-range (lr) part and a short-range (sr) part is usually achieved with the error function erf(−*μr*_12_) where *μ* is an interaction-strength parameter. By means of the AC, one obtains a continuous range of possible DFA models defined by different values of *μ* ≥ 0. In particular, one obtains Kohn–Sham DFT for *μ* = 0 and pure wave-function theory (WFT) for *μ* →∞; the AC from *μ* = 0 to *μ* = ∞ thus provides an alternative path to Kohn–Sham DFT.

The more interesting case is to use the AC from a partially interacting system at finite *μ* > 0 to *μ* = ∞. One then obtains a hybrid lrWFT–srDFT model, which, for a sufficiently large *μ*, can describe nondynamical long-range correlations adequately and also give correct spin symmetry of open-shell molecules^498^ by means of a multideterminant reference wave function. This approach is much simpler than attempting to describe them with a complicated Kohn–Sham DFA functional based on a single determinant. On the other hand, the dynamical short-range correlation effects can be described efficiently with a semilocal srDFT functional connecting to a Kohn–Sham DFT functional in the *μ* = 0 limit.

In the hypothetical case of short-range exact density functionals and long-range FCI wave functions for any *μ* value, the total energy would be the same for all *μ* values. The idea is to use the *μ* value for which the least computational work is needed to obtain good-quality energies and properties.

The computationally most efficient lrWFT–srDFT model will be for the smallest *μ* value for which long-range and spin correlations can be described to the desired accuracy, as this leads to the most compact lrWFT part with the smallest number of active orbitals. By comparison with accurate wave-function calculations, it has been found that a value around *μ* = 0.4 can be considered universally applicable for valence properties of singlet molecules.^499–501^ Preliminary investigations of transition-metal complexes indicate that a value around *μ* = 1.0 may be needed to describe their spin correlation sufficiently well.^502^

A particularly promising choice for the lrWFT part is to use the variational multiconfigurational self-consistent field description, leading to an lrMCSCF–srDFT (MC–srDFT) model. Because it is variational, the MC–srDFT model can also be used for molecular response properties, just like TDDFT in Kohn–Sham DFT – not only for excitation energies and transition moments, but in general for optical, electrical, and magnetic perturbations. For molecules with strong nondynamical correlation, the kinetic energy will be better described with the MC–srDFT model than with Kohn–Sham-DFT, thus a smaller kinetic-energy correction needs to be described by the correlation functional.

Note also that the MC–srDFT approach can be used not only for electronic ground states, but also in state-specific models for excited electronic states of any spin multiplicity and spatial symmetry.

#### 4.1.4 Loos

Recent developments by Giner, Toulouse, and coworkers on DFT-based basis-set corrections for wave-function theory (based on the range-separation of the electron interaction) are particularly promising with respect to removing, at a low computational cost, the basis-set incompleteness error in high-level calculations.^503^

#### 4.1.5 Kronik

The concept of “optimal tuning” has proven to be highly useful for extracting accurate one- and two-electron excitation energies from (relatively) simple DFAs.^91^ In DFT research, we typically seek an increasingly general DFA, which can come as close as possible to the ideal of a universal functional. But this comes at an increasingly large computational cost. Optimal tuning deviates from this paradigm. It seeks to retain a reasonably simple, low-cost general functional form, in which one or two parameters remain undetermined. The remaining freedom affords enhanced accuracy, with the parameter(s) determined nonempirically, but in a system-specific way, by demanding that a physical constraint be obeyed.

The most successful practical incarnation of this idea has been based on another highly successful idea – namely, that of range-separated hybrid functionals.^106,504^ These functionals can exhibit an asymptotically correct long-range (free^91^ or screened^505^) Coulomb potential, while retaining a useful balance between exchange and correlation in the short range. The range-separation parameter is then tuned, per system, by enforcing the ionization-potential theorem^18^ (or variants thereof). The approach has been successful in overcoming issues considered very challenging for DFAs, notably the infamous band-gap problem^506,507^ and the prediction of charge-transfer excitation energies,^508,509^ through systematic elimination of derivative discontinuity errors. Importantly, this approach restores the physical picture of single- and two-quasi-particle excitation thresholds, by reliably predicting them from the HOMO–LUMO eigenvalue difference of a DFT calculation and the lowest eigenvalue of a TDDFT calculation, respectively, using the same exchange–correlation functional.^91^ Extensions of these ideas can be expected to continue to play a role in DFT applications to spectroscopy.

#### 4.1.6 Johnson

Development of a DFA that can eliminate delocalization error is needed. The delocalization error, also known as the many-electron SIE, refers to the tendency of many DFAs to overstabilize systems with highly delocalized electrons or fractional charges on separated moieties.^17,510,511^ This error affects charge-transfer complexes, extended hydrogen-bonding networks, halogen bonds, organic salt crystals, systems with extended π-conjugation, and transition states of many radical reactions, to list a few examples. It is also responsible for the notorious band-gap problem.^21,67,512^

While many approaches to reducing delocalization error have been proposed, none is a panacea. Typically, one can reduce delocalization error through a (frequently range-dependent) mixing of local, DFA exchange and nonlocal, Hartree–Fock exchange. However, the optimal mixing is known to be highly system and size dependent.^513,514^ Development of a practical and universally applicable DFA with minimal delocalization error remains an outstanding challenge and would represent a significant advance.

#### 4.1.7 Gould

It is likely that any advance on the delocalization-error front would also help to resolve some of the issues with strong correlation, given how closely linked the two problems are. DFAs from Gori-Giorgi and Vuckovic that are based on the SCE limit (see contribution (2.4.5)) offer some innovative ways of thinking about both problems.

#### 4.1.8 Görling

A promising line of work in DFT is the development of correlation functionals based on the ACFD theorem.^100,101^ Such correlation functionals are used in conjunction with an exact treatment of all other parts of the total energy, obtained by simply evaluating the expression of the Hartree–Fock total energy with Kohn–Sham orbitals. The simplest of these correlation functionals is based on the RPA.^102–104^ Already the RPA yields competitive reaction and transition-state energies but can in addition treat noncovalent interactions.

There are various ways to go beyond the RPA. Highly promising are σ-functionals,^515,516^ which are technically closely related to the RPA but formally rooted in many-body perturbation theory along the AC. Methods using σ-functionals are distinctively more accurate than RPA-based or conventional functionals – they reach chemical accuracy for main-group chemistry and treat noncovalent interactions accurately.

While some methods based on the ACFD theorem are computationally expensive, this is not at all true for functionals within the RPA or for σ-functionals. These are typically evaluated in a post-SCF way, ideally using orbitals and eigenvalues from hybrid DFT methods. In this case, the post-SCF calculation of the total energy using the RPA or the σ-functional requires less computational time than the preceding hybrid calculation and thus can be easily carried out routinely.

There is much room for further developing correlation functionals based on the ACFD theorem and these functionals open up a new area for DFT – the field of highly accurate electronic-structure calculations, so far dominated by wave-function methods like the hierarchy of coupled-cluster methods.

#### 4.1.9 Xu

Despite being highly successful for main-group chemistry,^273,336,517^ the PT2-based double-hybrid approximations inherit the intrinsic deficiency of the PT2 correlation model for nondynamical correlation, which hinders their application to some of the challenging problems of DFT, such as stretched H_2_ and other molecules (see contribution (3.4.1)) without symmetry breaking (see 3.6) and transition-metal complexes (see contributions (3.2.2) and (4.1.11)).

A simple replacement of standard PT2 by more sophisticated correlation models from wave-function theory does not seem to lead to a notable improvement in accuracy despite the higher cost.^518^ Some recent efforts to develop efficient models that go beyond PT2 for double-hybrid approximations have led to some encouraging schemes for further progress.^519–522^

#### 4.1.10 Kaupp and Arbuznikov

One generalization of the concept of hybrid functionals that tries to account for local differences in the relative importance of exchange and correlation as well as for the differing spatial demands of different property operators is to use position-dependent Hartree–Fock exchange admixture in local hybrid functionals (LHs).^318,319,523^ While this introduces the ambiguities of locally mixing exchange-energy densities (the “gauge-problem” of LHs^524–527^) and some (manageable) additional requirements regarding two-electron integrals compared with standard (“global”) hybrids, the advantages of position-dependent exchange admixtures for various properties depending on different regions of space have been demonstrated.^312,523,528,529^

These advantages extend also to TDDFT computations of various types of excitations, including core, valence, and Rydberg excitations, with particularly good performances for triplet excitations.^530,531^ In general, LHs give extra flexibility to balance minimal self-interaction or delocalization errors in some regions of space with the simulation of left–right correlation in bonds. LHs can be further extended in various directions by combining with range separation (*e.g.*, local range separation^532–534^ and range-separated LHs^535,536^), by adding dispersion either *via* correction terms or *via* nonlocal van der Waals functionals, potentially by adding nonlocal rung-5 correlation contributions, or by adding corrections for strong correlation.

#### 4.1.11 Xu

There is an ongoing effort devoted to the development of reference data sets that are sufficiently accurate for benchmarking functional performance.^537,538^ A recent progress in the community is the emergence of large data sets for the main-group chemistry – for example, the MGCDB84 set with about 5000 data points maintained by Head-Gordon's group^273^ and the GMTKN55 set of Grimme's group with about 1500 data points.^336^ Comprehensive benchmarking of existing DFAs for main-group chemistry has become a reality, numerically validating the concept of Jacob's ladder of Kohn–Sham DFT by demonstrating that a higher-rung DFA is, in general, more accurate than a lower-rung DFA.^336^

For transition-metal systems, the situation is more complex and less developed than for main-group systems.^539–541^ Large transition-metal test sets with accurate reference data are urgently needed. It is not merely important for benchmarking Kohn–Sham DFAs, but also fundamentally important for understanding the limitations of current Kohn–Sham DFAs for strongly-correlated systems as mentioned in contribution (3.4.1), since transition-metal systems often have a strong multireference character.

#### 4.1.12 Ruzsinszky

Density functionals at the meta-GGA level harbour a great potential that has not been fully exploited. The excellent performance of the SCAN and r^2^SCAN functionals and the deorbitalized meta-GGA versions^542,543^ work very well for structural and energetic properties. Less is known in practice about the capability of some meta-GGAs for fundamental band gaps^512^ and excited states. While the TB-mBJ potential^544^ delivers accurate band gaps, its accuracy originates from fitting.

The recent TASK^545^ and modified (mTASK)^546^ meta-GGA DFAs are energy functionals developed explicitly for band gaps. Both DFAs excelled in accuracy for band gaps of some important but limited test sets of bulk solids and two-dimensional materials. However, more tests should be done in order to reveal the strengths (and limitations as well) and applicability of these meta-GGAs. With more information, the TASK and mTASK approximations can compete with available hybrid functionals such as the HSE06 functional in accuracy, at a more favourable computational cost. Such applications for the fundamental band gap could initiate the development of exchange–correlation kernels for optical response properties constructed from these meta-GGA functionals. This latter possibility is an obvious advantage of DFAs that possess functional derivatives. The static exchange–correlation kernel from a DFA is its second functional derivative with respect to the density, which needs to exist for this approximation.

#### 4.1.13 Chattaraj

Orbital-free DFT with approximate interacting kinetic-energy functionals should be explored further.^547,548^

#### 4.1.14 Fuentealba

The question is: Is there any hope to get a relatively accurate kinetic-energy functional? It must be highly nonlocal. Machine learning (ML) may help.

#### 4.1.15 Trickey

Apropos ML and orbital-free DFT, a warning is in order about good answers for bad reasons. There have been several instances of machine-learning manuscripts that purported to provide a kinetic-energy density functional (KEDF) but the functional dependence on the density *ρ* was such that ordinary uniform scaling of *T*_s_[*ρ*] was violated.

#### 4.1.16 Carter

Orbital-free DFT simulations that utilize existing nonlocal KEDFs are already quite accurate for a number of properties of solid and liquid main-group metals^549^ – see, for example, an orbital-free DFT MD study of the dynamics of liquid tin in ref. 550 using our open-source orbital-free DFT code PROFESS 3.0.^551^ These nonlocal KEDFs – see ref. 552 for a software library – are successful for such nearly-free electron-like systems because they are directly derived from the physics of the perturbed free-electron gas (the Lindhard function); for recent analysis, see ref. 553 and 554.

The real challenge is to develop KEDFs that can describe molecules and transition metals (similar problems will exist for f-block elements). The inherent angular momentum dependence of the electron distributions – captured by orbitals but not by densities – makes KEDF development for far-from-uniform densities truly a grand challenge. Self-consistent all-electron calculations, without pseudopotentials/effective core potentials, are also a huge challenge due to the same issues. We have shown how difficult this is to accomplish in several papers, where we can achieve small wins but easily break our models as well; see, for example, ref. 555–559, as I believe strongly – as discussed later in contribution (4.4.4) – in the value of publishing failures to gain insight into how to advance the field.

#### 4.1.17 Trickey

We too have been working on orbital-free DFT with emphasis on one-point KEDFs designed to satisfy constraints. One can get surprisingly good forces from those, but at the cost (so far) of inaccurate (too high) energies.^560,561^ An oft-ignored requirement on KEDFs is their *N*-representability; see Ayers and Liu.^562^ See the preceding remarks by Carter about nonlocal functionals. Also note her remark about local pseudopotentials. It may be that orbital-free DFT is forced into all-electron calculations of a modified projected-augmented wave (PAW) type.

A crucial point for the orbital-free DFT agenda that often goes undiscussed is to get rid of the orbital dependence in DFAs. This is the antithesis of much of the activity in the quantum chemistry community, as much of the discussion in this roundtable confirms. We have made considerable progress on de-orbitalization of meta-GGA DFAs^542,543,563^ by inclusion of a dimensionless ∇^2^*ρ* dependence, though the success of that approach is quite dependent upon the numerical stability of the parent meta-GGA DFA.

#### 4.1.18 Carter

We also did some work on single-point KEDFs, based on pointwise kinetic energy density and ELF analyses, emphasizing approaches that enable self-consistent calculations.^564,565^ Most single-point GGA KEDFs are unable to converge densities self-consistently, with the VT84F KEDF of Trickey *et al.* being a notable exception,^560,561,564^ rendering most of them impractical for most applications.

Our single-point KEDFs have no problems converging and yield properties in good agreement with Kohn–Sham DFT for the usual materials – simple metals – but again, we can easily break them. Just study a vacancy or an alloy, both of which by contrast are handled well by our 1999 nonlocal WGC KEDF.^566,567^ Our 2015 pointwise analysis of our single-point KEDFs compared to the WGC KEDF indicates at least some of what is needed to help improve such single-point KEDFs^565^ – namely, reproducing of the inherent multivaluedness of the non-von-Weizsäcker component of the GGA enhancement factor when plotted against the reduced density gradient. The WGC KEDF, remarkably, does so, indicating yet another fundamental reason it is so accurate for simple metals.

#### 4.1.19 Carter

Regarding *N*-representability in contribution (4.1.17): this is more difficult than it seems – we worked on this for quite some time, without much success; see Chapter 7 of ref. 568.

#### 4.1.20 Liu

Relevant to KEDFs in orbital-free DFT is the Pauli energy, which has recently been employed to identify strong covalent interactions.^197^ If approximate KEDFs are utilized for the same purpose, miserable results are obtained.^569^ This quality appraisal test for approximate KEDFs shows that they are unable to accurately account for the kinetic energy distribution in the medium range away from nuclei, where chemical bonding takes place and the Pauli energy plays a crucial role.

#### 4.1.21 Teale and Helgaker

Recently, we addressed the issue of attempting to solve the Euler–Lagrange equation of orbital-free DFT in the all-electron context.^570^ Using a second-order optimization method based on the trust-region image method (TRIM),^386^ we could robustly solve the equation for many systems by simultaneously optimizing the density and the chemical potential in the saddle function 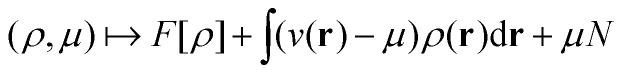. An interesting finding is that more complicated GGA-type functionals often show an erroneous nonconvex behaviour for the model *T̃*_s_[*ρ*] (where tilde indicates an approximate quantity). As a result, many solutions (rather than one solution) to the Euler–Lagrange problem with a given particle number *N* are found. Since *T*_s_[*ρ*] is the noninteracting limit of *F*[*ρ*] and since both functionals are convex with respect to the density variations, it would be interesting to explore techniques to impose convexity on approximate *T̃*_s_[*ρ*] and *F̃*[*ρ*] = *T̃*_s_[*ρ*] + *E*_H_[*ρ*] + *Ẽ*_xc_[*ρ*].

#### 4.1.22 Trickey

This remark from Teale and Helgaker is interesting because it suggests a different kind of constraint to impose in the construction of better KEDFs. It also will be important to see what the TRIM method does on a modern, constraint-based (all-electron) generalized gradient approximation such as our VT84F.^561^ By the way, since GGA KEDFs are inherently singular, a direction of interest to us is nonsingular combinations of reduced density derivatives.

#### 4.1.23 Vignale

Orbital-free approaches can be valuable not only in static DFT but also in time-dependent DFT. I would like to point out the existence of an orbital-free quantum-continuum-mechanics (QCM) approach,^571–573^ which offers an alternative to the time-dependent Kohn–Sham approach in calculating the dynamics of interacting electronic systems. This approach is based on the observation that the density *ρ*(**r**, *t*) and current *j*(**r**, *t*) of the many-body system obey the exact equations of motion65
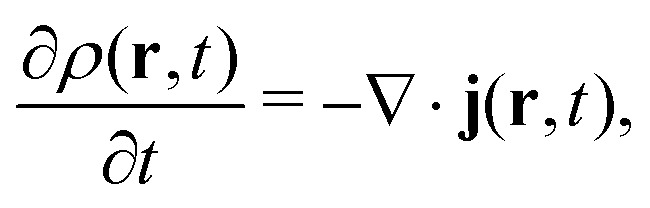
and66
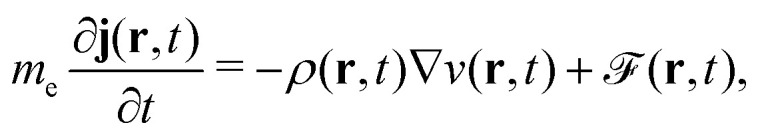
where *m*_e_ is the electron mass, *v*(**r**, *t*) is the potential and ℱ(**r**, *t*) is the force density arising from interactions between the particles. The calculation of the force density from the expectation value of the corresponding operator is a prohibitively difficult task: however, to the extent that we trust the basic tenets of time-dependent density and current DFT, we can assume that ℱ is a functional of the basic variables *ρ* and **j**. If an approximate form of this functional is adopted, then eqn (65) and (66) become a closed set of partial differential equations, akin to the equations of fluid mechanics, which can yield a huge amount of information about the evolution of the system without invoking the exact wave function. A particularly simple and appealing approximation to the force functional was proposed in ref. 571 and 572 for the linear response regime – that is, when the system is assumed to remain close to the ground state. The approximate force is then given by67
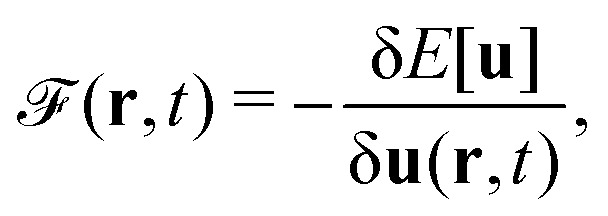
where *E*[**u**] is the energy (kinetic plus potential) of the state obtained from the ground-state wave function by applying a position- and time-dependent translation operator with displacement vector **u**(**r**, *t*). The displacement field is related to the current and the density by the relation68
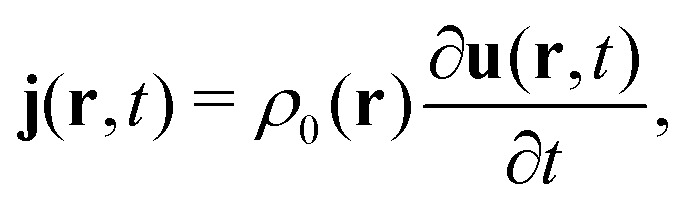
where *ρ*_0_(**r**) is the ground-state density and the functional derivative is evaluated to first order in **u**. This approximation was dubbed the “elastic approximation” in ref. 571 and 572 and reduces the problem of finding excitation energies to a standard eigenvalue problem with a dynamical matrix that is constructed from ground-state properties such as the pair correlation function and the one-particle density matrix. Only a few applications of this theory have been reported to date – see ref. 574 for a very recent one.

The elastic approximation is expected to work well for systems whose dynamics is dominated by collective modes, but not so well for independent-particle dynamics. There is much room for improvement, which makes this an exciting direction of research. Just to mention one possibility, the elastic approximation assumes that the force is instantaneously determined by the current: this leads to infinitely sharp excitation energies. We could go beyond the elastic approximation by introducing a physically motivated form of time retardation, which would immediately lead to more realistic spectra in extended systems.

### 4.2 What role will DFT play in multiscale and embedding methods?

#### 4.2.1 Salahub

I think Perdew's comment in contribution (2.2.1) situates DFT appropriately for multiscale modelling applications as providing “almost the right answer for almost the right reason at almost the right price”. QM/MM or embedding models have a “high-accuracy” method embedded in a “low-accuracy” method. In situations where DFT accuracy is good enough and if the speed is adequate, then DFT can be the high-accuracy method, usually coupled with an MM force field or various solvation models for the low-accuracy method. If DFT speed is an issue, then DFT can be used to calibrate faster semiempirical methods, like DFTB, again combined with an MM force field, for example. DFT can also be the embedding method as with frozen-density embedding theory, requiring kinetic-energy functionals.

#### 4.2.2 Carter

Beyond chemical applications, I want to remind readers of early work done to develop multiscale methods coupling quantum mechanics to higher-length-scale methods for studying materials properties, in order to simulate phenomena that cannot be handled properly by one scale alone – see, for example, this brief review from 15 years ago.^575^ While the coupled quantum–atomistic methods will be familiar to this readership (very much in the spirit of QM/MM and/or *ab initio* molecular dynamics/Monte Carlo), there are examples of coupling quantum mechanics (typically DFT) to continuum solid-mechanics methods, with feedback between scales that could offer ideas to build upon in the chemistry/physics realm going forward.

#### 4.2.3 Galli

Embedding techniques based on DFT are having an increasingly high impact in the study of highly correlated materials. There are many interesting problems that naturally lend themselves to a quantum-embedding description – for example, spin defects in solids or more generally point defects in materials, active site of catalysts, molecular adsorbates on surfaces, and nanostructures embedded in condensed systems, including solvents, to name a few. The great majority of embedding theories used in the literature today have some DFT component (*e.g.*, wave-function-method embedding in DFT, DMFT, and Green's-function-based embedding). In addition, using embedding theories, one may define second-quantized Hamiltonians and devise frameworks to carry out quantum-mechanical calculations for solids on near-term quantum computers – see, for example, ref. 576.

#### 4.2.4 Wesolowski

The Hohenberg–Kohn theorems and Kohn–Sham formulation of DFT are crucial for multiscale and/or embedding methods that apply multiplicative embedding operators (embedding potentials). The formal framework of frozen-density embedding theory (FDET) establishes the exact relations between the optimal embedded wave function, the embedding potential, and the Hohenberg–Kohn energy functional, for any nonnegative real function *ρ*_B_(**r**) used as the only quantum descriptor for the environment of an embedded system. For embedded wave functions obtained variationally, the FDET energy functional 
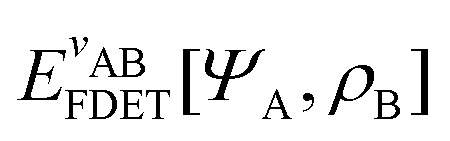
 satisfies the following equality by construction:^577^69
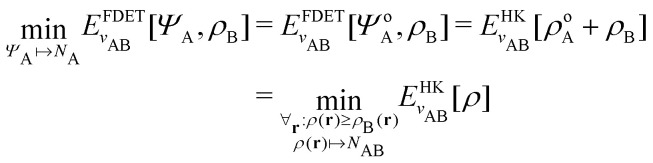
where 
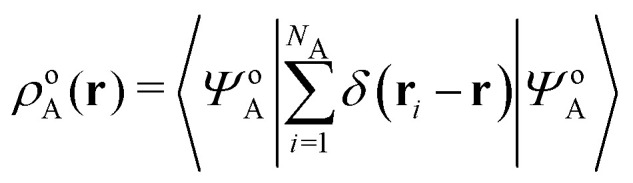
. Recently, an expression for the total energy that (similarly to eqn (69)) is consistent with the Hohenberg–Kohn energy functional, was derived also for methods in which the correlation energy is obtained as a nonvariational correction to variationally obtained wave functions.^578^ For whatever form of the embedded wave function, including the one introduced by Wesolowski and Warshel,^579^ FDET represents a bottom-up approach to deal with the quantum embedding problem in multilevel/multiscale simulations.

The optimal total density is the sum of individual components *ρ*_B_(**r**) and *ρ*^o^_A_(**r**) considered as independent variables in the total energy expression. Such a choice of independent variables makes it possible to use the information about the environment of the embedded species obtained from any physical model capable of delivering *ρ*_B_(**r**). Generating *ρ*_B_(**r**) using a wave-function description of the environment is one of several possible choices. Other choices include *ρ*_B_(**r**) obtained from continuum models of the environment,^580^ from X-ray diffraction data^581^ generated using a library of molecular electron densities,^582^ or from a simplified treatment of the electronic polarization.^583^ A particular version of FDET, where a noninteracting reference system is used for both *ρ*_A_(**r**) and *ρ*_B_(**r**) and where both densities are subject to optimization, is equivalent to Cortona's formulation of DFT.^584^

#### 4.2.5 Carter

The earliest FDET actually precedes Wesolowski and Warshel's 1993 paper; that credit should go to Warshel's, who proposed the first DFT-in-DFT embedding using KEDFs.^584^ As far as I am aware, the first WFT-in-DFT embedding utilizing this idea of an embedding potential containing a KEDF potential, as in FDET, was introduced by my group in 1998 in ref. 585, where we carried out, for example, MP4-in-DFT calculations for CO adsorbed on Cu(111).^585^ A follow-up, more detailed paper discussing our KEDF-based embedded correlated wave-function (ECW) theory also provides in the introduction important context of earlier embedded wave-function theories, for those interested in the history of the field.^586^ A 2008 review article summarizes the state of embedding (and other) electronic-structure methods for solids at that time.^587^ As Wesolowski points out in contribution (4.2.4), the FDET formalism subsequently was generalized by Wesolowski and coworkers to encompass methods beyond DFT, including a correlated wave-function treatment of subsystems.

However, for all the reasons summarized above, when discussing orbital-free DFT, the KEDF potentials introduce errors one would like to avoid, since we do not yet have KEDFs that reliably work across the periodic table. Because of this, more than 10 years ago we proposed density-functional embedding theory (DFET),^588^ in which one eschews use of KEDF potentials and instead uses OEP theory to solve for an exact (within a given DFA) embedding potential that describes the interaction between the embedded region and its environment. (Note that DFET is fully generalizable to more than two subsystems but, since we are mostly interested in fairly localized phenomena, typically two subsystems of a cluster of atoms embedded in a periodic slab background is sufficient.) This embedding potential thus solved for is then “frozen”, with no approximation other than the choice of exchange–correlation functional used to perform the OEP calculation. Since exchange–correlation functionals in use today are much more accurate and transferable across the periodic table than KEDFs, the embedding potential thus derived is much more accurate as well. This frozen embedding potential then is added as a one-electron operator to the cluster Hamiltonian. Thereafter, one can exploit readily any quantum-chemistry method for conducting the ECW calculations.

See ref. 589 for a brief review and ref. 590 for a more in-depth review of DFET^591^ and its cousin, potential-functional embedding theory (PFET).^592^ The latter can deliver self-consistent embedding potentials for hybrid ECW/DFT systems,^593–595^ albeit at considerable cost. Frankly, we have yet to find cases in which such self-consistency was terribly important, although I imagine such cases will emerge. If one is careful to include sufficient numbers of atoms in the embedded region such that the embedding potential does not overlap the phenomenon of interest but instead is essentially a physical boundary condition, then the frozen exact embedding potential we derive from DFET works very well. Thus we have continued to use DFET/ECW theory rather than PFET – with considerable success – to study problems where conventional DFAs fail, such as for phenomena involving electron transfer^596^ and excited states involved in electrochemistry and photochemistry on metals; for a recent review on the latter, see ref. 597. You are welcome to utilize our codes that compute embedding potentials and the AO-integrals in a variety of formats (see ref. 598) to try these calculations for yourself. There you can also find codes for a generalization of DFET to nonlocal embedding potentials that can also describe covalently bonded systems, in what we refer to as density-matrix-functional embedding theory (DMFET), where the same idea of using OEP is applied to density matrices rather than densities.^599,600^

Recent benchmarks that we have conducted comparing electrochemical carbon dioxide reduction modelled by a conventional DFA *versus* DFET/ECW theory reveals critical insights for modellers: for qualitative conclusions regarding reactions that do not involve electron transfer, the DFA is acceptable.^601^ By contrast, for any step involving electron transfer, specifically proton-coupled electron transfer (which we find to be the most favourable pathway), the DFA fails on multiple fronts (specifically it yields results inconsistent with experiments) due to too facile electron transfer (as expected from self-interaction error and the lack of a derivative discontinuity) whereas DFET/ECW predictions agree with experiments and produces qualitatively different products than the DFA^602^ – a cautionary tale for DFT modellers of electrochemistry.

Finally, we recently extended DFET/ECW theory to ionic/covalent materials; nearly all our previous work was done on metals. Before solving for the embedding potential with OEP theory, we cap the dangling bonds that were created at fragment edges by initial covalent bond cleavage, while partitioning atoms into subsystems. The capping eliminates potential spin-polarization artefacts that unpaired electrons at fragment edges would produce. The DFET theory is modified to account for the density of the capping atoms while solving for the embedding potential.^603^ An interesting sustainable energy application using this new theory examined metal-to-ligand charge-transfer states in a Ru-bpy dye attached to a titania cluster, as a model for such excitations in a dye-sensitized solar cell. The ECW calculations were conducted at the embedded CASPT2 level, predicting both lifetimes of singlet excited states and the positioning of triplet excited states, in order to consider the competition between fluorescent and phosphorescent decay.^604^

#### 4.2.6 Wesolowski

Owing to the consistency with the Hohenberg–Kohn energy functional, the formal framework of FDET provides a convenient tool for identifying the approximations/assumptions in any method that uses a multiplicative embedding operator. Multiplicative embedding operators are used commonly in QM/MM approaches, where they represent the classical electrostatic interactions, but also in various quantum embedding methods including those reviewed in contribution (4.2.5). A direct comparison of the expressions for the energy and the embedded potential of these methods with their FDET counterparts for each method discussed in contribution (4.2.5) is straightforward. For most of the methods, the approximations are easy to identify.

Concerning the potential-functional embedding theory (PDFT), FDET expressions for the total energy and the embedding potential admit also the embedded wave function and the environment density obtained from PDFT. In such a case, the total energies and embedding potentials of PFET and FDET are expected to be the same, in the absence of additional assumptions and approximations. In their exact form, both approaches target the same solutions. The identity of the corresponding quantities, one given as an explicit functional of the environment density (FDET) and one not (self-consistent PFET), might lead to a better understanding of the relevant density functionals.

Concerning Cortona's formulation of DFT applied originally to atoms in solids, it is worthwhile to recall an intriguing observation regarding LDA made when it was used for intermolecular complexes in our exploratory works.^605–607^ For such complexes, LDA is known to be inadequate for approximating the exchange–correlation energy. However, when applied simultaneously for both the exchange–correlation and non-additive kinetic-energy functionals, LDA yields surprisingly good interaction energies. The reasons for this apparent compensation of errors remains an open question.

#### 4.2.7 Piecuch

I have no doubt that DFT will continue to play a major role in the development of multiscale and embedding methods. I have been impressed by the ability of the FDET approach of Wesołowski and Warshel^579^ to compete with the considerably more expensive high-level equation-of-motion coupled-cluster calculations with singles, doubles, and noniterative completely renormalized triples (δ-CR-EOMCC(2,3)) in accurately reproducing the experimentally observed shifts of excitation energies due to hydrogen bonding.^608^

While this question may not belong to this section, and it may very well be that it is even ill-defined, our results obtained in ref. 608, especially the failure of the supermolecular TDDFT approach to produce accurate results for the same spectral shifts, made me wonder if practical implementations of TDDFT, which invoke a variety of approximations, satisfy the property of size intensivity of excitation energies (satisfied by properly developed methods based on equation-of-motion coupled-cluster theory). One might say that FDET and other embedding techniques are size intensive by design, which is yet another argument in their favour in applications involving excitation spectra in condensed phases.

We recently used the *ab initio* embedding scheme called the effective-fragment-potential approach^609^ combined with the aforementioned δ-CR-EOMCC(2,3) calculations, properly calibrated DFT and TDDFT methods to optimize geometries, and the DFT continuum solvation model based on the solute-electron-density approach^610^ to accurately model photochemistry of the strongest known super photobase abbreviated as FR0-SB in various alcohol solutions.^611^ This would not be possible without mixing DFT and TDDFT with embedding and *ab initio* approaches.

#### 4.2.8 Fromager

An alternative approach to quantum embedding initiated with the seminal work of Knizia and Chan^612^ on density-matrix embedding theory (DMET) has been intensively developed in the last few years in both condensed-matter physics^613–615^ and quantum chemistry.^616,617^ At first sight, DMET looks more like a wave-function-based method that has nothing to do with DFT. Nevertheless, connections can be made when the convergence criteria involve diagonal elements of the (one-electron reduced) density matrix only.^615,618,619^ In this context, the localized “impurity” orbital occupations 
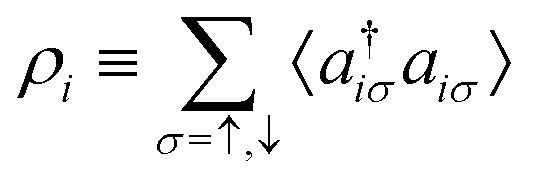
 play the role of the density and the correlation embedding potential,^616^ which is used in the full-size system, is a collection of one-electron (“on-site” in lattice models^615^) energies *v*_*i*_. The latter are adjusted so that the corresponding embedded impurity orbitals have the same occupation as in the full-size system. Referring to a noninteracting Kohn–Sham (full-size) system in this context is appealing because it is a way to “exactify” the embedding procedure.

In regular DMET, the bath orbitals (which exchange electrons with the impurity orbitals) are constructed from the Schmidt decomposition of the approximate mean-field wave function (which is computed for the full system). The resulting reduced-in-size “impurity + bath” cluster is then treated in wave-function theory as a closed system.

Sekaran *et al.* have recently shown that this procedure is equivalent to a (much simpler) density matrix-functional Householder transformation when the density matrix is idempotent.^615^ They have also shown that, when the transformation is applied to a correlated density matrix, the cluster becomes an open subsystem. Therefore, if the full system is described at the noninteracting Kohn–Sham level (which is still exact density-wise), then the usual separation of the cluster from its environment is perfectly justified. The correlation potential then learns from the cluster (in which interactions are reintroduced, after applying the Householder transformation) through the density constraint.

Thus, we obtain a new type of density-functional approximation (with an implicit dependence on the density) where we can afford an accurate description of strong electron correlation. Obviously, in general, the cluster's environment (which is usually neglected) contributes to the total correlation energy. A formally exact density-functional embedding theory would in principle be obtained by deriving, in this context, a multireference version of Görling–Levy perturbation theory.^133,449,450,615^

#### 4.2.9 Grimme

While Kohn–Sham-DFT calculations with accurate DFAs in combination with good one-particle basis sets are feasible for molecules with a few hundreds of atoms, they are still computationally too demanding for many purposes – for example in large scale screening applications, for the combinatorial problem of conformational sampling of flexible molecules, or the computation of vibrational Gibbs free energies of large systems. While the initial steps in typical multilevel approaches can be conducted routinely at a semiempirical or force-field level,^281^ at some point in the applied filtering procedures, higher accuracy is required – in particular, for relative (chemical) energies.

This motivated the development of composite Kohn–Sham-DFT methods – for example, from the 3c-family B97-3c or r^2^SCAN-3c^620,621^ at the (meta)GGA level. The sought-after compromise between computational effort and accuracy is achieved here by applying tailored, medium-sized atom-centred AO basis sets on top of standard or slightly modified DFAs and adding appropriate atom pairwise potentials to account for dispersion and basis-set incompleteness. The recently proposed r^2^SCAN-3c method outperforms some hybrid-DFT/QZ approaches for reaction and conformational energies as well as for noncovalent interactions at a speed-up of two to three orders of magnitude.^621^

#### 4.2.10 Köster

For QM/MM MD applications, Kohn–Sham DFT with density fitting or auxiliary DFT (ADFT) are very promising QM methods.^622^ In combination with DFT-optimized basis sets and automatically generated auxiliary functions, ADFT Born–Oppenheimer MD simulations on the nanosecond timescale are possible. These calculations permit the simulation of finite-system melting, to determine the corresponding melting temperatures and latent heats.^623^ The extension of these calculations to QM/MM models will allow the simulation of finite-system phase transitions in MM environments within the *NVT* and *NPT* ensembles.

#### 4.2.11 Gao

A general approach that goes beyond QM/MM are the fragment-based methods.^624^ In 2013, we edited a special issue of *Accounts of Chemical Research* on this topic.^625^

Fragment-based methods such as the explicit polarization (X-Pol) model can be designed as general QM/QM embedding approaches in which each fragment can be individually represented by any electronic-structure method, with the inclusion of the instantaneous environmental effects through Hartree, Pauli exchange, and dispersion potentials.^626^ Importantly, X-Pol and other fragment-based methods provide a framework for the development of next-generation quantum-mechanics force fields (QMFFs) for condensed-phase and biomolecular simulations.^627^ In a QMFF, QM effects such as polarization, charge transfer, and the change of the potential-energy surface itself due to dynamical fluctuations as well as chemical reactions are naturally included. These effects would be very difficult, if not impossible, to describe using the current MM force fields.

### 4.3 In what areas of application are improvements needed?

#### 4.3.1 Helgaker, Teale, and Laestadius

Current DFT (CDFT), in which the density functional depends on both the charge density *ρ* and the paramagnetic component of the current density **j**_p_, was introduced in 1987 by Vignale and Rasolt.^628^ The initial works assumed a Hohenberg–Kohn-type theorem, but it was later recognized that no such theorem had been rigorously established.^36^ Moreover, the conclusion in ref. 36 was that the pair of densities *ρ* and **j**_p_ cannot determine the scalar and vector potentials *v* and **A** since a wave function can be the ground state of infinitely many systems when the flexibility of a vector potential is added. This observation rules out a Hohenberg–Kohn theorem for the paramagnetic current density. Regarding the total (as opposed to paramagnetic) current, no Hohenberg–Kohn-type theorem has so far been established but it is not precluded either since no counterexamples have been found.

Nevertheless, the relationship between *E*[*v*,**A**] and *F*[*ρ*,**j**_p_] is sufficient to establish the Vignale–Rasolt formulation as a rigorous extension of DFT to systems in an external magnetic field. To obtain a convex formulation, however, the change of variables *u* = *v* + |**A**|^2^/2 is needed, imposing a formulation of the theory where the potential space can absorb the norm squared of the vector potential.^629^ Such a formulation of CDFT inherits the mathematical structure of standard DFT, only lacking the uniqueness provided by a Hohenberg–Kohn result.

The lack of a Hohenberg–Kohn theorem for CDFT has led to confusion in the literature and some (erroneous) claims questioning the validity of the Vignale–Rasolt formulation – see ref. 630 and 631 for a discussion of these points. Aside from these controversies, questions regarding the mathematical properties of *F*[*ρ*,**j**_p_] remained unclear, particularly regarding whether a formulation analogous to Lieb's treatment of DFT could be established for CDFT.

Such a Legendre–Fenchel formulation of CDFT was developed in ref. 631–633 but the equivalence of the Vignale–Rasolt constrained-search functional and the Lieb-type functional was only very recently established with the proof of the lower semicontinuity and expectation-valued nature of *F*[*ρ*,**j**_p_].^634^ The expectation-valuedness is important for the AC since it allows the energy to be partitioned (into exchange and correlation parts) in terms of the minimizing density matrix (or wave function) that satisfies the density constraint.

The Kohn–Sham formulation of CDFT was introduced already in 1987^628,635^ and recently several practical implementations of this approach for general molecular systems using London atomic orbitals for gauge-origin independence have appeared.^636–638^ As CDFT becomes a more widely applicable and practical tool for molecular simulations, several open questions remain both from a theoretical and a numerical point of view:

##### Representability

For CDFT, representability issues may be more acute.^639,640^ To what extent may ensemble approaches play a role in this context?

##### Current-dependent functionals

Approximate current-dependent exchange–correlation functionals *E*_xc_[*ρ*,**j**_p_] are still in an early stage of development although some approaches have been presented for extending existing DFAs.^641–645^ Those based on meta-GGA functionals have shown some promise in strong fields.^39^ However, improvements for low-field properties such as NMR shieldings, central to chemistry, are more modest.^646^ What is the optimal gauge-invariant parameterization of *E*_xc_[*ρ*,**j**_p_]? How may new functionals be developed and tested?

##### Alternative formulations of DFT in a magnetic field

CDFT is not the only way to extend DFT to systems in a magnetic field. The magnetic DFT (BDFT) formulation of Grayce and Harris requires functionals of the form *E*[*v*;**B**] and *F*[*ρ*;**B**], which simplifies numerical implementation and avoids an explicit functional dependence on **j**_p_, at the cost of losing some degree of universality.^647,648^ Could the simplifications outweigh the loss of universality in practical implementations? Another alternative would be to consider the coupling of internal magnetic fields with the external field *via* a Maxwell–Kohn–Sham approach, which does feature a Hohenberg–Kohn result for the total current density.^649^ Such an extension may be important in strong-field time-dependent light–matter interactions^650^ and leads to a more appealing functional dependence on the total current density, rather than only on its paramagnetic component. Finally, we mention linear-vector-potential DFT (LDFT), a simplified formulation of CDFT suitable for uniform magnetic fields.^651^

Given recent strides in better understanding the theoretical foundations of CDFT, and the construction of several practical implementations, addressing these challenges should lead to further progress and improvements in the accuracy of magnetic response properties with DFAs.

#### 4.3.2 Görling

As pointed out in contribution (4.3.1), the development of approximate exchange–correlation functionals in CDFT is not an easy task and is still in its early stage. It is, however, possible to the treat the exchange contribution to these functionals exactly by generalizing the OEP method to spin-current DFT.^652^ By this generalization, exact exchange vector potentials coupling to density currents and exact exchange magnetic fields coupling to noncollinear spin components arise (in addition to the usual exchange potential coupling to the electron density) and can actually be calculated.

#### 4.3.3 Tellgren

I agree with contribution (4.3.1) and want to elaborate that the development of practical CDFT functionals has only reached a crude stage of development compared with conventional DFT functionals. Pure CDFT functionals should depend only on the density and the paramagnetic vorticity ***ν*** = **∇** × *ρ*^−1^**j**_p_.^628^

At least for molecular systems, pure functionals are not yet practically useful. Instead, the more pragmatic meta-GGA functionals are presently much better at capturing the response to magnetic fields. However, recent work has shown that the kinetic energy density employed in these meta-GGA functionals only builds in the correct gauge correction, but not any vorticity dependence.

One way forward is to employ a local tensor, akin to a stress-energy tensor, that encodes both the vorticity and a gauge-invariant kinetic energy in a natural way;^653^ see also the current dependent meta-GGA form in ref. 654. This tensor furthermore obeys strong *N*-representability conditions that enable discrimination of regions with one, two, three, and four-or-more Kohn–Sham orbitals. The isoorbital indicators that underlie many standard approximations discriminate rigorously only between one and two-or-more orbitals.

#### 4.3.4 Vignale

Following up on contributions (4.3.1) and (4.3.2), I would say that all generalizations of the original DFT of Hohenberg, Kohn, and Sham require a firmer mathematical basis. The absence of a strict Hohenberg–Kohn theorem is a problem not only for CDFT but also for spin-DFT, and, probably, also for DFT of superconductors.

Fortunately, there are many indications that the “nonuniqueness” of the potential is harmless for Kohn–Sham applications because the wave function remains unique even when the potential is not. However, I do feel that all multivariable DFTs have another hidden problem – namely, to what extent can the intensive variables of the theory be varied arbitrarily and independently of each other? This problem may be particularly severe when there are global constraints enforced by symmetry – for example, the total angular momentum of a rotationally symmetric system. Or there may be inequalities whereby the maximum value of a density (say, the spin density) can never exceed some maximum value that is controlled by another density (say, the particle density). Such constraints affect the intensive variables but not their conjugate fields, which can always be varied independently. This is one of the main reasons (if not the only one) why the map from densities to potentials is generally not invertible.

### 4.4 What extensions are needed to get ground- and excited-state properties and observables in DFT?

#### 4.4.1 Vignale and Ullrich

Interest in noncollinear spin magnetism in systems with strong spin–orbit coupling has greatly increased since the emergence of spintronics and the discovery of topological materials. The SU(2) formulation of spin DFT for a noncollinear spin density seems to be nearing a “phase transition” with the appearance of new DFAs. There exist several interesting ideas for the construction of noncollinear spin functionals using gauge-invariant blocks^655^ and orbital functionals:^656^ they should be pursued.

#### 4.4.2 Gidopoulos

The expectation value of any observable quantity is a functional of the ground-state density. As far as I am aware, there is little progress in developing exact or approximate functionals for general observables, except for the total energy and the density itself. The definition of the density functional of any observable is known – for example, if 
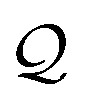
 is the operator for the observable *Q*, then the density functional *Q*[*ρ*] is given by70
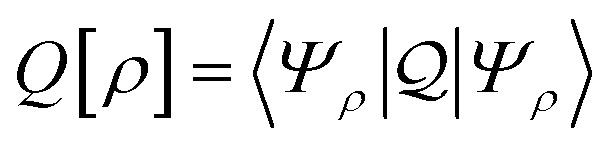
where *Ψ*_*ρ*_ is the minimizing state in the definition of the universal internal energy density functional71
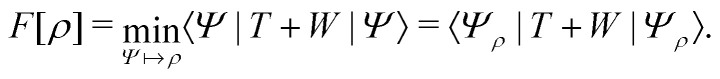
Since we do not have a good approximation to *Ψ*_*ρ*_, the Kohn–Sham state *Φ*_s_[*ρ*] is sometimes employed72
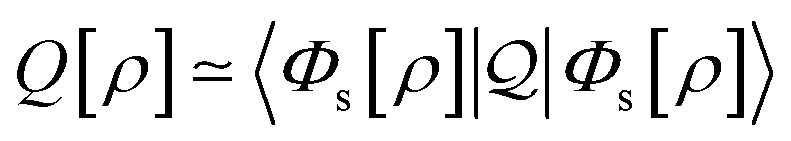
but this approximation is often not accurate enough.

Since the definition of the density functional of a general observable is known, the development of an approximate or exact density functional *Q*[*ρ*] means trying to find an exact or approximate expression that does not depend on the unknown interacting state *Ψ*_*ρ*_, but which depends only on the density and also on quantities that can be obtained from a Kohn–Sham calculation (*e.g.*, the Kohn–Sham state, orbitals, and eigenvalues) and which is more accurate than the obvious approximation in eqn (72). Recently, we managed to write down such a density functional for the magnetization density in DFT (not spin DFT) for open-shell systems in the absence of an external magnetic field.^657^

#### 4.4.3 Gould

Much recent work on ensemble DFT (see Section 3.7) and DFAs is focused on excited states.^421,422^ Ensemble DFT has the useful feature that differentiation with respect to ensemble weights gives access to Kohn–Sham wave functions (which can be multireference,^427^) densities, and energies of excited states.^430^ It has already been shown that ensemble DFAs can outperform their DFT or TDDFT counterparts in some difficult cases.^442,658^ Moreover, ensemble DFT can do so without breaking any symmetries^424,443^ and thus preserves spectroscopic features such as degeneracies.

#### 4.4.4 Maitra and Ullrich

Ensemble DFT is an elegant way to obtain excitation energies; however, we do not know how to obtain oscillator strengths from it. A more versatile approach to the calculation of spectroscopic properties such as optical spectra, excited-state forces, excited-state dipole moments and transition-dipole moments is *via* TDDFT.^97,659–661^ More than that, TDDFT gives access to a wealth of properties and observables in the nonlinear and real-time regimes, including high-harmonic generation or transient absorption, and, in general, electron dynamics on the attosecond time-scale. TDDFT can also be coupled with ionic dynamics, allowing practical calculations of the photochemistry of complex systems in mixed quantum–classical approaches. Needless to say, all these applications, while based on a theory that is in principle exact, involve approximations.

TDDFT has been overwhelmingly successful for a wide range of excited-state properties, but there are also spectacular failures. In the linear response regime, for example, it is now well understood that caution should exercised when standard DFAs are used to study states of double-excitation or charge-transfer character, and that these standard DFAs fail to yield excitonic spectra of semiconductors. However, such failures provide us with an opportunity to learn and improve our DFAs – much work remains to be done, but progress has been steady. It is also worth noting that the effort is well spent, given that TDDFT computations have a far smaller carbon footprint than alternative methods.

#### 4.4.5 Romaniello

Jacob and collaborators have recently proposed a scheme to extract the many-body spectral function of an interacting many-electron system from an equilibrium DFT calculation.^662,663^ This has been achieved by using an extension of DFT, called steady-state DFT (i-DFT).^664^

#### 4.4.6 Jensen

Excitation energies and transition properties may also be calculated using the long-range MCSCF – short-range DFT (MC–srDFT) method, which offers improved accuracy compared to TDDFT for ground states characterized by significant long-range nondynamical correlation and excited states with double-excitation character.^500,501^ The possibility of performing a state-specific optimization of an excited state with the MC–srDFT method offers another direct path to modelling excited states of any spin symmetry and any spatial symmetry; see contribution (4.1.3). Improvements to currently available short-range DFAs are needed to model the spin densities accurately, most likely by using the on-top pair density.

#### 4.4.7 Romaniello

An elegant but not often used (I do not know why) method for calculating the linear response of finite and extended systems is TD-current-DFT (TDCDFT).^665–667^ In TDCDFT, the basic quantity is the total current–density of the system, rather than the density as in TDDFT, that has a one-to-one mapping with an external vector potential. There are three main reasons for using TDCDFT:

1. for extended systems, it allows a well-defined expression for the macroscopic polarization of the system in terms of the induced current–density in the bulk;^668,669^

2. it allows one to treat the response to transverse fields;^670,671^

3. instead of looking for frequency-dependent approximations to the exchange–correlation kernel that are nonlocal functionals of the density (as done in TDDFT), one can look for consistent frequency-dependent approximations that are local functionals of the current–density, such as the Vignale–Kohn (VK) functional.^672^

Recently, Berger proposed a functional in the context of TDCDFT that can describe excitons in 3D materials.^673^ Linear response of 2D materials by contrast remains a challenge in TD(C)DFT.^674^

#### 4.4.8 Savin

As recalled in contribution (2.2.24), the universal functional, *F*[*ρ*] does not depend only on the density, but also on the Hamiltonian used. Bauer added operators to the Hamiltonian and, by exploiting the Hellmann–Feynman theorem, showed that properties can be obtained in this manner (even if not only the density is needed to get the expectation value of the operator).^675^ Bauer's approach requires density functionals that are specific for each property and new, property-specific DFAs must therefore be generated – see, for example, ref. 676.

This approach of DFT to molecular properties is not only interesting from a fundamental point of view; it can also show how far the present ideas for generating approximations can be taken – or help us understand why our current ideas work for the energy but not for a given property.

#### 4.4.9 De Proft and Geerlings

In conceptual DFT, the extension to excited states should certainly also be considered for use in, for example, photochemical reactions; see also Section 4.8. Its status and prospects have recently been commented on in ref. 475.

### 4.5 How can DFT further benefit from rigorous developments?

#### 4.5.1 Chattaraj

As systematic improvement is not possible in DFT (unlike in *ab initio* wave-function theory), research on fundamental aspects should continue. To achieve the goal of chemical accuracy in DFT, different approaches should be pursued side-by-side/in parallel, including the development of improved parameter-free functionals.

#### 4.5.2 Lewin

Rigorous mathematical results have played an important role in DFT. The most celebrated work is that of Elliott Lieb from 1983, who introduced the correct functional analysis setting for the ground-state problem.^8^ Several exact constraints have also been found, which could then be used in the construction of nonempirical DFAs, as mentioned by Perdew in contribution (3.1.2). This includes, for instance, the Lieb–Oxford bound,^677^ which provides an exact lower bound on the smallest possible Coulomb energy of *N* electrons, expressed only in terms of their density *ρ*. A recent review of known rigorous results for DFT can be found in ref. 33.

Several mathematical problems are still open and it would be nice to discuss here which of those could have an impact in DFT. I will only mention three problems which, in my opinion, deserve attention in the future.

The first is to better understand the Kohn–Sham potential. We have no rigorous proof that *v*_s_, which appears in eqn (8) and (32), for example, exists and, to my knowledge, no efficient numerical tool to construct an approximate one exists. Let me try to be a bit more precise.

Recall that a density *ρ* is (ensemble) *v*-representable when it arises from an *N*-electron (mixed) ground state with an external potential *v*. Let me emphasize that there are two notions of *v*-representability, for the interacting and noninteracting cases, respectively. The question is whether a *v*-representable density (for the interacting system) is *v*-representable by the noninteracting system. In other words, we need to study the set of densities that are simultaneously v-representable for the two cases. At the moment, nothing is known rigorously about this set, to my knowledge – in principle, it could even be empty! Of course, to properly discuss this problem, it is important to first fix a class of admissible potentials *v* that we wish to consider in DFT. This class should be large in order to increase the probability of being *v*-representable, but probably not too crazy either. Lieb considered all potentials belonging to *L*^3/2^(ℝ^3^) + *L*^∞^(ℝ^3^) because the energy is always bounded-below for such potentials.^8^ However, many physical cases do not appear in this class, such as the harmonic potential for instance. This is definitely a too small class.

One can understand *v*-representability in several other equivalent ways, all described by Lieb.^8^ My preference goes towards the Legendre–Frenchel point of view, which requires the use of mixed states and ensemble *v*-representability and was already discussed above in contributions (2.1.13) and (3.7.2). We know that the corresponding lowest total energy and the lowest kinetic energy for a given density *ρ* satisfy the duality principles^8^73
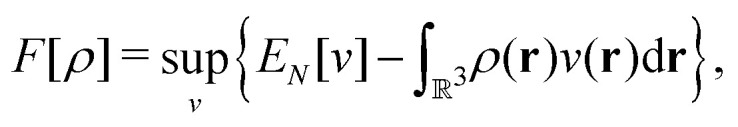
74
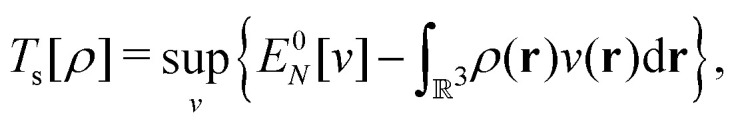
where *E*_*N*_[*v*] and *E*^0^_*N*_[*v*] are the interacting and noninteracting ground-state energies in an external potential *v*, respectively. Again, one should specify the set of potentials *v* in the two suprema, but any reasonable class will yield the same final value. The question is whether these suprema are attained (our desired potential *v*_s_ is a maximizer for *T*_s_[*ρ*]) and then the chances that this happens are much higher if the allowed class of potentials *v* is larger. Let me recall in passing that the existence of a dual potential is well understood in classical DFT. At zero temperature this follows from methods in multimarginal optimal transportation.^678^ At positive temperature, this result was proved by Chayes, Chayes, and Lieb.^679^ The quantum kinetic energy is thus the main obstacle here. Discretized quantum systems are studied in ref. 34, 680 and 681.

Another point of view has been mentioned above in contributions (2.2.2) and (3.7.2) and involves a kind of differentiability of *F*[*ρ*] and *T*_s_[*ρ*]. Any potential solving a maximum principle such as in eqn (74) is, formally at least, a derivative of the corresponding functional.^8^ To be able to treat the difference *F*[*ρ*] − *T*_s_[*ρ*], we thus need both to be differentiable at the same time. Although the notion of differentiability looks natural and intuitive, it is in fact not so easy. The reason is that the natural domain of *F*[*ρ*] is the set of densities with a finite von Weizäcker energy,^8^ which is not such a nice set.

Lieb proved that the two sets of *v*-representable densities are dense in the space *L*^1^(ℝ^3^) ∩ *L*^3^(ℝ^3^).^8^ The problem is that a dense set can, in principle, be extremely small – think of the rational numbers, which are dense but form a set of zero measure in the set of real numbers. Even worse, we need to look at the intersection of these two dense sets, which can be arbitrarily small or even empty. Very little is thus known mathematically about this problem.^681^

Let me now quickly mention the other two problems I had in mind. The second one is to improve existing exact constraints. For instance, I already mentioned the Lieb–Oxford bound,^677^ of which the best constant is believed to be that of the uniform electron gas.^177,682,683^ But at present, we have no idea on how to justify this rigorously.

Finally, I would like to mention that, unlike ground-state DFT, TDDFT is very poorly understood mathematically.^684^

#### 4.5.3 Kvaal

Moreau–Yosida regularized Kohn–Sham theory does not suffer from the nondifferentiability of *F*[*ρ*] − *T*_s_[*ρ*] and the problem of nonrepresentability therefore does not arise; see remarks in contribution (4.5.6). Also, Lammert has made an interesting attempt to coarse-grain exact DFT, where this issue is to a large extent resolved.^34^

#### 4.5.4 Laestadius

An important work addressing differentiability of the density functional *F*[*ρ*] is Lammert's work in ref. 685. Lammert provides a counterexample of a convex and lower semicontinuous function with a (unique) subdifferential that is not differentiable. Thus, this illustrates that convexity and lower semicontinuity are not enough to establish “*F*′ = −*v*”, even for variations that stay within the domain of the density functional *F*[*ρ*] – that is, Lieb's set of *N*-representable densities ℐ_*N*_.

#### 4.5.5 Helgaker

With regard to contribution (4.5.4), subdifferentiability of *F* on a dense subset of the *N*-representable densities ℐ_*N*_ (namely, on the set of ensemble *v*-representable densities ℬ_*N*_) follows from the convexity and lower semicontinuity of the universal density functional *F*. It is a general result of convex analysis that a proper lower semicontinuous convex function (here *F*) is subdifferentiable on a dense subset (here ℬ_*N*_) of its effective domain (here ℐ_*N*_) and everywhere in the interior of its effective domain (here the empty set since ℐ_*N*_ has no interior).

#### 4.5.6 Helgaker, Teale, Laestadius, and Kvaal

As pointed out in contribution (4.5.2), a central problem of DFT is the representability problem of Kohn–Sham theory. In short, what one would like to have is a simultaneous solution to the interacting and noninteracting Euler–Lagrange equations:75
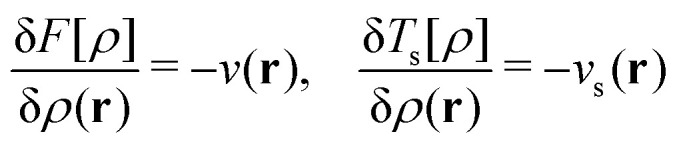
where *ρ* is simultaneously the ground-state density of the interacting system in the external potential *v* and the ground-state density of the noninteracting system in the external potential *v*_s_. The problem is that both *F* and *T*_s_ are everywhere discontinuous and therefore not differentiable^685^ – more precisely, they are subdifferentiable but only on a (dense) subset of their domains^8^ and there is no reason to believe that these subsets are the same for the interacting and noninteracting problems. In short, we cannot hope to find a Kohn–Sham noninteracting system with exactly the same ground-state density as the interacting system.

However, imagine that we change the ground-state energy in the manner
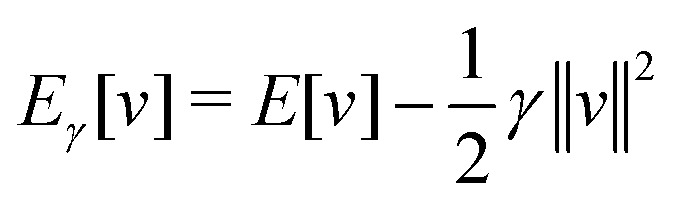
where *γ* > 0 can be arbitrarily small.^686^ The density functional then becomes
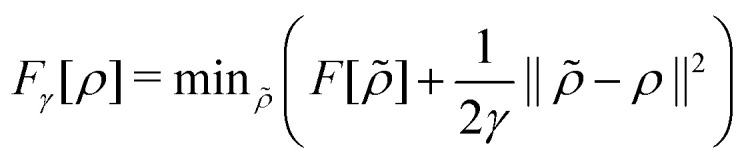
and likewise for (*T*_s_)_*γ*_. Importantly, *F*_*γ*_ and (*T*_s_)_*γ*_ are both everywhere differentiable, meaning that the interacting and noninteracting Euler–Lagrange equations can now be solved simultaneously.^99,686^ In convex analysis, such a procedure is known as Moreau–Yosida regularization.^687^ Once the regularized energy *E*_*γ*_[*v*] has been calculated, it is trivial to obtain *E*[*v*] and nothing is lost – that is, the Moreau–Yosida regularization of *F* has the curious property of being lossless with respect to the calculation of the ground-state energy *E*[*v*]. The only caveat is that ‖*v*‖ must be finite, which is only satisfied for Coulomb potentials by constraining the system to an arbitrarily large box.

In the regularized setting, every density is both interacting and noninteracting representable – a rigorous exact Kohn–Sham theory is thereby established. However, such a density need not be a “physical” density.

The Moreau–Yosida regularization of DFT may also be of practical interest as a tool for guaranteeing and improving convergence of the Kohn–Sham iterations.^99,688^

#### 4.5.7 Laestadius

Representability of a given density *ρ* can also be understood as the Lieb functional 
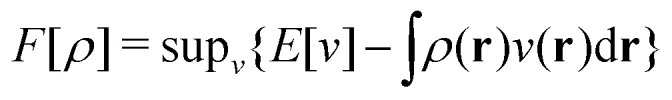
 (see contribution (2.1.13)) attaining its maximum, such that *ρ* is representable by its maximizing potential. In analogy with the above discussion of the Moreau-Yosida procedure, a maximizing potential can here be guaranteed by a regularization of 
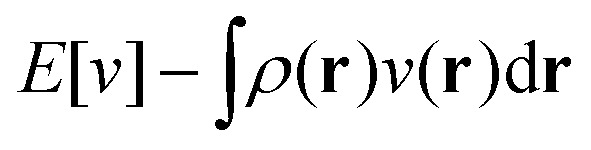
 using fixed weight functions *α*_*i*_ ∈ *L*^∞^. Such a scheme only makes use of partial information of the density constraint, *i.e.*, 
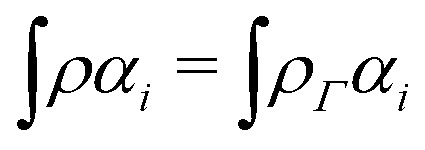
, for all *i* (for more details see ref. 689).

#### 4.5.8 Gori-Giorgi

The *ℏ* → 0 limit of the Levy–Lieb functional (see contribution (2.4.5)) establishes a link^690,691^ between DFT and the mathematical field of optimal transport; see, for example, ref. 678. When *ℏ* → 0, the Levy–Lieb functional tends to the SCE functional^139,140^76
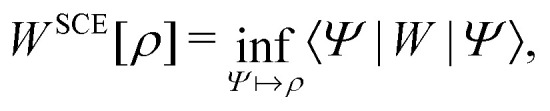
which defines an optimal-transport multimarginal problem with the repulsive Coulomb interaction as cost function.^690–692^ Techniques from optimal transport made it possible, for example, to prove^693^ that the exact SCE functional for one-dimensional systems is provided by the solution first guessed by Seidl^141^ on physical grounds. Another application is on the Lieb–Oxford bound (see contribution (4.5.2)), where optimal-transport methods can be used to improve bounds on the optimal constant.^694,695^

Some open questions that remain on the rigorous side concern the next leading term in the *ℏ* → 0 expansion, whose form was conjectured in ref. 144. A first step in this direction has been recently taken.^696^ I do have the feeling that there is still a lot to learn from the connection with optimal transport and by further analysing this limit, which provides complementary information with respect to perturbation theory. Although it is a semiclassical limit, it may perhaps be corrected for exchange (spin) effects,^697^ and could be combined with Kohn–Sham DFT.^408,698^

#### 4.5.9 Lewin

I fully agree with contribution (4.5.8). Related to the above discussion in contribution (4.5.2), one interesting question could be to understand what can be said about the Kohn–Sham potential *v*_s_ in the limit *ℏ* → 0. As I have mentioned, all densities are *v*-representable in the SCE limit eqn (76). So, more *v*-representable densities seem to exist when *ℏ* gets sufficiently small.

#### 4.5.10 Gori-Giorgi

A first (brute-force) attempt to get insight into this intriguing question has been made by Grossi *et al.*,^699^ who explicitly computed the functional derivative of the next leading term^144^ in the *ℏ* → 0 expansion, in the special one-dimensional case (for which we now know^696^ that the functional proposed in ref. 144 is exact). However, Kohn–Sham self-consistent calculations that include the functional derivative of this next leading term^700^ make the density poorer relative to the bare Kohn–Sham SCE result – even for very low-density systems, where inclusion of this term improves the energy. This suggests that this is not the right route to take to answer the question on *v*_s_, or at least that we need to better understand the expansion.

#### 4.5.11 Arbuznikov and Kaupp

Regarding the discussion in contribution (4.5.2), we would like to make a point about the difference between global and local exact constraints. Most of the extremely important and useful known and proven exact constraints pertain to global (integral) energy functionals. Yet most DFAs are designed with local (exchange and/or correlation) energy densities in mind. Apart from the issue of the nonuniqueness of any energy density (only defined up to a real-space function whose integral vanishes), constructions often apply known “global” constraints locally, even though the local versions of the constraints are usually ill-defined or unknown.

A point in case is the Lieb–Oxford bound.^677,695^ Becke argued that any reasonable exchange functional globally satisfies the Lieb–Oxford bound for any real chemical or physical system, irrespective of whether the underlying energy density obeys or violates it locally.^701^ In other words, a local Lieb–Oxford bound seems to be a sufficient but not a necessary constraint in the design of functionals – the exact-exchange energy density, in particular, violates the local Lieb–Oxford bound in the tail of any finite system. A local enforcement of the Lieb–Oxford bound gives enhancement factors of some widely used semilocal exchange functionals (*e.g.*, PBE^234^ and SCAN^235^ functionals) that are somewhat too low to describe finite systems adequately (in combination with an appropriate correlation functional), thus hampering thermochemical accuracy.

#### 4.5.12 Vignale

Concerning the mathematical foundation of TDDFT, it seems to me that much progress has been made recently by Ruggenthaler and coworkers in establishing the existence and uniqueness of the density-potential map; see ref. 98 for a review. This goes significantly beyond the original Runge–Gross theorem. There are more radical forms of TDDFT (*e.g.*, time-dependent DFT for the calculation of thermal currents, reviewed in ref. 702) that still lack a rigorous mathematical foundation.

#### 4.5.13 Ullrich

To follow up on contribution (4.5.12): the requirements for proving a rigorous mathematical structure of TDDFT are vastly different from those of ground-state DFT. In recent years, a consensus seems to have developed that the most promising avenue is to find a fixed-point proof,^98^*via* the force-balance equation. The latter is an equation of motion for the density, involving its second time derivative 
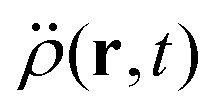
, external forces, and internal kinetic and many-body stresses. The fixed-point technique is mathematically very difficult and the TDDFT proofs based on it are still not fully rigorous.^98^

It has recently been shown that TDDFT can be reformulated using 
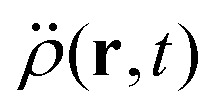
 instead of *ρ*(**r**, *t*) as the basic variable, which has the advantage that the causal structure of the theory becomes more transparent.^703^ This result may provide a new way forward in the ongoing attempts to solidify the foundations of TDDFT.

### 4.6 How can DFT further benefit from numerical analysis and algorithmic developments?

#### 4.6.1 Johnson

In finite-molecule calculations, use of hybrid functionals is routine. However, for plane-wave DFT calculations on periodic solids, use of hybrid functionals remains prohibitively expensive for most systems. A more efficient algorithm for evaluation of the exact exchange energy in plane-wave codes would benefit the application of hybrid DFAs to solid-state systems. Algorithms for efficient evaluation of the exact exchange-energy density in both finite-molecule and periodic-boundary codes would also aid the implementation of local hybrid functionals^319^ and of Becke's real-space correlation functionals.^13^

#### 4.6.2 Kronik

The overwhelming majority of DFT-based calculations are performed using either atom-centred basis sets or plane waves. While calculations based on a real-space grids have been available for a long time,^704,705^ their importance can be expected to increase – first and foremost, since this approach lends itself easily to massive parallelization across a large number of processors.

#### 4.6.3 Cancès

I agree. This also applies to finite-element^706^ and wavelet^707^ discretization methods.

#### 4.6.4 Cancès

Efficient SCF algorithms are available for a variety of systems of practical interest.^708,709^ However, SCF convergence remains problematic in some cases – for instance, for large, heterogeneous, systems such as metal-insulator interfaces. Progress has been made recently,^710^ based on a better understanding of the mathematical properties of the Kohn–Sham model. Some particularly difficult systems are still resisting, motivating further work in this direction.

#### 4.6.5 Cancès

Another numerical issue encountered in materials science, as well as in chemistry in the liquid phase (with explicit solvent molecules), is the choice of suitable supercells. The smaller the supercell, the lower the computational cost. On the other hand, the supercell must be large enough to limit spurious interactions from the artificial periodic boundary conditions. Finite-size corrections for point defects in periodic crystals have been proposed in the physics literature^711^ and analysed mathematically.^712^ Selecting optimal supercells and associated random configurations for disordered systems (alloys, glassy materials, and liquids) is a notoriously difficult problem.

Let us emphasize that the apparently simple case of a genuine, periodic crystal can be challenging also when the crystal is a metal. Recall that, for periodic crystals, using a supercell is mathematically equivalent to sampling the Brillouin zone with the regular *k*-point grid;^713^ the advantage of the latter approach is that it is far more efficient from a computational viewpoint. In most calculations, a relatively coarse *k*-point grid is used to further reduce the computational burden (say, 3 × 3 × 3 for insulators and 7 × 7 × 7 for metals). This approach is usually sufficient for insulators because the integrands are periodic, analytic, and weakly oscillating over the Brillouin zone for all relevant physical observables, but far from sufficient^714^ for metals with complicated Fermi surfaces. Smearing techniques^715^ at a fictitious positive temperature (possibly higher than the melting temperature of the metal) help to some extent,^716^ but do not fully solve the problem. It appears that many computational results on metals reported in the literature cannot be considered as converged with respect to *k*-point discretization.

#### 4.6.6 Galli

To enable first-principles MD with hybrid functionals for thousands of atoms and for time scales on the order of nanoseconds, algorithmic developments that reduce the scaling of the solution of the Kohn–Sham equations are needed. Such developments are also required for the derivation of deep-MD potentials based on the acquisition of DFT data for many configurations and under many different thermodynamic conditions.^717^

Many groups have worked on the development of 𝒪(*N*) techniques, from the early nineties up to very recently. Nevertheless, robust 𝒪(*N*) techniques for first-principles MD, where energies can be evaluated with a controlled error, are not yet available. Based on the experience acquired in the literature with 𝒪(*N*) methods implemented using plane waves, wavelets, or other localized basis sets, it appears that methods with controllable accuracy might come from the development of real-space based techniques, which would also require the development of specific pseudopotentials for periodic DFT calculations.^718^

#### 4.6.7 Cancès

What is the error in the output of DFT codes relative to the exact value of a chemical or physical quantity of interest (*e.g.*, the dissociation energy of a molecule, the bulk modulus of a material)? This question is obviously of major importance and is usually addressed by comparing experimental and computational results on large databases. However, such statistical analyses do not really answer legitimate questions of most users, which can be formulated as follows: “What will be the error for the specific system I am interested in if I use this code, with these numerical parameters (basis set/energy cutoff, convergence thresholds, *etc.*)? How should these parameters be chosen to obtain the accuracy I need, at the lowest computational cost?”

Providing partial answers to these questions is the purpose of a field of applied mathematics called *a posteriori* error analysis. This field has reached its maturity in, for example, finite-element based computational mechanics, where most academic and commercial codes provide numerical results (*e.g.*, the lift and drag of an aircraft) complemented by error bars. To understand what can or cannot be done in this direction for DFT, it is useful to decompose the overall error in several pieces:

1. the model error, coming from replacing the reference very accurate model (the *N*-body Schrödinger model or one of its relativistic counterparts) by a DFT approximation (LDA, PBE, B3LYP, *etc.*), possibly with pseudopotentials;

2. the discretization error due to the use of a finite basis set;

3. the algorithmic error due to finite convergence thresholds;

4. the finite-arithmetic error (computations are usually done in double precision);

5. execution error (negligible for current computers but an issue for future exascale^719,720^ and quantum computers).

It is already possible to estimate the discretization, algorithmic, and finite-arithmetic errors for linear Schrödinger equations discretized in plane-wave basis sets.^721,722^ The more recently developed error estimators are

1. guaranteed: mathematical theorems prove that the exact value indeed lies in the confidence interval;

2. accurate: the actual error is of the same order of magnitude as the error bar;

3. cheap to compute: evaluating the error bars requires only a moderate computational extra cost; and

4. systematically improvable: provide detailed information on how to increase the accuracy at the lowest cost.

Extending these techniques to the nonlinear Schrödinger and Kohn–Sham equations is work in progress.^723^ Such estimators would allow the computer program to choose adaptively, in a black-box manner, the best numerical parameters to reach a given numerical accuracy at the lowest computational cost (error balancing). Error balancing would be particularly useful for building large databases for ML, requiring hundreds of millions of single-point DFT calculations.^724^

Let us finally discuss the model error. For wave-function methods, it is in principle possible to estimate this component of the error by a careful mathematical analysis of the residual *H*_*N*_*Ψ*^app^_*N*_ − *E*_*N*_*Ψ*^app^_*N*_, where *H*_*N*_ is the *N*-electron Hamiltonian, and *Ψ*^app^_*N*_ and *E*_*N*_^app^ are the computed approximations to the ground-state wave function and energy; this is a topic of ongoing research. In the DFT setting, a promising approach is the use of (non-guaranteed) estimates based on a statistical analysis of the model error – see, for example, ref. 341–343 and 346. Whether nonstatistical, guaranteed, accurate, and cheap-to-compute model error estimators can be constructed using DFT is a completely open question.

### 4.7 What role will machine learning play in the future of DFAs and DFT?

#### 4.7.1 Scheffler

Artificial intelligence (AI) accepts that there are relationships or correlations that cannot be expressed in terms of a closed mathematical form. Thus, in principle, AI is more flexible than the theory of the past. The algorithm outlined in (2.1.4) “*ρ*(**r**) 
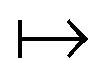
 ground-state energy (and even everything)” may be a case where an AI model can capture the relationship better than a mathematical functional. However, at present, the flexibility of AI comes together with a lack of interpretability, and the missing knowledge of the domain of applicability is probably an even more severe problem for AI models than it is for DFAs. AI can only predict aspects that were included in the training. If this is considered sufficient, then AI is a numerically efficient approach, once the laborious training has been performed with appropriate care.

#### 4.7.2 Salahub

Recent progress in AI/ML has been so astounding that even the “old guard” need to be aware and beware. As far as DFT is concerned, ML can have a great impact from (at least) three perspectives: (1) improving DFAs, (2) accelerating DFT calculations, evaluation of potential-energy surfaces and (Born–Oppenheimer) MD or, catastrophically for DFT, (3) obviating the need for DFT if ML-accelerated wave-function calculations become “infinitely” efficient (of course the advantages of a (Kohn–Sham) orbital picture would remain). I think the next few years will be very dynamic on all of these fronts, and others. And we, DFT aficionados, should also be paying attention to advances in quantum computers.

#### 4.7.3 De Proft and Geerlings

ML may be a valuable tool to inspire researchers in conceptual DFT to look for possible relationships between reactivity and conceptual-DFT descriptors that are not obvious when a limited number of cases are explored “by hand”. Care should of course be taken that, in the end, the researcher comes to a point where the link proposed by the ML ansatz provides him/her with a reasonable insight into the problem.

#### 4.7.4 Grimme

The primary purpose of Kohn–Sham DFT for AI/ML approaches seems to be the efficient generation of the required huge amount of reasonably accurate reference data (mostly energy and forces).

#### 4.7.5 Chermette, Adamo, and Ciofini

AI will be also involved in the building of exchange–correlation functionals – for example, by following Perdew's approach of satisfying 17 theoretical constraints; see contribution (3.1.2). A paper in this vein by Kirkpatrick and Cohen appeared recently.^725^

#### 4.7.6 Galli

I would like to mention DFT-based deep-potential MD (DeePMD) by Robert Car and Weinan E's group in Princeton^717^ and machine learning dielectric screening for the simulation of excited state properties of molecules and materials,^726^ which may eventually be used also to derive dielectric-dependent hybrid DFAs.

#### 4.7.7 Trickey

It is important to scrutinize ML results to see if they actually match the DFT on which they are supposed to be founded. We recently found an example in which such a match does not occur – namely, the liquid–liquid phase transition of hydrogen^727^ (in which H_2_ dissociates to atomic H). A machine-learned potential (MLP) had been developed by Cheng *et al.*,^728^ primarily by training on small (mostly 108-atom) and some intermediate-sized (512 atom) AIMD-DFT calculations. The resulting MLP-AIMD simulations for systems of 1728 atoms has a qualitatively different continuous transition from the first-order transition found by all the prior AIMD-DFT simulations (which were on smaller systems). Supposedly, the MLP-AIMD allowed bigger systems, hence overcame finite-size effects in the earlier AIMD-DFT studies. So we redid the brute-force AIMD-DFT calculations not only for 512 atoms but also 1024 and 2048 atoms. Our results were consistent with the earlier AIMD-DFT ones, a first-order transition. They do not confirm the MLP-AIMD results. Something artefactual remains in the MLP such that it is not a faithful extrapolation of the AIMD-DFT potential. I suspect that there is much yet to learn about the limitations of ML regarding extrapolation toward the thermodynamic limit, particularly in the treatment of phase transitions. Scrutiny, if not outright scepticism, is warranted.

### 4.8 How should interpretive tools based on DFT evolve?

#### 4.8.1 Ayers, Chattaraj, Chermette, De Proft, Fuentealba, Geerlings, Liu, Vela, and Yang

In the case of conceptual DFT, the prospects and concomitant issues were summarized in the status paper^475^ mentioned in Section 3.9. Regarding first the interpretive aspect, some issues should clearly be communicated by the experts to the practising chemist using conceptual DFT. For example:

1. Is the use of a temperature-dependent version of conceptual DFT^729^ necessary to avoid the *E*(*N*) differentiability problem when considering temperatures typical for laboratory conditions in synthetic work (leaving high-temperature chemistry aside)?

2. Can the pros and cons of going from the canonical ensemble, with the associated *E*[*N*, *v*] functional, to the grand canonical ensemble, with a state function *Ω*[*μ*, *v*] that allows fluctuations in the number of electrons,^730^ be clarified for the practising chemist? See also contribution (2.4.1).

3. Can one expect that the perturbation series of *E*[*N*, *v*] of a given reactant upon interaction with a perturbing reaction partner (the second reactant) converges properly in most cases?

4. Can the proper use of the principles mentioned in contribution (3.9.1) be supported by delineating their domain of applicability – that is, by formulating the conditions under which a meaningful application of the different principles is possible?

5. Can potential pitfalls in using conceptual DFT when going from kinetic (reactivity) to thermodynamic (stability) reasoning be identified?

On the other hand, the theory is in need of extensions – for example:

1. the inclusion of new variables in the *E*[*N*, *v*] functional such as electric and magnetic fields, mechanical forces, pressure to cope with the increasing variety of reaction conditions encountered in present-day chemistry;^731,732^

2. a proper and possibly elegant inclusion of spin^733^ to extend the theory to transition metals, of crucial importance in catalysis, and to radical reactions;

3. a generally applicable extension to excited-state reactivity^482,734,735^ to deepen the insight into photochemical phenomena and, in the same vein, an extension to time-dependent conceptual DFT;^736^

4. a deeper connection between conceptual DFT and information theory and its density functionals^193,737^ and with the reaction-force ansatz when considering reaction mechanisms.^738^

Recognizing that the ultimate goal of conceptual DFT is prediction, it was realized that the one reactant approach on the basis of the above mentioned *E*[*N*, *v*] expansion should be scrutinized to identify what terms in the expansion of *E*[*N*, *v*] are most likely to be efficient for a given problem, thereby challenging chemical intuition. Characteristics of the second reactant most probably should be explicitly introduced at various levels of refinement for quantifying the Δ*N* and Δ*v* perturbations.^475^

#### 4.8.2 Gao

In contribution (3.7.11), I described MSDFT as a hybrid wave-function and DFT method in the context of ensemble DFT. The basis states used to generate the energies of the adiabatic states in the ensemble can be viewed as effective valence-bond configurations. These configurations, obtained either through fragmental block-localization or by local electronic excitations, correspond to well-defined Lewis resonance structures, whose variational optimization can be directly used for block-localized wave-function interaction energy-decomposition analysis (BLW-EDA) to provide a quantitative interpretation of DFT results, such as aromaticity, hyperconjugation, and the Dewar–Chatt–Duncanson σ-dative donation and π-backbonding in transition-metal complexes.^739^ Furthermore, these localized electronic structures can be used to define diabatic states by orthogonal projection,^740^ suitable for dynamics simulations of nonadiabatic processes, including electron transfer, excited-state energy transfer, and photochemical reactions.

Recently, a general approach was introduced for treating spin-coupling interactions of open-shell molecules by MSDFT.^458^ The TDF energies that determine spin coupling are obtained by enforcing the multiplet degeneracy of the *S* + 1 state in the *M*_S_ = *S* manifold. Spin-adapted configuration states were used as the active space in MSDFT calculations of core excitations of open-shell molecules.

#### 4.8.3 Ayers

While existing density-based tools can provide deep insight into chemical bonding, molecular electronic structure, and even the thermodynamic driving forces for chemical processes,^741^ there are still outstanding issues. I am not entirely convinced that there is any fully satisfactory definition of bond order, atomic partial charge, *etc.* I am not even convinced that the canonical Parr–Pearson definition of chemical hardness is the best one.^742^ Perhaps some of these concepts must be discarded. However, the power of DFT is that, unlike most (but not all) other approaches, it is mathematically rigorous and grounded on physical observables. This allows one to attempt to approach chemical concepts axiomatically: first one lists the key properties/tests one expects a concept to possess/satisfy, then one tries to find a mathematical definition.^743^

## 5 Communicating and sharing our results

### 5.1 How should the DFT community organize and share information?

#### 5.1.1 Loos

No one should have to code the B3LYP functional again: the democratization of open-source software and libraries such as libxc,^744^ xcfun,^745^ and numgrid,^746^ are a big step in this direction. The availability of the source code should be strongly encouraged, especially for research funded by public money.

#### 5.1.2 Krylov

I would like to distinguish between open sharing of ideas and basic tools useful for prototyping *versus* open-sourcing production-level codes. Yes, we should openly exchange ideas and share basic tools of development, or some libraries. But, as described in this Viewpoint article,^747^ sustainability of code development cannot be ensured by present funding models and license income then provides a way to sustain scientific developments and software maintenance. We should remember that our ultimate goal is to provide chemists with software that is robust, effective, and usable. The commercial software-development model provides a vehicle for achieving this goal.

#### 5.1.3 Trickey

Most of the issues associated with this section seem to be generic to the practice of scientific research and not specific to DFT. Those issues include the sociology of science, national funding policies (note Krylov's contribution (5.1.2)), priorities, and mandates (*e.g.*, data management plans in the USA), institutional practices and policies (*e.g.*, tenure and promotion in the USA compared to say China or Germany or México). Given that enormously variegated setting and given the sprawling utilization of many-fermion DFT in myriad diverse specialities and technologies, one might ask whether there are DFT-specific aspects of dissemination, communication, and/or data management, for which the DFT development community has explicit responsibilities and/or opportunities.

One such DFT-specific aspect has been pointed out by Loos in his contribution (5.1.1). But as usual there is a hitch. First, just because a DFA (or KEDF) is implemented for some kind of Gaussians, this doesn’t mean that it will work for a plane-wave PAW code. (It may not even work for another kind of Gaussians without some fiddling.) Secondly, there are mis-implementations. (The B3LYP DFA itself is a kind of mis-implementation and there are the notorious VWN versions.) Postdocs in my group have found several mis-implementations in popular codes and one of our finite-temperature DFAs was mis-implemented in libxc. Especially for intricate DFAs (and KEDFs), it seems healthier to have several independent implementations.

Another step would be for presentation of a new DFA to include an explicit, unambiguous statement near the outset of whether it was intended for only a certain class of molecules or only for molecules but not condensed phases, *etc.* and on what class of systems it had been tested.

#### 5.1.4 Draxl

I agree, for example, with contribution (5.1.1) that we should share as much as possible to avoid that many people are doing the same again and again. We should use our human resources for going beyond what is done already. This also implies, however, that many of us contribute to open-source libraries; testing and feedback is another issue. It is also good to have alternative implementations for comparison. Very important, we as a community also need to appreciate much more the work that some people are putting into developing codes and tools. It often happens that such work is considered “nonscientific” or “programming jobs only”. Also, when papers on implementations are submitted, referees reject these because of lack of novelty. People dedicating months and years to develop tools that are used by the community should not have a disadvantage when being considered (or not) for a job because they published less during this time.

#### 5.1.5 Ayers

I see no drawback to being aggressively open. Indeed, I believe we should aspire to share so aggressively that reproducing, and even extending, a study is not only possible but feasible. This requires more than FAIR sharing of content/data;^748^ it requires more than releasing open-source software; it requires a high standard of communication/documentation for theory, data, algorithms, and code. I concur with contribution (5.1.3) that some of these issues are very broad, and we can certainly learn from other researchers in the computational mathematical sciences.

#### 5.1.6 Savin

One aspect related to sharing information is improving the condensed information we share – for example, the way we summarize the results obtained from benchmarks. This means that we need good tools to analyse the existing and ever-increasing amount of data.

#### 5.1.7 Draxl

The need of benchmarks is also emphasized in contribution (5.2.3). A very first step was made by Lejaeghere *et al.* in a true community effort, known as the *Delta test*.^749^ From the beginning of the initiative to publication, it took several years during which codes and pseudopotentials were significantly improved. Still, this is only a very first step as this work only concerns total energies for elemental solids and a single semilocal exchange–correlation functional (PBE). We need comparative studies for very different properties (barriers, band gaps, spectra, *etc.*) and very different types of materials (organic, inorganic, surfaces, interfaces, hybrid materials, *etc.*), carried out on different levels of methodology. Even the *Delta test* data, though appearing extremely consistent across many codes with regard to the total energy, exhibits an unacceptable spread when it comes to properties, as revealed in a subsequent analysis.^750^

#### 5.1.8 Crawford

I fully agree that the broad dissemination of both established and emerging DFAs and related DFA technologies is to the benefit of the scientific community, and optimized libraries such as libxc,^751^ xcfun,^745^ and numgrid^746^ provide superb examples of the added value of such an approach. The impact of libxc, in particular, is noteworthy in that it provides more than 600 density functionals (LDA, GGA, and meta-GGA) to dozens of community quantum-chemistry and materials-science software packages, both open-source (*e.g.*, PySCF,^752^ Psi4,^753^ Quantum ESPRESSO^754^) and commercial (*e.g.*, ORCA,^755^ ADF,^756^ Molpro^757^). Furthermore, the library is applicable not only to Gaussian basis sets, but also to plane waves, adaptive grids, and finite-element representations.

Libraries and modules such as these not only provide high performance, but also improved reproducibility and standardization, both of which are becoming more vital as the complexity of our models advances. To that end, emerging standards and tools for sharing computational results will similarly grow in importance. In the materials-science domain, for example, this has long been underway with community-driven resources such as the Materials Project^758^ as a paradigm, although the standardization of the content of materials databases is still under development within that community. In the computational-chemistry domain, new tools such as the Quantum Chemistry Schema (QCSchema) and Quantum Chemistry Archive (QCArchive)^759^ (an open, public-facing database of computational results) developed by the Molecular Sciences Software Institute (MolSSI)^760,761^ would allow much greater interoperability between codes by facilitating standards for data sharing. In addition, the Simulation Environment for Atomistic and Molecular Modeling (SEAMM),^762^ also under development by the MolSSI, provides a lightweight, Python-based plug-in environment for complex, shareable workflows, which will permit sophisticated computations involving multiple community code components in a fully reproducible and publishable manner. The broader the adoption of standards and tools such as these, the faster our community will be equipped to handle more complex and important scientific challenges.

#### 5.1.9 Reining

Since data from models, such as the quantum Monte Carlo data for the homogeneous electron gas,^241^ have turned out to be so precious for DFT, we should think about the best way to share such data. Some of us think that models more complex than the homogeneous electron gas may contribute to better approximations,^242,763^ not only finite models with uniform electron density as Loos mentions in contribution (3.1.5), but truly inhomogeneous, still simple, systems. To tabulate and/or interpolate such model data will be hard work on its own, and sharing the results will be crucial.

### 5.2 How and what should we publish?

#### 5.2.1 Loos

Publishing negative results should be encouraged much more in our community because they may be as valuable as the positive ones and may provide important insights.

#### 5.2.2 Loos

Hopefully, the popularity of open-access repositories for electronic preprints and postprints (such as arXiv or ChemRxiv) will keep growing in our community so that researchers have rapid access to free, new science. I personally believe that the present model where researchers seek funding, supervise students/postdocs, write articles, and review them is a broken, unsustainable model.

#### 5.2.3 Draxl

I agree with both – we should change our publication culture, including also publication of negative results. But we also need more “positive results”. While in chemistry, verification/validation and benchmarking are a matter of course and have been for many years, or even decades, in computational physics this is still in a very early stage. All this is, however, crucial for assessing methodology and distinguishing between accuracy (of a method) and (numerical) precision.

#### 5.2.4 Jones

I have participated in countless discussions over many years concerning scientific publication, mostly in physics. Common conclusions have been that too much is published in too many journals, and improved refereeing is needed to reduce the number of publications whose quality is borderline or below. The world has gone in the opposite direction. Open access publication has some advantages, but it has contributed to the continuing proliferation of journals and can result in a lowering of standards (accepted papers bring income, rejected papers do not). The widespread use of electronic archives increases the number of articles that are not reviewed at all. I see little hope for change and have depended for years on private communications about new developments.

Identifying something as “broken” and/or “unsustainable” as in contribution (5.2.2) could be the first step towards repairing it, but I am not optimistic. I am reminded of the alleged response of a local in rural Ireland: “If I wanted to get to Dublin, I wouldn’t start here.” Ever-increasing pressure to obtain external funding will both hamper risk-taking and increase focus on “fashionable” topics.

#### 5.2.5 Savin

I agree with contribution (5.2.4). I feel drowned in the publication flood. Finally, the question is about transmitting information. Maybe we should try to establish ways to present essential findings that is incremental, and can be updated, in the style of Wikipedia. The numerical support could be put in a database that can be searched by automatic tools.

#### 5.2.6 Trickey

Again we are faced with generic challenges in physical science. Funding pressures are one. Competition among publishers to have the most exclusive journals is another. Emphasis is on the allegedly spectacular. These influences combine to make it hard to publish careful, incremental advances, let alone negative results. Within the DFT community, maybe we should urge editors to accept the publication (hence, also, respectful refereeing) of careful presentation of negative results about well-motivated, well-grounded attempts at advancement?

#### 5.2.7 Gori-Giorgi

We should also not forget that the pressure to publish goes hand in hand not only with the competition to obtain funding but also with how we evaluate (young) scientists. There is now (finally) an attempt to shift from criteria based on quantity (like the number of publications, the h-index, *etc.*) to move towards quality.^764^ How to define the latter is of course a big challenge – although experts usually recognize quality in their field, any definition has exceptions. There is also more focus on collaboration and team science, which are positive developments.^764^ But without appropriate funding and reasonable career perspectives for young people, the situation regarding overpublishing and overselling results (writing artificial success stories) will probably remain dire.

#### 5.2.8 Romaniello

I agree with contribution (5.2.4). In particular, open-access publication seemed a nice idea at the start but, as with most human activities where money circulates, the system got corrupted. The publishing fees are now so high that it is much cheaper for the scientific community to keep the standard subscription model. Moreover, nowadays everybody can wake up in the morning and create their own journal, which makes it difficult for the institutions to keep track of “serious” journals. I like the idea proposed in contribution (5.2.5), which puts back in the spotlight the importance of sharing knowledge and not of increasing the h-index.

#### 5.2.9 Galli

I would like to alert the community on one of our efforts to make data available on a per-publication basis, which could be used also for DFT publications. Please see Qresp, a tool for curating, discovering and exploring reproducible scientific papers.^765,766^ For an example of a curated paper, see ref. 767.

#### 5.2.10 Ayers

For traditional electronic-structure calculations on molecules and materials, there are existing platforms like QC-Archive^768^ and the NOMAD repository^769^ for securely storing and sharing data. These databases provide good search capabilities, support most popular electronic-structure packages, use the well-defined JSON schema, which can be directly accessed/used (especially NOMAD), and at least partly fulfil the goals of making data findable, accessible, interoperable, and reusable (FAIR).

### 5.3 What format should workshops and conferences take in the future?

#### 5.3.1 Maitra and Ullrich

In the past 15 years or so, there have been a number of schools and workshops geared towards graduate students and postdocs – notably, the DFT/TDDFT tutorials at the March Meeting of the American Physical Society, the biyearly TDDFT series at the Benasque Center for Science since 2004, a similar series in the US since 2017, and the CECAM workshops on learning the theory of DFT. Despite the positive feedback these events have received, their impact is limited to those who can travel to their locations. The Zoom activities that arose out of necessity during the pandemic (*e.g.*, the international PhD student seminar series on (TD)DFT theory development^770^) offer us the possibility to think about establishing hybrid schools and workshops routinely: not only to reduce our carbon footprint at the heights of the climate crisis, but also to enable students truly from all over the world to attend.

#### 5.3.2 Romaniello

We should not go back to the pre-pandemic model. In this last year, we have learned that we can easily follow a workshop/conference from our office/home. Of course, we also need real interactions, but we could select one or two events per year in which to participate in person and the rest online. Together with the advantages mentioned in contribution (5.3.1), let me add that the possibility of hybrid events will be beneficial also for female scientists just back from maternity leave, for whom it is usually complicated to leave home for several days.

#### 5.3.3 Reining

I would like to advance two more arguments in favour of using online tools in general: First, if we talk about family matters, this should concern not only women, but also, and equally, men who care for their family. Second, we are scientists and know about the climate – so, let us make an effort to travel less and shorter distances, preferably by train.

We should work out new formats that do not force us to choose between taking a plane and having coffee with colleagues, or just sitting in front of a screen. We could work out, for example, a delocalized physical conference, where smaller hubs are connected by internet and people can travel to the nearest hub. Such a format would necessitate new forms of discussion but, if we are not too conservative, we can certainly come up with solutions. Besides, online tools also allow us to make material available in advance, such that newcomers in the field can be better prepared and profit more.

### 5.4 How can we best teach and communicate DFT?

#### 5.4.1 Helgaker

I believe too much is made of the Hohenberg–Kohn theorem – expressing the ground-state energy as a function of the density alone in the manner *E*[*ρ*] is unhelpful and obfuscates the theory. We do not ever attempt to obtain the energy is this manner, by some miraculous use of the Hohenberg–Kohn theorem. The constrained-search approach is a much more intuitive and transparent introduction to DFT and the theory of Lieb provides an elegant mathematical framework that captures the essence of DFT.

#### 5.4.2 Jones

In my experience, lecture courses and seminars involving DFT usually give a rather boring view of its history. The world began in 1964 with Hohenberg–Kohn, Kohn–Sham made DFT usable, and so on. The listener learns nothing about the excitement people working in DFT experienced during the bleak years up to 1990, perhaps because the speaker does not know or care. Here are some points for consideration.

The Hohenberg–Kohn theorem^5^ is ubiquitous, but little used in practice. Its proper place today is in review articles and textbooks, and we should focus on the constrained-search approach^6^ and the formulation of DFT in terms of Legendre transforms.^8^ Kutzelnigg's “beginner's” guide to the latter is accessible to most in the DFT field.^771^

As noted in contribution (2.1.13), “the power of DFT derives from Kohn–Sham theory,” but the successes of Kohn–Sham theory are linked closely to the ability of local density approximations to the exchange–correlation energy *E*_xc_ to give useful results in most cases. Kohn noted: “I believe that formal DFT would have been of very little interest if there had not been a simple and very practical approximation for *E*_xc_, the LDA, which has yielded surprisingly accurate results.”^772^

Kohn and Sham proposed using an LDA for *E*_xc_ that is exact in two limits (slowly varying densities and high densities).^65^ These are far from the density distributions found in atoms, molecules, and condensed matter. Kohn and Sham, and many others, were therefore convinced that LDAs would not describe chemical bonding well. Nevertheless, they gave “reasonable” answers in early tests of energy differences (including small molecules^120,469,773^ and jellium half-spaces^100,119^) and remain the basis of many approximations for *E*_xc_. The initial successes of LDA were so surprising that they motivated work to understand why it could provide useful energy differences for systems with densities far from the regions of obvious validity. This work led to “adiabatic coupling” and studies of the exchange and correlation holes, their spherical averages and related sum rules,^100,119,120^ which have been of lasting value in DFT studies. This surprisingly satisfactory description of reality often provided by LDAs was essential to the ultimate success of DFT.

#### 5.4.3 Chattaraj

The density (its advantage over the wave function), density matrices (writing energy in terms of them), and density functionals (a map from a function to a number) should be introduced. DFT highlights the fact that, as *N* and *v*(**r**) fix the Hamiltonian, the Schrödinger equation is a map from these quantities to the density, whereas the corresponding inverse map (along with normalization) is DFT. Of course, Kato's cusp condition^4^ for the ground state of any system provides *v*(**r**) through nuclear positions and the charges, when the density is known. Various techniques (Kohn–Sham, Levy–Lieb, orbital-free DFT, *etc.*) exist for calculating the density. A recapitulation of Hartree–Fock theory may be helpful.

#### 5.4.4 Maitra and Ullrich

DFT is taught in many ways and at many levels: in addition to the tutorials, workshops, and summer schools mentioned in contribution (5.3.1), there are online courses,^774^ regular courses at universities, and pedagogical textbooks.^659,775–777^ The targeted audience often tends to be at an advanced level (graduate students, postdocs, researchers). However, there is an urgent need to teach DFT at a more basic, introductory level, to make it accessible to undergraduate students and to those who may not have a strong background in quantum mechanics, and who wish to understand and learn how to use DFT.

When teaching DFT to beginners, we face similar choices as in other fields of physics (*e.g.*, quantum mechanics, electrodynamics): we can follow the historical path in which the field was established, or we can start with the most fundamental theorems and then build up the formalism, or we can introduce the subject through examples, case studies, and hands-on applications. In our experience, students tend to learn DFT better when the latter approach is taken; if we start with the theorems or with many-body theory, students often fail to see the connection to the “real world”. Thus, as a community, we should make an effort to make DFT more accessible and inclusive, and to do this it will be helpful to develop (and share) simple numerical examples and hands-on exercises.

#### 5.4.5 Grimme

The title of this paper contains the word “workhorse” and hence we should not forget to teach this aspect – that DFT really works every day in thousands of applications. DFT is a theory that is generally robust but students should also know when it fails (rarely) and why and how this is related to the DFAs (and other) approximations involved. Therefore, it is especially important to teach the basics of DFT in the context of real-world applications to illustrate that scientists are able to use it as a versatile tool to solve chemical and physical problems in many disciplines. The perception of DFT as a valuable component of today's fundamental chemistry–physics method toolbox should be promoted accordingly, through practicals and lectures with a pronounced hands-on mentality.

#### 5.4.6 Gori-Giorgi

I fully agree with everything said above, especially on using the constrained-search approach instead of introducing the Hohenberg–Kohn theorem. Obstacles I often see students facing when trying to learn DFT are:

1. To understand the theory behind DFT, you need to have a good understanding of many-electron wave functions and reduced density matrices, especially in real space, which they often lack.

2. Most pedagogical material focuses on DFT without spin densities, while in applications the latter are used.

3. Modern exchange–correlation functionals are very complicated and look obscure to them.

4. The language is often ambiguous – for example, in the literature and in conferences the term “local” is sometimes used to indicate a multiplicative potential (the local Kohn–Sham potential, as opposed to the nonlocal Hartree–Fock potential) and sometimes to indicate a local dependence on the density.

5. The role of symmetry breaking is very important and often neglected in pedagogical material.

Finally, I believe it would be useful to teach LDA in a more modern way, by including the recent works on the large – *N* limit of neutral atoms, which show in which sense LDA is a universal limit for Coulombically bound systems and how gradient expansions arise; see, for example, ref. 179 for a recent review.

#### 5.4.7 Reining

Good teaching should help the learner to take a step back and understand the essential elements, rather than the technical details. A crucial question that is in my opinion often neglected but merits deep thought, is the motivation: Why should we choose to work with DFT? The reason cannot just be that so many people do it successfully. In other words, we should (in general, and also for DFT) talk more about how we make choices in science. The answer may include a historical component and should mention alternatives – not for a detailed comparison, but to highlight some basic choices that may distinguish or be common to different methods. For DFT, I would insist on the following points:

• We know in principle how to calculate observables in terms of many-body wave functions, but we cannot do it in practice in most cases. The choice of DFT, instead, is to express ground-state observables as functionals of the ground-state density. The density is an object that is more compact (depending on fewer variables) than the many-body wave function;

• We could also express observables as functionals of other quantities that are more compact than many-body wave functions – DFT uses the ground-state density, but we could also choose density matrices, for example, as a good descriptor;

• As a rule of thumb, the more compact we make the descriptor, the fewer observables can be calculated as explicit functionals. We can therefore discuss when and why the density is a convenient choice – for example, when the Hartree energy is important;

• The idea to use an auxiliary system to determine one or more, but perhaps not all, observables exactly should be exposed clearly;

• In many-body physics, we know from the very start that approximations will be needed. It is then crucial to discuss why DFT is a good starting point for approximations;

• We should also discuss the choice of strategies for developing approximations, rather than the technical details. One example in DFT is to calculate the kinetic energy from a noninteracting system with the same density as the interacting one. Another example is to use the intuitive concept of nearsightedness.^184,185^

These are very general ideas that can be found – maybe under different names – also in contexts other than DFT, but they have all been important for the success of DFT.

## Conflicts of interest

There are no conflicts to declare.

## Supplementary Material

## Appendices

### Appendices

#### A Acronyms

AC: Adiabatic connection
ACFD: Adiabatic-connection fluctuation-dissipation
ADFT: Auxiliary DFT
AI: Artificial intelligence
AIMD: *Ab initio* MD
AM1: Austin Model 1
BBGKY: Bogoliubov–Born–Green–Kirkwood–Yvon
BCB: Bottom of the conduction band
BDFT: Magnetic DFT
BLW: Block-localized wave function
BOMD: Born–Oppenheimer molecular dynamics
BSE: Bethe–Salpeter equation
CASPiDFT/CASΠDFT: CAS pair-density functional theory
CASSCF: Complete-active-space SCF
CASPT2: Complete-active-space PT2
CCSD: Coupled-cluster singles–doubles
CCSD(T): CCSD with perturbative triples
CDFT: Current DFT
CI: Configuration interaction
DC: Dirac–Coulomb
DD: Density driven
DEKS: Direct-energy Kohn–Sham
DF: Density functional
DFA: Density-functional approximation
DFET: Density-functional embedding theory
DFT: Density-functional theory
DFTB: Density-functional tight binding
DFT+*U*: DFT with the Hubbard *U* correction
DK: Douglas–Kroll
DMET: Density-matrix embedding theory
DMFT: Density-matrix functional theory
DMRG: Density-matrix renormalization group
dRPA: Direct RPA
EA: Electron affinity
ECW: Embedded correlated wave function
EDA: Energy decomposition analysis
EDFT: Ensemble DFT
EFG: Electric field gradient
ELS: Extended Löwdin–Shull
ELS +: Augmented ELS
EXX-OEP: Exact exchange – optimized effective potential
FCI: Full CI
FDET: Frozen-density embedding theory
FPMD: First-principles molecular dynamics
GASSCF: Generalized-active-space SCF
GGA: Generalized gradient approximation
GKS: Generalized Kohn–Sham
GL: Görling–Levy
GL2: Second-order GL
GMTKN55: General main-group thermochemistry, kinetics, and non-covalent interactions dataset
*GW*: Green's function *G* and screened Coulomb interaction *W*
GOEP: Generalized OEP
GOK: Gross–Oliveira–Kohn
HEG: Homogeneous electron gas
HF: Hartree–Fock
HK: Hohenberg–Kohn
HOMO: Highest occupied MO
Hxc: Hartree–exchange–correlation
IP: Ionization potential
i-DFT: DFT for steady-state transport
KE: Kinetic energy
KEDF: Kinetic-energy density functional
KS: Kohn–Sham
LFDFT: Ligand-field DFT
LD: Local density
LDA: Local density approximation
LH: Local hybrid
LOB: Lieb–Oxford bound
LR/lr: Long range
lrMCSCF–srDFT: Long-range MCSCF with short-range DFT
lrWFT–srDFT: Long-range WFT with short-range DFT
LUMO: Lowest occupied MO
MAE: Mean absolute error
MC: Multiconfigurational
MBPT: Many-body perturbation theory
MC-DFT: Multiconfiguration DFT
MC-NCFT: Multiconfiguration nonclassical functional theory
MC-PDFT: Multiconfiguration pair-density functional theory
MCSCF: Multiconfigurational SCF
MC-srDFT: MCSCF theory with short-range DFT
MD: Molecular dynamics
MD/MC: Molecular dynamics/Monte Carlo
MGCDB84: Main-group chemistry database with 84 data sets
ML: Machine learning
MLP: Machine-learned potential
MM: Molecular mechanics
MMRDFTP: Mild-mannered respectable DFT practitioner
MNDO: Modified neglect of diatomic overlap
MO: Molecular orbital
MP4: 4th-order Møller–Plesset
MSDFT: Multistate DFT
MSSCF: Multistate SCF
NEVPT2: *N*-Electron valence state PT2
NOSI: Nonorthogonal state interaction
NTO: Natural transition orbital
OEP: Optimized effective potential
OO: Orbital optimization
OT: Optimal transport
PAW: Projected augmented wave
PD: Pair density
PFET: Potential-functional embedding theory
PFT: Potential-functional theory
PM6: Parameterization Method 6
PPLB: Perdew–Parr–Levy–Balduz
PT2: Second-order perturbation theory
PZ: Perdew–Zunger
QCT: Quantum-chemical topology
QE-DFT: Quasi-energy DFT
QED: Quantum electrodynamics
QM: Quantum mechanics/mechanical
QMC: Quantum Monte Carlo
QMFF: Quantum-mechanical force field
QM/MM: Quantum-mechanics/molecular-mechanics
QM/QM: Quantum-mechanics/quantum-mechanics
QTAIM: Quantum theory of atoms in molecules
QZ: Quadruple zeta
RASSCF: Restricted-active-space SCF
RDF: Relativistic DF
RDM: Reduced density matrix
RHEG: Relativistic HEG
RPA: Random-phase approximation
RS: Range separation
SA-CASSCF: State-averaged CASSCF
SAOP: Statistical averaging of orbital potentials
SCE: Strictly-correlated electrons
SCF: Self-consistent field
SF-TDDFT: Spin-flip TDDFT
SIC: Self-interaction correction
SIE: Self-interaction error
SR/sr: Short range
srDFT: Short-range DFT
SSE: Sham–Schlüter equation
SU(*n*): Special unitary group of degree *n*
TAO-DFT: Thermally-assisted-occupied DFT
TB: Tight binding
TDCDFT: Time-dependent CDFT
TDDFT: Time-dependent DFT
TDDMFT: Time-dependent DMFT
TDF: Transition-density functional
TRIM: Trust-region image minimization
UEG: Uniform electron gas
vdW: van der Waals
WF: Wave function
WFT: Wave-function theory
XC: Exchange–correlation
X-Pol: Explicit polarization
X2C: Exact two-component
X*α*: X-alpha method
ZDO: Zero differential overlap
ΔSCF: Delta-SCF
1RDM: First-order reduced density matrix
2RDM: Second-order reduced density matrix

#### B Mathematical symbols

*A*: Electron affinity
ℬ_*N*_: Set of ensemble *v*-representable densities
*E*: Total electronic energy
*E*_H_: Hartree density functional
*E*_xc_: Exchange–correlation density functional
*E*_Hxc_: *E*_H_ + *E*_xc_
*F*: Universal density functional
*f*: Fukui function
*H*: Hamiltonian
*I*: Ionization potential
ℐ_*N*_: Set of *N*-representable densities
*j* ^*μ*^: Four-current-density
**j**: Total current-density
**j**_p_: Paramagnetic current-density
*L*: Angular momentum
*L* ^*p*^(ℝ^3^): Set of Lebesgue *p*-integrable functions on ℝ^3^
**m**: Magnetization density
*m*_e_: Electron mass
*N*: Number of electrons
**R**: Nuclear positions
**r**: Electron positions
*s*: Local softness
*S* ^2^: Total spin squared
*S*: Chemical softness
*t*: Time
*V*
*v*: External potential
*W*
*w*: Potential of interaction between electrons
*v*_s_: Kohn–Sham potential
*v*_xc_: Exchange–correlation potential
*T*: Kinetic energy
*T*_s_: Kinetic energy of the Kohn–Sham noninteracting system
*W*: Two-particle interaction
*Z*: Nuclear charges
*γ*: First-order reduced density matrix
*Γ*: Two-particle reduced density matrix
*λ*: Coupling coefficient in adiabatic connection
*μ*: Chemical potential
*ε*: Orbital energy
*ζ*: Spin polarization
*Φ*: Slater determinant
*ϕ*: Orbital
*Π*: Pair density
*ρ*: Density
*ρ*_*Ψ*_: *ρ* given by *Ψ*
*σ*: Spin index
*ξ*: Ensemble weight
*Ψ*: *N*-Electron wave function
*η*: Chemical hardness
*χ*: Linear response function
*ω*: Electrophilicity
